# Discovery of plasma biomarkers for colorectal cancer diagnosis via untargeted and targeted quantitative metabolomics

**DOI:** 10.1002/ctm2.805

**Published:** 2022-04-07

**Authors:** Maoqing Wang, Zhiping Long, Weinan Xue, Chenghai Peng, Tianming Jiang, Jingshen Tian, Hongru Sun, Yu Gao, Yue Yu, Yanming Yu, Chen Gong, Fan Wang, Junde Zhou, Yashuang Zhao

**Affiliations:** ^1^ National Key Disciplines of Nutrition and Food Hygiene Department of Nutrition and Food Hygiene School of Public Health Harbin Medical University, Harbin, Heilongjiang Province China; ^2^ Department of Epidemiology School of Public Health Harbin Medical University, Harbin, Heilongjiang Province China; ^3^ Department of Colorectal Surgery The Affiliated Tumor Hospital of Harbin Medical University, Harbin, Heilongjiang Province China; ^4^ Department of Emergency The Fourth Affiliated Hospital of Harbin Medical University, Harbin, Heilongjiang Province China; ^5^ Department of General Surgery The Second Affiliated Hospital of Harbin Medical University, Harbin, Heilongjiang Province China

**Keywords:** colorectal cancer, diagnosis, metabolic biomarkers, plasma

Dear Editor,

Colorectal cancer (CRC) is a major cause of cancer‐related mortality worldwide.^1^ Shifting the detection of CRC to earlier stages via massive screening has considerably reduced mortality.^2^ Metabolomics has shown great potential in the identification of noninvasive biomarkers of CRC. However, the huge number and inconsistent reports of putative markers make choosing the most appropriate biomarkers difficult. The aim of this study was to identify the metabolic markers of CRC by a multi‐step strategy. We first identified and verified differential plasma metabolites of CRC by a two‐stage case–control design. The tumour specificity of plasma markers was then confirmed by comparison with tumor‐adjacent non‐malignant paired tissue. Moreover, we conducted a systematic review of metabolomics studies of CRC to affirm the markers in multiple populations. Finally, the metabolites were quantitatively evaluated in an independent case–control population (Figure 1).

**FIGURE 1:**
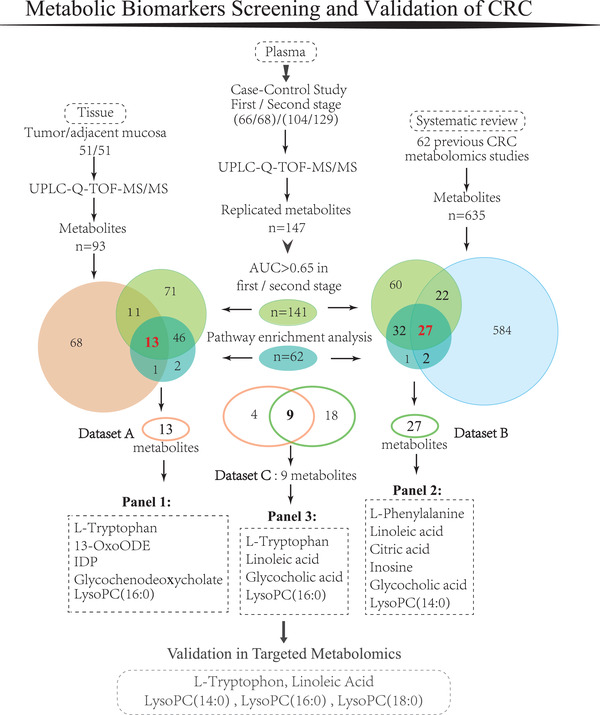
Schematic of the study design. Among the 147 replicated differential metabolites identified from this two‐stage case–control study, 141 metabolites had an AUC of > 0.65 in the first or second stage, and 62 metabolites were enriched in six biological pathways. Fifty‐nine metabolites were shared across the 141 metabolites with an AUC of > 0.65 and the 62 metabolites involved in the biological pathways. In total, 93 metabolites were identified from the metabolic profiling analysis of paired tissue. Among the 59 plasma metabolites, 13 were reproduced in the tumour tissue study (Dataset A) and 27 had been reported as differential metabolites in previously published studies (Dataset B). Nine metabolites common to Datasets A and B were grouped as Dataset C

A two‐stage case–control study involving 170 cases and 197 controls was performed. All patients were diagnosed at the Third Affiliated Hospital of Harbin Medical University. Controls were recruited from patients in the orthopaedic and ophthalmology departments and volunteers from Xiangfang District of Harbin City during the same period. Fasting peripheral venous blood was obtained in the morning in the hospital or medical examination centre. Metabolic profiling analysis was conducted on a ultra performance liquid chromatography (UPLC)/Q–time‐of‐flight (TOF)–mass spectrometry (MS)/MS platform. Principal component analysis and orthogonal projections to latent structures discriminant analysis were performed to check the separation tendency. Student's *t*‐test or Wilcoxon's rank‐sum test with an adjusted *P*‐value was applied to test the metabolites between cases and controls (details in Supporting Information, Section 1.1).

No significant differences in age, sex or body mass index were observed between the cases and controls in either the first or second stage (Table S1). The datasets with 5394 and 7312 variables in electrospray ionization in negative and positive ion mode (ESI^−^ and ESI^+^) in the first stage, and 6630 and 7760 variables in ESI^−^ and ESI^+^ in the second stage, respectively, were imported for multivariate statistical analyses. By excluding ion fragments from the same parent ions and the duplicated variables in ESI^−^ and ESI^+^, 388 variables were selected after univariate analysis. Finally, 147 metabolites were identified. Pathway‐enrichment analysis was conducted using a hypergeometric test, and six pathways (including 62 of the 147 metabolites) were enriched (Supporting Information, Section 2.1.1). Eleven of the 147 metabolites were introduced into binary logistic regression because of an area under the curve (AUC) of > 0.85, and 4 showed statistical significance [eicosenoic acid, alpha‐*N*‐phenylacetyl‐l‐glutamine, 3a,7a‐dihydroxycholanoic acid, and LysoPC(16:1(9Z))]. The combined AUCs [first‐stage AUC: 1.000, 95% confidence interval (CI): 1.000–1.000; second‐stage AUC: 0.989, 95% CI: 0.980–0.999] were higher than the AUC of any individual marker. However, all markers except LysoPC(16:1(9Z)) failed to be enriched in any biological pathway. Small‐molecule metabolites perform biological functions in pathways. Our findings indicated that these three metabolites have unknown biological function or may not be functional. Thus, although these metabolites exhibited the greatest efficacy in distinguishing CRC cases, this lack of biological significance may result in false‐positive diagnoses.

Pavlova and Thompson^3^ suggested that there are some common features of cancer cell metabolism. Although few tumours display all of these hallmarks, most display several.^4^, ^5^ Thus, no single metabolic pathway can fully reflect tumorigenesis‐associated metabolic alterations. One or a few metabolites from a single metabolic pathway also have limited diagnostic capacity. Choosing a group of metabolites encompassing a particular combination of hallmarks may ultimately improve diagnosis and tumor classification. Fifty‐nine metabolites shared across the 141 metabolites with an AUC of > 0.65 and the 62 metabolites involved in the six biological pathways were analysed. Six of the 59 metabolites [2‐aminobenzoic acid, 13‐OxoODE, citric acid, 2′‐deoxyinosine triphosphate, taurocholic acid, and LysoPC(16:1(9Z)] were focused on because they had the highest AUCs in each pathway. The combined AUCs were 0.982 (95% CI: 0.962–1.000) and 0.974 (95% CI: 0.956–0.992) in the two‐stage study (Figure 2B).

**FIGURE 2:**
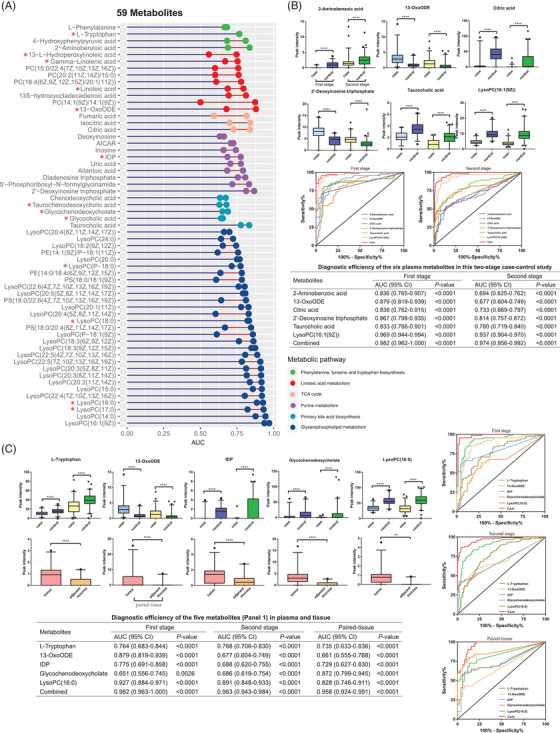
Differential metabolites with favourable diagnostic efficiency, biological significance, and tumor tissue reproducibility of colorectal cancer in a Chinese population. (A) Cleveland dot plot showing the 59 differential metabolites with favourable diagnostic efficiency and biological significance. The two dots per metabolite indicate the AUCs in the first and second stages. The red stars before the metabolite names indicate that these metabolites were also identified in the paired tissue study. (B) Box plots showing the ion intensities of the six metabolites in the first and second stages. **P < 0.01, ****P < 0.0001. AUCs of the six metabolites [2‐aminobenzoic acid, 13‐OxoODE, citric acid, 2′‐deoxyinosine triphosphate, taurocholic acid, and LysoPC(16:1(9Z))] and a multi‐marker panel in the first and second stages. (C) Box plots show ion intensities of the five metabolites (Panel 1) in both the first and second stages. **P < 0.01, ****P < 0.0001. AUCs of the five metabolites [l‐tryptophan, 13‐OxoODE, IDP, glycochenodeoxycholate, and LysoPC(16:0)] and a multi‐marker panel in the first and second stages

Blood metabolites reflect the global metabolic perturbation throughout the body. However, the plasma metabolites replicated in targeted organ tissues represent the specific metabolic characteristics of tumour cells. Thus, tissue samples of the deepest infiltration of the tumour and the adjacent non‐malignant mucosal tissues were analysed (Supporting information, Section 1.1.2). Ninety‐three differential metabolites were identified (Supporting information, Section 2.1.2). Thirteen with an AUC of > 0.65 in plasma were overlapped in tissue (Dataset A). Five of the 13 [l‐tryptophan, 13‐OxoODE, IDP, glycochenodeoxycholate, and LysoPC(16:0)] were selected as CRC panel 1 because they had the highest AUCs in the pathways (Figure 2C). The combined AUCs were 0.982 (0.963–1.000) and 0.963 (0.943–0.984) in the two‐stage study in plasma and 0.958 (0.924–0.991) in paired tissue.

Metabolomics results may be affected by several factors, including the testing coverage, sensitivity of analytical instruments, sample pretreatment method, and data analysis process.^6^ Metabolites are sensitive to many endogenous pathologies and external stimuli,^7^, ^8^ such as dietary, environmental or gut microbial factors.^9^ A systematic review is a method to collect a complete summary of the current evidence relevant to a research question.^10^ Hence, it facilitates assessment of metabolic markers with reproducibility in diverse laboratories and populations. Sixty‐two metabolomics studies of CRC were reviewed, and 635 differential metabolites were extracted (Supporting information, Sections 1.2 and 2.2). Twenty‐seven of the 635 metabolites that had an AUC of > 0.65 and were enriched in six biological pathways were designated Dataset B. Six metabolites [l‐phenylalanine (reported frequency = 20), linoleic acid (8), citric acid (9), inosine (4), glycocholic acid (3), and LysoPC(14:0) (3)] were selected when focusing on the reported frequency. The combined AUCs were 0.971 (0.949–0.994) and 0.948 (0.921–0.976) in plasma (CRC panel 2, Figure 3). Four metabolites [l‐tryptophan, linoleic acid, glycocholic acid, and LysoPC(16:0)] were finally identified from the overlap of Datasets A and B (Dataset C) because they had multiple advantages of favourable AUCs, biological significance, tumor tissue reproducibility, and the highest reported frequency (Supporting information, Sections 1.3 and 2.3). The AUCs were 0.942 (0.901–0.983) and 0.937 (0.906–0.968) in plasma. In particular, the AUCs were > 0.93 in the diagnosis of stage I/II (Figures S14 and S15).

**FIGURE 3:**
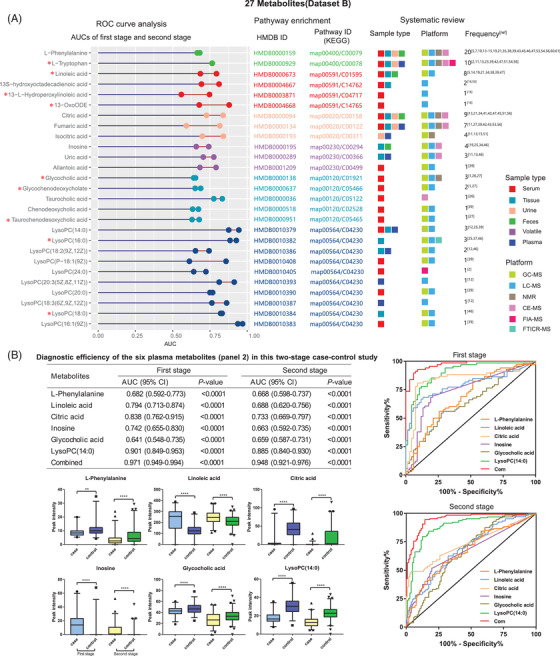
Differential metabolites with favourable diagnostic efficiency, biological significance, and external reproducibility for colorectal cancer in diverse populations. (A) Cleveland dot plot of the 27 metabolites (Dataset B). The two dots per metabolite indicate the AUCs in the first and second stages. The red stars before the metabolite names indicate that these metabolites were also identified in the paired tissue study. The reference numbers (ref) of studies are the same as those in Table S14. (B) Box plots showing ion intensities of the six metabolites (Panel 2) in both the first and second stages. **P < 0.01, ****P < 0.0001. AUCs of the six metabolites [l‐phenylalanine, linoleic acid, citric acid, inosine, glycocholic acid, and LysoPC(14:0)] and a multi‐marker panel in the first and second stages

Ultimately, we quantified five metabolites [l‐tryptophan, linoleic acid, LysoPC(14:0), LysoPC(16:0), and LysoPC(18:0)] in the targeted analysis. A batch of 504 plasma samples (251 patients with CRC and 253 controls) was detected (Supporting information, Sections 1.4 and 2.4). The AUC of l‐tryptophan, linoleic acid, LysoPC(14:0), and LysoPC(18:0) was 0.728 (0.681–0.775) in plasma (Figure 4).

**FIGURE 4:**
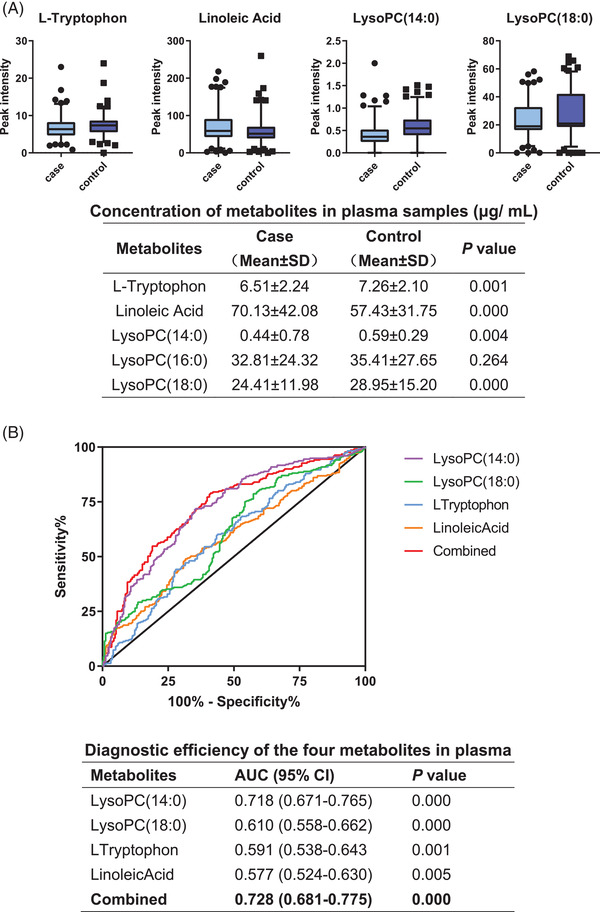
The four plasma metabolic biomarkers validated in quantitative analysis. (A) Box plots showing ion intensities of the four metabolites. (B) AUCs of the four metabolites [l‐tryptophan, linoleic acid, LysoPC(14:0), and LysoPC(18:0)] and a multi‐marker panel

This study had some limitations. First, the fact that the biomarkers were not well validated might have been due to some other parameters that were either unknown or not tightly controlled. Second, change variation was not taken as a strict standard in the screening process because we did not want to exclude metabolites that may have better sensitivity in early cancer. The response of small molecular metabolites to nutritional or disease states is so sensitive that even subtle disturbances can be detected much earlier than genomic and proteomic variation. Third, UPLC/Q–TOF–MS/MS is not a good technique for quantitative analysis. Fourth, the model discrimination performance still requires validation internally and externally using targeted quantitative analyses. Finally, comparisons with the metabolites of other diseases are still required.

In conclusion, we revealed the most promising metabolic biomarkers of CRC in plasma using the herein‐described multi‐step analysis strategy. Future application of multi‐omics markers may achieve accurate noninvasive diagnosis.

## CONFLICT OF INTEREST

The authors declare no conflict of interest.

## Supporting information

Supporting Information
