# Supplementary material for: Discovery of plasma biomarkers for colorectal cancer diagnosis via untargeted and targeted quantitative metabolomics
Source: Clin Transl Med. 2022 Apr 7;12(4):e805. doi: 10.1002/ctm2.805 (PMC8989079; doi:10.1002/ctm2.805)
Supplement: Supplementary file 1 — Supporting Information [file CTM2-12-e805-s001.pdf]

SUPPLEMENTARY MATERIAL FOR

Discovery of Plasma Biomarkers for Colorectal Cancer Diagnosis via Untargeted and Targeted Quantitative Metabolomics

List of Content

**I. Supplementary Methods**	**Page**
1.1 Metabolomics studies of plasma and paired tissue of CRC in a population from China	**1**
1.1.1 Study design and subjects recruitment	**1**
1.1.2 Experimental protocol of plasma and tissue metabolic profiling analyses	**1**
1.1.3 Data processing, multivariate and univariate analyses of metabolites, and identification of differential metabolites	**4**
1.2 Systematic review of metabolomics studies of human CRC	**5**
1.3 Plasma biomarkers screening for CRC diagnosis	**7**
1.4 Plasma biomarkers validation based on targeted metabolic assays	**8**
1.4.1 Sample and Platform selection	**8**
1.4.2 Experimental protocol of targeted plasma metabolic biomarkers analyses	**8**
1.4.3 Qualitative and quantitative method of plasma metabolic biomarkers	**9**
1.4.4 Evaluation of UPLC/Q-TOF-MS/MS detecting methodology	**10**
**II. Supplementary Results**	
2.1 Metabolomics studies of plasma and paired tissue of CRC in a Chinese population	**11**
2.1.1 Global plasma metabolic profiling analysis by UPLC/Q-TOF-MS/MS	**11**
2.1.2 Metabolic profiling analysis of paired tissue by UPLC/Q-TOF-MS/MS	**12**
2.2 Systematic review of metabolomics studies of human CRC	**12**
2.3 Plasma biomarkers screening for CRC diagnosis	**14**
2.4 Biomarkers Validation in Targeted Metabolomics	**15**
**III. Supplementary Figures**	
S.Figure 1. Representative ultra-performance liquid chromatography and quadrupole time-of-flight tandem mass spectrometry (UPLC/Q-TOF MS/MS)-based peak intensity chromatogram of human plasma	**17**
S.Figure 2. Two-dimensional PCA score plots of plasma from colorectal cancer patients, healthy controls, and QC samples	**17**
S.Figure 3-1. Global plasma metabolic profiling analysis by UPLC/Q-TOF-MS/MS of the first stage<br>S.Figure 3-2. PCA and OPLS-DA score plots of the second stage and permutation test results of OPLS-DA model	**18**
S.Figure 4. The six significantly enriched pathways by differential plasma metabolites in this two-stage case-control study	**21**
S.Figure 5. Data processing, statistical analysis, and identification of differential metabolites in plasma and tissue by UPLC/Q-TOF-MS/MS	**22**
S.Figure 6. PCA and OPLS-DA score plots of tissue from colorectal cancer patients and permutation test results of OPLS-DA model	**23**

S.Figure 7. General tendency of metabolites discriminated in paired tissue metabolomics analysis	**24**
S.Figure 8. Workflow of literature retrieve in the systematic review of metabolomics studies of colorectal cancer	**25**
S.Figure 9. Overall distribution of sample types, platforms, publication year, study design, and populations in the systematic review of metabolomics studies of colorectal cancer.	**26**
S.Figure 10. Reported frequency in previous studies of the differential metabolites with good diagnostic value in our study	**27**
S.Figure 11. Reported frequency in previous studies of the differential metabolites with biological significance in our study	**28**
S.Figure 12. Four metabolic biomarkers with the best diagnostic value of colorectal cancer in Northeastern Chinese population.	**29**
S.Figure 13. Comparison of based peak intensity chromatograms between our and previous reported methods of preparing human plasma	**30**
S.Figure 14. The optimal candidate metabolic biomarkers for colorectal cancer diagnosis in diverse populations	**31**
S.Figure 15. The diagnositic efficacy of panels in different TNM stages of colorectal cancer.	**32**
**IV Supplementary Tables**	
S.Table 1. Demographic characteristics of participants in this two-stage case-control study in a population from China.	**33**
S.Table 2. Colorectal cancer metabolites identified in the two-stage case–control study in a population from China	**34**
S.Table 3. Demographic and pathological characteristics of fifty-one colorectal cancer patients in the paired tissue metabolomics study	**42**
S.Table 4. Colorectal cancer metabolites identified from the paired tissue metabolomics study	**45**
S.Table 5. Differential metabolites and reported frequency in previous metabolomics studies of colorectal cancer	**50**
S.Table 6. Altered pathways reported in previous metabolomics study of colorectal cancer	**63**
S.Table 7. Pathway enrichment analysis of the 635 metabolites reported in 62 published metabolomics studies of colorectal cancer	**65**
S.Table 8. Diagnostic efficiency of metabolites screened out from the two-stage case-control study in a population from China	**66**
S.Table 9. Diagnostic efficiency of the eleven metabolites in a population from China	**73**
S.Table 10. Variables in the equation of binary logistic regression of eleven metabolites	**74**
S.Table 11. Diagnostic efficiency of metabolites screened out from a tumor-adjacent non-malignant paired tissue metabolomics study	**76**
S.Table 12. Candidate biomarkers and panels screened out from this comprehensive analysis for colorectal cancer diagnosis based on metabolomics studies	**79**
S.Table 13. The diagnostic efficacy of different tumor stages of the biomarker panels	**80**
S.Table 14. Characteristics of metabolomics studies of colorectal cancer included in this systematic review	**81**
S.Table 15. Quality assessment of studies included in the systematic review by QUADOMICS	**116**
S.Table 16. Limit of detection, limit of quantification and standard curve of standards	**120**
S.Table 17. Standard addition recovery and relative standard deviation (N =6)	**120**
S.Table 18. Intra-day and inter-day precision (n=6)	**121**
S.Table 19 The sensitivity and specificity of metabolites in ROC analysis	**122**

I. Supplementary Methods

Fig. 1 shows the complete schematic for this study design.

1.1 Metabolomics studies of plasma and paired tissue of CRC in a population from China

1.1.1 Study design and subjects recruitment We designed a two-stage case–control study to identify and test the specific plasma metabolites of CRC in a population from China. All the subjects of the first stage study were collected in 2007. The subjects of another independent case-control study as the second stage were collected in 2010. The first stage included 66 CRC cases and 68 controls and the second stage included 104 cases and 129 controls. A self-control study was conducted to detect differential metabolites between tumor tissue and adjacent non-malignant mucosal tissue. Fifty-one pairs of tissue were obtained from surgical resection of CRC patients in 2010. Metabolic profiling analyses of plasma and tissue were conducted by UPLC/Q-TOF-MS/MS.

All patients were diagnosed and recruited at the Third Affiliated Hospital of Harbin Medical University. Any patient with neuroendocrine carcinoma, malignant melanoma, non-Hodgkin's lymphoma, gastrointestinal stromal tumors, or Lynch syndrome CRC was excluded. Only newly diagnosed, histopathologically confirmed cases were retained. About 5 ml of fasting peripheral venous blood was obtained in the first morning of patients' admission but before surgery and any treatment (including surgery, chemotherapy or radiation therapy). Controls were recruited from the patients in the orthopedic and ophthalmology departments of the Second Affiliated Hospital of Harbin Medical University, and among volunteers from Xiangfang district of Harbin City over the same time period. Any subject in the control group with inflammatory conditions, gastrointestinal tract disorders, or a history of polyps, adenoma, or other cancer-related disease was excluded. Fasting peripheral venous blood from controls was obtained in the morning in hospital or community medical examination center. The operational procedures of sample collection, pretreatment and detection were as consistent as possible in the two-stage study

Blood samples were collected in an EDTA tube and centrifuged at 3000 rpm, 10 min and 4 ℃ within 3 h of collection, after which plasma were separated and were frozen at −80 ℃. Tissue sampling included the deepest infiltration of the tumor and the adjacent non-malignant mucosal tissues. All tissues were immediately soaked in formaldehyde solution until use.

1.1.2 Experimental protocol of plasma and tissue metabolic profiling analyses

Because the high chromatographic resolution, high sensitivity, and rapid separation, ultra-performance

liquid chromatography (UPLC) coupled to Q-TOF-MS/MS is the greatest method for rapid qualitative and quantitative analysis of subtle changes in metabolites in complex mixtures. Thus, metabolic profiling analyses of plasma and tissue were conducted by UPLC/Q-TOF-MS/MS.

Sample selection Considering the invasive procedure and limited frequency of tissue sampling, biofluids (e.g. plasma, serum, and urine) are preferred for testing. Urine contains metabolic breakdown products from a wide range of foods, drinks, drugs, and environmental contaminants, which results specialize in identifying temporal metabolic changes. By contrast, the reliability (within-person variance) and stability (shipment at different temperatures, use of gel-barrier collection tubes, freeze-thaw cycles) of metabolites in fasting serum and plasma are much better, and levels of the most targeted metabolites remain stable within a 14-day period. (Breier M, Wahl S, Prehn C, et al. Targeted metabolomics identifies reliable and stable metabolites in human serum and plasma samples. PLoS One 2014;9:e89728.) Therefore, we chose blood-derived metabolites as the cancer biomarkers.

Chemicals and reagents Acetonitrile and methanol (HPLC grade) were purchased from Honeywell Burdick & Jackson (Muskegon, MI). Formic acid was purchased from Beijing Reagent Company (Beijing, China). All chemicals and regents were of HPLC grade available from commercial sources. Ultrapure water was prepared by an ultra-clear system (PURELAB Ultra; Veolia Water Solutions & Technologies, France).

Plasma sample preparation Plasma samples were thawed at 4 ℃, subjected to vortexing for 1 min, placed in a 2-ml centrifuge tube at a volume of 250 μl and supplemented with 750 μl of methanol, vortexed for 1 min, and then centrifuged at 12,000 rpm for 10 min at 4 ℃. The supernatants were then obtained and dried under nitrogen, after which a 350-μl mixture of acetonitrile and water (1:2, v/v) was used to wash the precipitated proteins to decrease the loss of the polar materials in plasma. They were then vortexed for 1 min and centrifuged at 12,000 rpm for 10 min at 4 ℃. The obtained supernatants were transferred to the previous nitrogen-dried tubes to dissolve the residue, vortexed for 1 min, and stewing for 5 min, followed by centrifugation at 12,000 rpm for 10 min at 4 ℃. The supernatants were then transferred to autosampler vials for metabolomic analysis. Aliquots of 10 individual plasma samples from 5 CRC patients and 5 controls were combined to make a typical pooled quality-control (QC) sample to verify the reproducibility and reliability of the data. The QC sample was analyzed every 15th sample throughout the analytical run.

Tissue sample preparation About 100 mg of tissue soaked in formaldehyde solution was placed in a mortar containing Liquid nitrogen and fully grounded. After the well-ground tissue was completely dissolved with 2 ml of methanol, all metabolites in the tissue were extracted and all proteins were precipitated. The solution obtained from step 1 was transferred to a 2 ml centrifuge tube, then was vortexed for 1 min; and centrifuged at 12,000 rpm for 10 min at 4℃. The supernatant was put in another 2 ml centrifuge tube and was dried with nitrogen. The substance dried by nitrogen in step 3 was dissolved with methanol (1:1) and the tissue soaking solution (300-400ul). Vortexed for 1 min and standing for 5 min, then centrifuged at 12,000 rpm for 10 min at 4℃. The supernatant was finally removed and transferred to auto sampler vial for metabolomics analysis by ULPLC/Q-TOF MSMS. A quality control sample (QC) was prepared by mixing aliquots from all supernatant samples (10 μL from each sample).

Optimization for the protocol of sample preparation The coexistence of both polar (e.g., amino acids and sugars) and nonpolar (e.g., lipids) analytes means that serum/plasma represents a rather more difficult challenge for sample preparation. Most previous studies applied precipitation with a high solvent-to-sample ratio (e.g., 3:1 v/v), with either methanol- or acetonitrile-based systems, followed by centrifugation. While efficient protein removal can be achieved, but most polar metabolites in the serum/plasma may be lost. We optimized the metabolite extraction protocol in our study to extract both polar and nonpolar metabolites *via* two consecutive extractions. After protein precipitation, the protein deposit was solubilized in a solvent with high polarity (1:2 acetonitrile/water). Then, another centrifugation was used to collect the polar metabolites for metabolite profiling analysis. This second extraction approach avoided the loss of polar metabolites and achieved relatively complete extraction of all metabolites from plasma. This protocol was used in our metabolomics studies; we got more differential metabolites (see the comparison in S.Figure 13) than the conventional methods used in previous studies.

UPLC/Q-TOF-MS/MS analysis Chromatographic separation was performed on a 1.7-μm BEH C18 column [ACQUITY (HSS); Waters Corp., Milford, MA, USA; 2.1 mm × 100 mm] equipped with a UPLC system (ACQUITY UPLC; Waters Corp., USA). The temperatures of the column and autosampler were maintained at 35℃ and 4℃, respectively. A sample (2 μl) of the preprocessed plasma was injected onto the column at a flow rate of 0.35 ml/min. The mobile phase consisted of

water containing 0.1% formic acid waters (solution A) and acetonitrile (solution B). The elution gradient was as follows: 2% B for 0.5 min; 2% to 20% B over 0.5 to 6.0 min; 20% to 35% B over 6.0 to 7.0 min; 35% to 70% B over 7.0 to 9.0 min; 70% to 98% B over 9.0 to 10.5 min; 98% B for 2.0 min; and then a return to 2% B for 6.0 min. Once the initial settings had been established, the column was equilibrated for 2.0 min. Acetonitrile was run every fifth sample as a blank solution and the plasma samples in the two analysis batches were injected alternately as five cases and five control samples.

Q-TOF MS/MS was performed with a mass spectrometer (Micromass Q-TOF mass spectrometer; Waters Corp., Manchester, UK) using an electrospray ionization (ESI) interface operated in both ion modes (ESI^- and ESI^+). The analytical parameters were as follows: capillary voltage, 2800 V in ESI^- or 3000 in ESI^+; sample cone voltage, 35 V; collision energy, 6 eV; source temperature, 110 ℃; desolvation gas (nitrogen) flow, 650 L/h; desolvation temperature, 320 ℃; cone gas (nitrogen) flow, 50 L/h; collision gas, argon; and MCP detector voltage, 2400 V. The Q-TOF mass acquisition rate was set at 0.4 s, with an interscan delay of 0.1 s. The scan mass range was from 50 to 1000 m/z. The data were collected in centroid mode, using the lock spray to ensure accuracy and reproducibility. A concentration of 200 pg/ml leucine–enkephalin was used as lock mass (m/z 554.2615) in ESI^- and (m/z 556.2771) in ESI^+. The lock spray frequency was set at 10 s, and the lock mass data were averaged over 10 scans for correction. The MS/MS spectra of metabolites were obtained by UPLC-MS/MS.

1.1.3 Data processing, multivariate and univariate analyses of metabolites, and identification of differential metabolites

Data processing The raw data were imported into MarkerLynx software incorporated in Masslynx software (version 4.1 SCN714). MarkerLynx ApexTrack peak integration was used for peak detection and alignment. The ApexTrack peak parameters were set as follows: peak width at 5% height, 1 s, and peak-to-peak baseline noise (calculated automatically). Collection parameters were set as follows: retention time (RT) range 0.5–10.5 min, mass range 50–1000 Da, mass tolerance, 0.05 Da; RT tolerance, 0.1 min; minimum intensity, 80; noise elimination level, 6.0; and deisotope data, yes. After being recognized and aligned, the intensity of each ion was normalized to the summed total ion intensity of each chromatogram. The data-reduction process was handled in accordance with the "80% rule".

Multivariate analysis of metabolites A matrix of samples against variables was generated and

transferred to SIMCA-P version 13.0 software (Umetrics, Umeå, Sweden) for multivariate analysis. Principal component analysis (PCA) was first performed to check the outliers and the separation tendency. Orthogonal projections to latent structures discriminant analysis (OPLS-DA) was further applied to visualize the maximal difference between cases and controls. A default sevenfold (leave one-seventh of samples out) cross-validation procedure was used to assess the robustness of the models. Furthermore, permutation tests calculated by 100 randomizations were performed to avoid the overfitting of supervised OPLS-DA models. The variable importance in projection (VIP >1.5 and P <0.05) values of all peaks from the OPLS-DA model was taken as a coefficient for peak selection.

Univariate analysis of metabolites Student's *t*-test or Wilcoxon rank-sum tests were used for two-sample tests of metabolites between cases and controls based on normality test. An adjusted *P*-value (FDR correction) was applied to protect against multiple comparison problem. The cut-off (0.05) was considered statistically significant. Both first and second stages were analyzed using the same multivariate and univariate statistical analyses as described above.

Differential metabolites identification Metabolite annotation was performed by comparing the exact m/z values and MS/MS spectra with those in free online databases, including Human Metabolome Database (HMDB, http://www.hmdb.ca/), Metlin (http://metlin.scripps.edu), and LMSD (http://www.lipidmaps.org/). If the potential MS/MS spectra were not available in online databases, the MassFragment application manager (MassLynx version 4.1, Waters) was applied to facilitate the MS/MS fragment ion analysis process via chemically intelligent peak-matching algorithms. Differential metabolites were finally confirmed using standard compounds based on both retention time and MS/MS spectra.

Statistics Categorical variables were tested by chi-square test, and continuous variables were tested by two-sample t test for demographic information between cases and controls. Variables with statistically significant difference were adjusted as confounders. Heatmaps were generated to describe the general tendency of metabolites from plasma and paired tissue metabolomics analyses. The log2 fold-change of per-metabolites in cases relative to controls was calculated. Receiver operating characteristic (ROC) curve, sensitivity, and specificity were used to assess diagnostic values. A *P*-value<0.05 was considered statistically significant for ROC tests.

1.2 Systematic review of metabolomics studies of human CRC

To comprehensively collect the metabolites of CRC reported in various platforms and populations, we conducted a systematic review of metabolomics studies of human CRC and extracted all differential metabolites reported in original metabolomics studies. We counted the reported frequency as the judging basis of reproducibility. Pathway enrichment analysis was conducted based on all the differential metabolites extracted from previous studies. We also extracted the biological pathways of CRC that were reported in original studies.

Literature search strategy We systematically reviewed three databases (PubMed, Embase, and ScienceDirect) to identify all metabolomics studies of CRC reported from 1998 through June 2018. The following MeSH terms and keywords were used: "metabolomics," "metabonomics," "NMR," "GC-MS," "LC-MS," and "colorectal cancer." We attempted to collect eligible studies by computer retrieval combined with a manual search of bibliographies. The references were also scrutinized to identify further relevant publications.

Inclusion and exclusion criteria Only original research involving global metabolic profiling analysis and biomarker discovery of CRC was included. Additional inclusion criteria were as follows: 1) reports published in English; 2) studies with case–control design, nested case–control design, or self-control in patients (Studies detecting tumor tissue commonly used autologous non-tumorous adjacent tissue specimens as matched controls were classified to self-control studies.); 3) studies investigating either human tissues or body fluids; 4) the use of a detection platform including NMR, GC-MS, LC-MS, UPLC-MS/MS, HPLC-GC/MS-MS, or GC-TOF; and 5) the names of differential metabolites or significantly altered pathways being available to extract. All studies involving in vitro experiments or animal models were excluded.

Quality assessment of individual studies QUADOMICS, a quality assessment tool for assessing diagnostic accuracy, was adopted to assess the methodological quality of individual studies. This tool has contributed to the development of specific recommendations on '-omics'-based diagnostic research. The quality of analyzed studies was summarized by the percentage of applied criteria for which a positive score was assigned. Since no clear threshold value has been published by either QUADAS or QUADOMICS, it was not possible to assess studies using a single cut-off value to identify flaws in the methodological designs. *(Lumbreras B, Porta M, Marquez S et al. QUADOMICS: an adaptation of the Quality Assessment of Diagnostic Accuracy Assessment (QUADAS) for the evaluation of the*

methodological quality of studies on the diagnostic accuracy of '-omics'-based technologies. Clin Biochem 2008; 41: 1316-1325.)

Data extraction from included studies The following information was extracted from each included report: 1) basic information on the publication, such as the name of the first author, publication year, and study design; 2) numbers of CRC patients and controls; 3) matching status and matching conditions; 4) type of biospecimens; 5) platform for metabolomic analysis; 6) metabolites differentiating CRC patients from controls; and 7) significantly altered pathways. Two researchers (J.S. Tian and Z.P. Long) extracted the above-mentioned information independently using standard forms specifically created for this systematic review. Any disagreement was resolved by discussions with other researchers (F. Wang and M.Q. Wang) or a group meeting.

1.3 Plasma biomarkers screening for CRC diagnosis

Firstly, all replicated differential metabolites from the two-stage case-control study were selected and were processed for following ROC analysis. According to the areas under ROC curves (AUCs), a list of metabolites with AUC>0.85 in both the first and second stages in the Chinese population was sorted. A logistic regression model was established to filter the variable with the most important contribution on cases discrimination by a backward statistical technique.

Secondly, all replicated differential metabolites from the two-stage case-control study were processed for pathway enrichment analyses. MetaboAnalyst 3.0[33] incorporated with database sources including KEGG (http://www.genome.jp/kegg/) and HMDB (http://www.hmdb.ca/) was applied for pathway enrichment analysis. Only the metabolites enriched in pathways were considered as having biological significance. Thus, the metabolites significantly enriched in relevant biological pathways and also with good diagnostic value (AUC>0.65 in the first or second stage) were selected. Moreover, metabolites with the highest AUCs from each pathway were grouped together as a panel.

Thirdly, we cross checked the replicated metabolites from plasma and paired tissue metabolomics analysis in the Chinese population. Only the metabolites with tumor tissue reproducibility were selected (Fig. 1).

Finally, differential metabolites with favorable diagnostic values (AUC>0.65 in the first or second stage), biological significance (enriched in pathways), and further confirmation in CRC tumor tissue were selected as Dataset A. The metabolites that showed the highest AUCs in each pathway from

Dataset A were selected as CRC Panel 1. Metabolites with AUC>0.65 in the first or second stage, involved in relevant biological pathways, and were also reported in external populations studies as differential metabolites of CRC were selected as Dataset B. Metabolites with the highest reported frequencies in each pathway from Dataset B were selected as CRC Panel 2 (if two or more metabolites had the same reported frequency, the one with the higher AUC was selected.). The replicated metabolites between Dataset A and B were selected as Dataset C. Finally, synthesizing all the factors, including external reported frequency, reproducibility in tumor tissue, biological significance, and AUC values in the two-stage case-control study, CRC Panel 3 were screened out.

1.4 Plasma biomarkers validation based on targeted metabolic assays

1.4.1 Sample and Platform selection

A total of 251 patients with CRC diagnosed by hospital pathology between October 2016 and December 2019 were enrolled in the quantative validation set. The source and collection criteria of the participants of the case and control are the same as described above (SM 1.1.1). Targeted metabolomic analyses of plasma were conducted by UPLC/Q-TOF-MS/MS. Methodological evaluation of targeted quantification of metabolites is shown in S.Table 16-18.

1.4.2 Experimental protocol of targeted plasma metabolic biomarkers analyses

Chemicals and reagents Metabolites' standards and internal standards, including L-Tryptophon, Linoleic Acid, LysoPC(14:0), LysoPC(16:0), LysoPC(18:0), were purchased from Shanghai Zhenzhun Biotechnology (Co., Ltd.).

Plasma sample preparation Plasma samples were thawed at 4 ℃, subjected to vortexing for 1 min, placed in a 1.5-ml centrifuge tube at a volume of 100 μl and supplemented with 300 μl of methanol, vortexed for 2 min, and then centrifuged at 12,000 rpm for 10 min at 4 ℃. The supernatants were then obtained and dried under nitrogen, after which a 140-μl mixture of acetonitrile and water (1:1, v/v) was used to wash the precipitated proteins to decrease the loss of the polar materials in plasma. They were then vortexed for 2 min and centrifuged at 12,000 rpm for 10 min at 4 ℃. The obtained supernatants were transferred to the previous nitrogen-dried tubes to dissolve the residue, vortexed for 2 min, and stewing for 10 min, followed by centrifugation at 12,000 rpm for 10 min at 4 ℃. The supernatants were then transferred to autosampler vials for metabolomic analysis. Aliquots of 10 individual plasma samples from 5 CRC patients and 5 controls were combined to make a typical pooled quality-control

(QC) sample to verify the reproducibility and reliability of the data. The QC sample was analyzed every 15th sample throughout the analytical run.

UPLC/Q-TOF-MS/MS analysis Chromatographic separation was performed on a 1.7-μm BEH C18 column [ACQUITY (HSS); Waters Corp., Milford, MA, USA; 2.1 mm × 100 mm] equipped with a UPLC system (ACQUITY UPLC; Waters Corp., USA). The temperatures of the column and autosampler were maintained at 35°C and 4°C, respectively. A sample (4 μl) of the preprocessed plasma was injected onto the column at a flow rate of 0.35 ml/min. The mobile phase consisted of water containing 0.1% formic acid waters (solution A) and acetonitrile (solution B). The elution gradient was as follows: 2% B for 0.5 min; 2% to 30% B over 0.5 to 2.0 min; 30% to 70% B over 2.0 to 3.0 min; 70% to 75% B over 3.0 to 7.5 min; 85% to 98% B over 7.5.0 to 10.5 min; 98% B for 2.0 min; and then a return to 2% B for 6.0 min. Once the initial settings had been established, the column was equilibrated for 2.0 min. Acetonitrile was run every fifth sample as a blank solution and the plasma samples in the two analysis batches were injected alternately as five cases and five control samples.

Q-TOF MS/MS was performed with a mass spectrometer (Micromass Q-TOF mass spectrometer; Waters Corp., Manchester, UK) using an electrospray ionization (ESI) interface operated in negative ion modes (ESI^-). The analytical parameters were as follows: capillary voltage, 2800 V in ESI^-; sample cone voltage, 35 V; source temperature, 125℃; desolvation gas (nitrogen) flow, 720 L/h; desolvation temperature, 320℃; cone gas (nitrogen) flow, 12 L/h. The Q-TOF mass acquisition rate was set at 0.48s. The scan mass range was from 50 to 1000 m/z, and time range was from 0 to 16min. The data were collected in centroid mode, using the lock spray to ensure accuracy and reproducibility. A concentration of 200 pg/ml leucine–enkephalin was used as lock mass (m/z 554.2615) in ESI^-. The lock spray frequency was set at 10 s, and the lock mass data were averaged over 10 scans for correction. The MS/MS spectra of metabolites were obtained by UPLC-MS/MS.

1.4.3 Qualitative and quantitative method of plasma metabolic biomarkers

The software Masslynx was used to process the mass spectrum data. The standard solutions of 5 substances were scanned by the primary mass spectrometer to obtain the retention time and quasi-molecular ion peak of each substance; in the MS/MS mode, the secondary mass spectra of each substance were obtained by optimizing the corresponding collision energy. Choose two The most abundant fragment ion is used as the qualifier ion. When analyzing a serum sample, the substance to be

tested can be accurately characterized by comparing with the retention time, quasi-molecular ion peak and fragment ion of the standard.

The standard stock solutions of 5 substances are diluted with methanol and prepared into standard solutions of different mass concentrations. The peak areas of the quasi-molecular ions of different concentrations of substances are the ordinate (Y), and the corresponding concentration is the abscissa (X) , Draw the standard curve of 5 kinds of substances respectively, analyze the content of 5 kinds of substances in the serum.

1.4.4 Evaluation of UPLC/Q-TOF-MS/MS detecting methodology

Detection limit and quantitation limit A series of mixed standard solutions of 5 kinds of standard substances were prepared, and the concentrations were detected from low to high. The limit of detection (LOD) and limit of quantification (LOQ) of the target substances were determined by the signal-to-noise ratio S/N= 3 and S/N=10, respectively.

Standard addition recovery, precision determination A certain amount of mixed standard solution was added to the serum sample to evaluate the standard addition recovery of the method. The mixed standard solution of 5 substances of low, medium and high concentration (1, 5 and 10 ug /mL) was added to the serum sample, and 6 parallel samples were made for each concentration. Sample preparation and quantitative analysis were performed according to the previous method, and the concentration after addition was calculated. In addition, the serum samples without standard solution were measured 6 times in parallel to calculate the unlabeled concentration. The recovery rate was calculated according to the formula: recovery rate = (spiked concentration - unspiked concentration)/spiked concentration ×100%.

Six samples were processed at low, medium and high concentrations (1,5,10 ug /mL) in parallel, and the relative standard deviations (RSD) of the measured concentrations were calculated respectively to evaluate the intraday precision of the detection of each target substance at each concentration level. The RSD (n=6) of the concentration of each target was calculated and the precision of the method was evaluated.

II. Supplementary Results

2.1 Metabolomics studies of plasma and paired tissue of CRC in a Chinese population

2.1.1 Global plasma metabolic profiling analysis by UPLC/Q-TOF-MS/MS

Demographics of study population in this two-stage case-control study No significant differences in age, gender, or body mass index (BMI) were observed between the cases and controls in either the first or second stage (S.Table 1).

Quality assessment of the metabolomics platform The typical based peak intensity (BPI) chromatograms of plasma revealed that gradient elution accurately distinguished metabolites between the samples from cases and controls (S.Figure 1). Principal Components Analysis (PCA) was applied to obtain an initial overview of the quality of the analytical run. Quality control (QC) results were all clustered tightly in the middle of the score plot in both ion modes of two-stage case-control detection (S.Figure 2). The relative standard deviations (RSD; %) of the retention time and peak area ranged from 0 to 0.73 and 0.8 to 4, respectively in the intra-batch assay, and ranged from 0.1 to 2.7 and 1.5 to 6.2, respectively in the inter-batch assay. The results showed that the stability of the UPLC/Q-TOF MSMS platform was excellent throughout the run, and was sufficient to ensure data quality for further global metabolic profiling analyses.

Multivariate analysis for plasma metabolic profiling analysis The datasets with 5394 and 7312 variables in electrospray ion source^{-} (ESI^{-}) and ESI^{+} in the first stage, and 6630 and 7760 variables in ESI^{-} and ESI^{+} in the second stage, respectively, were imported for multivariate statistical analyses. As shown in the PCA score plot, CRC samples were generally distinct from healthy controls (S.Figure 3). R^2Y and Q^2 values to the left were lower than the original point to the right, which strongly suggested the validity and accuracy of the current models. Based on these results, we confirmed that there were significant alterations in the plasma metabolites of CRC patients compared with healthy controls.

Univariate analysis and metabolites identification A total of 284 and 382 variables were selected in ESI^{-} and ESI^{+} in the first stage, while 455 and 398 variables were selected in ESI^{-} and ESI^{+} in the second stage, respectively. In total, 213 and 210 variables were shared across the two datasets in ESI^{-} and ESI^{+}. By excluding ion fragments from the same parent ions and the duplicated variables in ESI^{-} and ESI^{+}, 388 variables were selected among the 213 and 210 shared variables; and a total of 147 metabolites were finally identified. Detailed annotation for the identification of each metabolite was

given in S.Table 2.

Heatmap and fold change of the identified differential metabolites Compared with healthy controls, 65 metabolites were significantly upregulated, whereas 82 metabolites were significantly downregulated in CRC patients. There were 141 metabolites with AUC>0.65 and 70 metabolites with log2 fold-change≥2 or ≤−2 (S.Figure 3-1).

Pathway enrichment analysis Six pathways were significantly enriched, namely, linoleic acid metabolism; purine metabolism; phenylalanine, tyrosine, and tryptophan biosynthesis; primary bile acid biosynthesis; TCA cycle; and glycerophospholipid metabolism (S.Figure 4). Sixty-two metabolites involved in these six pathways were considered to have biological significance among the 147 differential metabolites.

2.1.2 Metabolic profiling analysis of paired tissue by UPLC/Q-TOF-MS/MS

Demographic and pathological characteristics of CRC patients S.Table 3 shows the demographic and clinicopathologic characteristics of 51 CRC patients. There were 23 female and 28 male patients included in this tumor-adjacent non-malignant paired tissue metabolomics study. According to the TNM staging system, four patients were stage IV, 27 patients were stage III (IIIA, 10; IIIB, 17), 15 were stage II (IIA, 3; IIB, 12), and two patients were stage I.

Multivariate analysis for tissue metabolic profiling analysis Although PCA did not show a perfect separation between tumor and adjacent non-malignant mucosal tissues, OPLS-DA models achieved a distinct cluster separation. To avoid over fitting, a permutation test was conducted to assess the risk of OPLS-DA model (S.Figure 6-7). R^2Y and Q^2 values indicated the validity and accuracy of the current models.

Univariate analysis of metabolites In total, 167 and 315 variables were selected in ESI^- and ESI^+, respectively. A total of 93 metabolites were identified by comparing retention time and precise MS (S.Figure 5, S.Figure 7b).

Heatmap and fold change of identified differential metabolites Compared with adjacent mucosal tissue, 80 metabolites were upregulated, and 13 were downregulated in tumor tissue. There were 57 metabolites with log2 fold-change≥2 or ≤−2 (S.Figure 7, S.Table 4).

2.2 Systematic review of metabolomics studies of human CRC

Literature retrieval The workflow of literature selection is shown in S.Figure 8. A total of 954

articles were retrieved, primarily from the PubMed, Embase, and ScienceDirect online databases. Among the 954 studies, 386 were excluded for duplicated records; 73 were excluded owing to the report not being written in English, or the study being a review or only abstract available; and 333 were excluded owing to cell line experiment or animal research; as well as the involvement of proteomic studies. A further 109 articles were excluded owing to inconsistency in the research purpose, including studies focused on metabolites associated with survival prediction and therapy evaluation. Additional 7 eligible articles were retrieved from the reference lists and reviews manually. Finally, 62 articles were included in this systematic review.

Description of included studies The information of all eligible studies is presented in S.Table 14. Among the 62 included studies, 5 (5/62, 8.06%) studies were nested case–control studies, 36 (36/62, 58.06%) studies had a case–control design, and 21 (21/62, 33.87%) studies had a self-control design of paired tissue metabolomics study. The participants in 37 studies were from Asia (China, Japan, South Korea, Singapore, and Iran), those in 13 studies were from Europe (Latvia, France, Spain, Italy, Germany, Norway, UK, and Denmark), and those in 13 studies were from North America (USA). Six studies did not provide matching information in detail.

Sample types and platforms A variety of biological samples were analyzed to identify metabolic biomarkers of CRC in the 62 included metabolomics studies, including tissue (n=23 studies), serum (n=18), urine (n=7), feces (n=8), plasma (n=5), volatiles from blood (n=1) and exhaled breath (n=2), and dried blood spot (DBS) (n=1). Different platforms were also applied among these 62 studies: 4 used CE-MS, 23 used GC-MS, 11 used LC-MS, 14 used NMR, and 10 used multiple platforms (S.Figure 9).

Quality assessment of eligible studies Based on the QUADOMICS tool, 56 out of 62 (90.32%) studies were not able to avoid overfitting owing to the lack of an independent validation set. All of the included studies were explorative, being classified into preliminary phase 1, which contributes to the development of new diagnostic tests. Items in regard to the availability of the representative nature of the spectrum of patients were not applicable for all studies. Detailed items for all included studies are summarized in S.Table 15.

In total, 635 differential metabolites of CRC were extracted from 62 eligible studies (S.Table 5). There were 49 metabolites shared across the 635 metabolites and the 141 metabolites with AUC>0.65.

S.Figure 10 shows the reported frequencies of the 49 metabolites in external studies. Meanwhile, there were 29 replicated metabolites between the 62 involved in the six biological pathways and the 635 metabolites reported in external studies (S.Figure 11).

As shown in S.Table 6, pathway enrichment analysis was conducted in 14 of these 62 included studies, and a total of 96 pathways were suggested to be altered in CRC. Five pathways enriched in our study had also been reported previously, with the only exception being linoleic acid metabolism. Additionally, 23 pathways were significantly enriched based on all 635 differential metabolites from the 62 included studies (S.Table 7); notably, Linoleic acid metabolism was significantly enriched.

2.3 Plasma biomarkers screening for CRC diagnosis

Firstly, S.Table 8 shows the diagnostic efficiency of all 147 metabolites. Among them, 11 metabolites were selected owing to manifesting the best AUC values (>0.85) in both the first and second stages (S.Table 9). Upon introducing these 11 metabolites into binary logistic regression, four [Eicosenoic acid, Alpha-N-phenylacetyl-L-glutamine, 3a,7a-Dihydroxycholanoic acid, and LysoPC(16:1(9Z))] showed statistically significant contribution to identifying CRC cases (S.Table 10). As expected, the combined AUCs (AUC: 1.000; 95% CI: 1.000–1.000 in the first stage; AUC: 0.989; 95% CI: 0.980–0.999 in the second stage) were higher than the AUC of any individual marker (S.Figure 12).

Secondly, there were 59 metabolites shared across the 141 metabolites with AUC>0.65 and the 62 metabolites involved in the six biological pathways. As shown in Fig. 2A-B, 2-aminobenzoic acid, 13-OxoODE, Citric acid, 2’-deoxyinosine triphosphate, Taurocholic acid, and LysoPC(16:1(9Z)) showed the highest AUCs in each pathway among the first or second stages. Combined AUCs of 0.982 (95% CI: 0.962–1.000) in the first stage and 0.974 (95% CI: 0.956–0.992) in the second stage were obtained for these six metabolites.

Thirdly, S.Table 11 shows the diagnostic efficiency of the 93 metabolites in the tissue metabolomics study. Among the 59 plasma metabolites with AUC>0.65 and were involved in six biological pathways, 13 were also confirmed to be differential metabolites in the paired tissue metabolomics study (Dataset A; Fig. 1, Fig. 2, S.Table 12).

Five of these 13 metabolites (Dataset A), L-Tryptophan, 13-OxoODE, IDP, Glycochenodeoxycholate, and LysoPC(16:0) showed the highest AUCs in following pathways: Phenylalanine, tyrosine and tryptophan biosynthesis; Linoleic acid metabolism; Purine metabolism;

Primary bile acid biosynthesis; and Glycerophospholipid metabolism (with one pathway has no replicated metabolite in plasma and tissue), and were selected as CRC panel 1(Fig. 2C). The combined AUCs and 95%CIs achieved 0.982, 0.963–1.000 in the first stage and 0.963, 0.943–0.984 in the second stage in plasma, and achieved 0.958, 0.924–0.991 in paired tissue analysis. As shown in Fig. 3, 27 metabolites (Dataset B) with AUC>0.65 in our study were also involved in the six pathways, and all of these metabolites had been reported in previous studies. Six metabolites, including L−Phenylalanine (reported frequency=20), Linoleic acid (8), Citric acid (9), Inosine (4), Glycocholic acid (3), and LysoPC(14:0) (3), were selected due to having the highest reported frequency and better AUC value in each pathway. The combined AUCs and 95%CIs achieved 0.971, 0.949–0.994 and 0.948, 0.921–0.976 in the first and second stages, respectively (CRC panel 2). Finally, the nine metabolites common to Datasets A (13 metabolites) and B (27 metabolites) were grouped as Dataset C (S.Table 12). Four metabolites, including L-Tryptophan, Linoleic acid, Glycocholic acid, and LysoPC(16:0), were selected from Dataset C, as they had multiple advantages of the highest reported frequency in multiple populations, tumor tissue reproducibility, biological significance, and favorable AUC values (CRC Panel 3). The combined AUCs and 95%CIs achieved 0.942, 0.901-0.983 and 0.937, 0.906-0.968 in the first and second stage studies in plasma, and 0.966, 0.938–0.995 in paired tissue analysis (S.Figure 14).

In addition, according to the TNM stage of tumor, the combined AUCs were all above 0.93 in the diagnosis of I/II stage patients (S.Figure 15 and S.Table 13). The AUCs of Panel 1 achieved 0.989 (95%CI, 0.976-1.000) and 0.960 (0.935-0.985) in the first and second stage studies in Chinese population. When the Youden indexes were at maximum, the sensitivity and specificity was 0.941, 0.972 in the first stage study and 0.858, 0.926 in the second stage study.

2.4 Biomarkers Validation in Targeted Metabolomics

Ultimately, we targeted and quantified 5 metabolites (L-Tryptophon, Linoleic Acid, LysoPC(14:0), LysoPC(16:0), LysoPC(18:0)) with higher peak intensity belonging to 27 metabolites (Dataset B) using UPLC/Q-TOF-MS/MS in plasma. Totally, a batch of 504 plasma samples, including 251 plasma samples from CRC patients, 253 normal controls from healthy check-up crowd were detected.

The plasma L-Tryptophon, Linoleic Acid, LysoPC(14:0), LysoPC(18:0) levels remain significantly lower or higher in the CRC samples than in the normal control , but the concentration of LysoPC(16:0) was not significantly different among the groups (Fig. 4A). The combined AUCs and

95%CIs achieved 0.728, 0.681-0.775 in plasma (Fig. 4B). Detailed information of sensitivity and specificity of all prediction models was shown in S.Table 19.

III Supplementary Figures

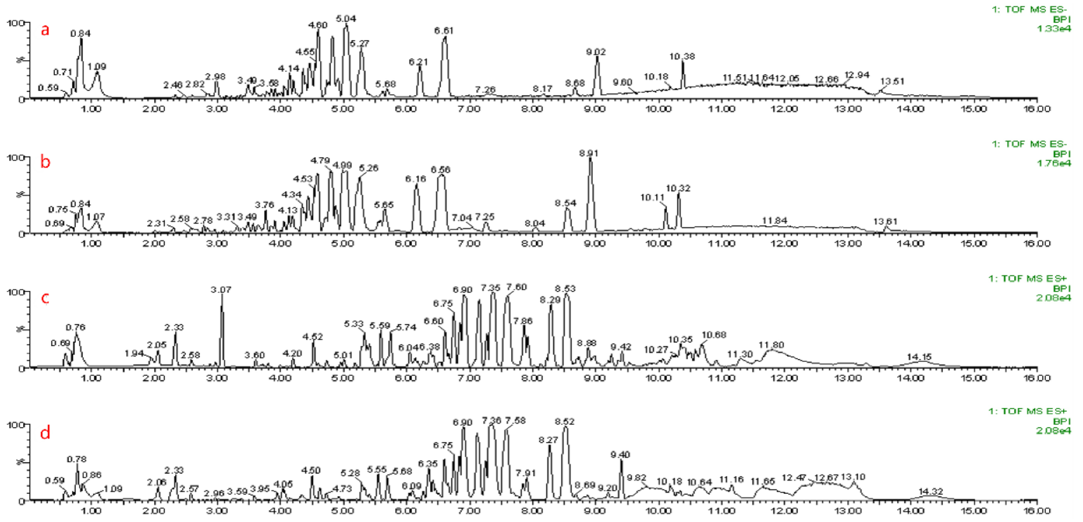

S.Figure 1. Representative ultra-performance liquid chromatography and quadrupole time-of-flight tandem mass spectrometry (UPLC/Q-TOF MS/MS)-based peak intensity chromatogram of human plasma. (a) Colorectal cancer patients in ESI$^-$ mode; (b) Healthy individuals in ESI$^-$ mode; (c) Colorectal cancer patients in ESI$^+$ mode; (d) Healthy individuals in ESI$^+$ mode.

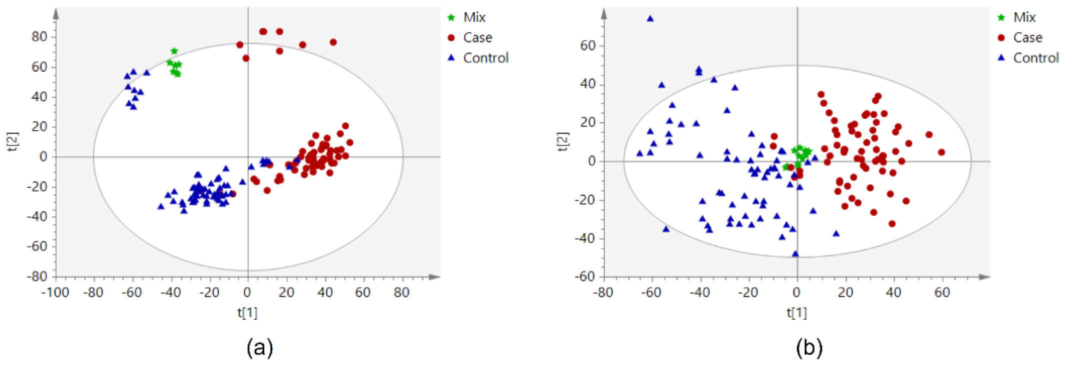

S.Figure 2. Two-dimensional PCA score plots of plasma from colorectal cancer patients, healthy controls, and QC samples. (a) Plasma samples from CRC patients (red circle), healthy individuals (blue triangle) and QC samples (green star) in ESI$^-$ mode. (b) Plasma samples from CRC patients (red circle), healthy individuals (blue triangle) and QC samples (green star) in ESI$^+$ mode. t[1], component 1; t[2], component 2.

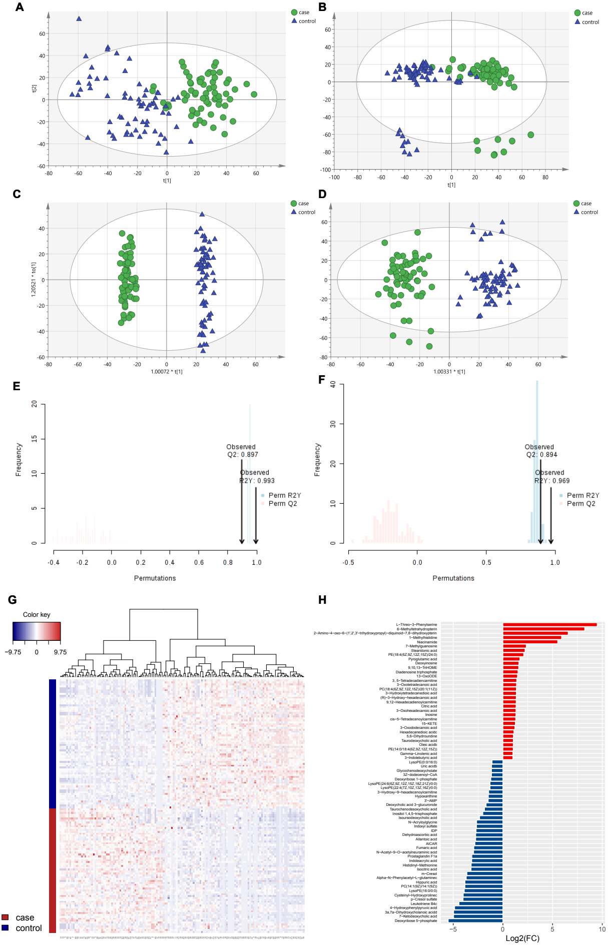
A
B
C
D
E
F
G
H
case
control
t[1]
t[2]
1.00072 * t[1]
1.20521 * to[1]
1.00331 * t[1]
Frequency
Observed
Q2: 0.897
Observed
R2Y: 0.993
Observed
Q2: 0.894
Observed
R2Y: 0.969
Perm R2Y
Perm Q2
Permutations
Color key
-9.75
0
9.75
Log2(FC)

S.Figure 3-1. Global plasma metabolic profiling analysis by UPLC/Q-TOF-MS/MS of the first stage (A) PCA score plot of the first stage in ESI^+ mode, $R^2X=0.154$, $Q^2=0.107$. (B) PCA score plot of the first stage in ESI^- mode, $R^2X=0.206$, $Q^2=0.168$. (C) OPLS-DA score plot of the first stage in ESI^+ mode, $R^2X=0.194$, $R^2Y=0.988$, $Q^2=0.896$. (D) OPLS-DA score plot of the first stage in ESI^- mode, $R^2X=0.155$, $R^2Y=0.925$, $Q^2=0.871$. (E) and (F) are permutation test results of OPLS-DA model. R^2Y value represents the goodness of fit of the model. Q^2 value represents the predictability of the model. (E) Result of the first stage in ESI^+ mode. (F) Result of the first stage in ESI^- mode. (G) Heatmap visualization constructed based on the 147 identified metabolites. Rows: samples; columns: differential metabolites (the ID numbers of metabolites are same as in S.Table 2). Color key indicates metabolite expression: dark blue, lowest; dark red, highest. Compared with healthy controls, 65 metabolites were upregulated in the plasma of CRC patients, whereas 82 were downregulated. (H) The log2 fold-change of 70 metabolites with a threshold of 2.

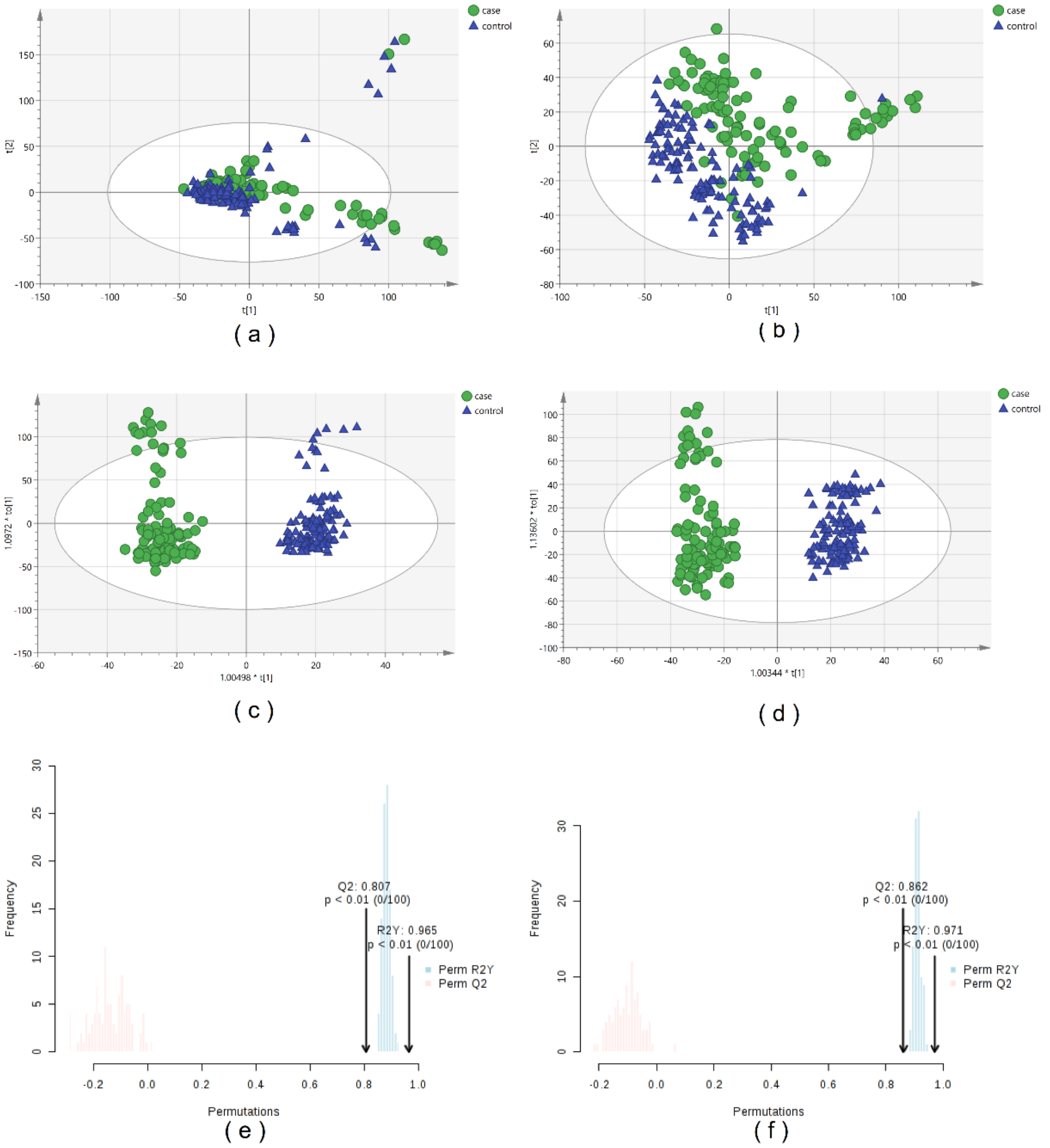

S.Figure 3-2. PCA and OPLS-DA score plots of the second stage and permutation test results of OPLS-DA model. (a) PCA score plot of the second stage in ESI$^+$ mode, R^2X=0.161, Q^2=0.111. (b) PCA score plot of the second stage in ESI$^-$ mode, R^2X=0.15, Q^2=0.123. (c) OPLS-DA score plot of the second stage in ESI$^+$ mode, R^2X=0.219, R^2Y=0.962, Q^2=0.83. (d) OPLS-DA score plot of the second stage in ESI$^-$ mode, R^2X=0.199, R^2Y=0.955, Q^2=0.868. R^2Y value represents the goodness of fit of the model. Q^2 value represents the predictability of the model. (e) Permutation test result of the second stage in ESI$^+$ mode; (f) Permutation test result of the second stage in ESI$^-$ mode.

A

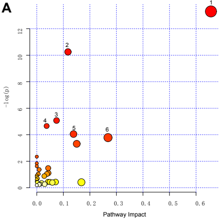

B

Pathway enrichment analysis for differential metabolites in this two-stage case-control study

NO.	Pathway Name	Total	Hits	P-value	FDR	Impact
1	Linoleic acid metabolism	15	6	1.67E-06	0.000133	0.65625
2	Purine metabolism	92	11	3.54E-05	0.001414	0.11747
3	Phenylalanine, tyrosine and tryptophan biosynthesis	27	4	0.006263	0.16702	0.07442
4	Primary bile acid biosynthesis	47	5	0.009488	0.18976	0.03703
5	Citrate cycle (TCA cycle)	20	3	0.017427	0.27883	0.13837
6	Glycerophospholipid metabolism	39	4	0.022778	0.3037	0.26835

C

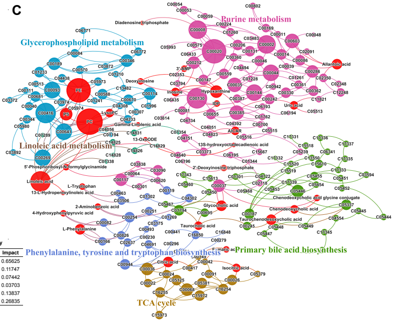

S.Figure 4. The six significantly enriched pathways by differential plasma metabolites in this two-stage case-control study. (A) & (B) showing the pathway enrichment results for differential plasma metabolites. (C) An overview of the six significantly enriched pathways. Red painted circles indicate the metabolites identified from our two-stage case-control study.

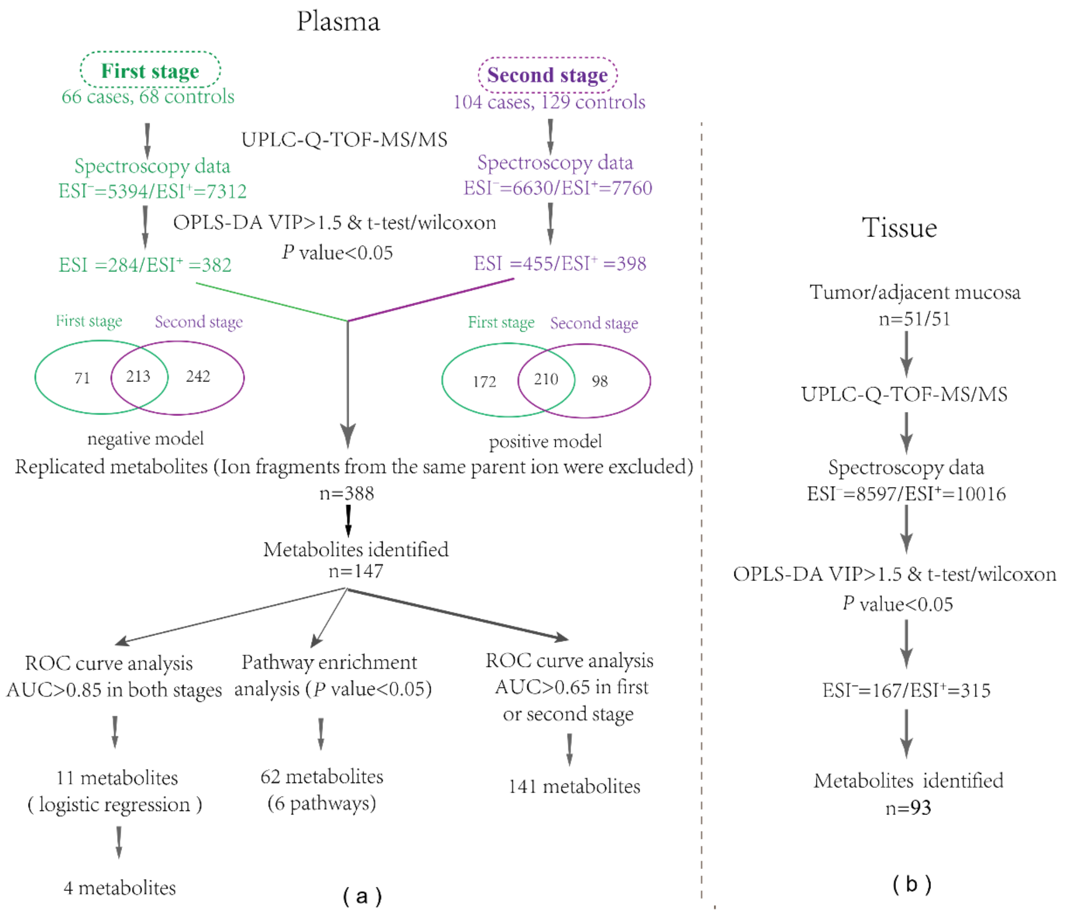

S.Figure 5. Data processing, statistical analysis, and identification of differential metabolites in plasma and tissue by UPLC/Q-TOF-MS/MS

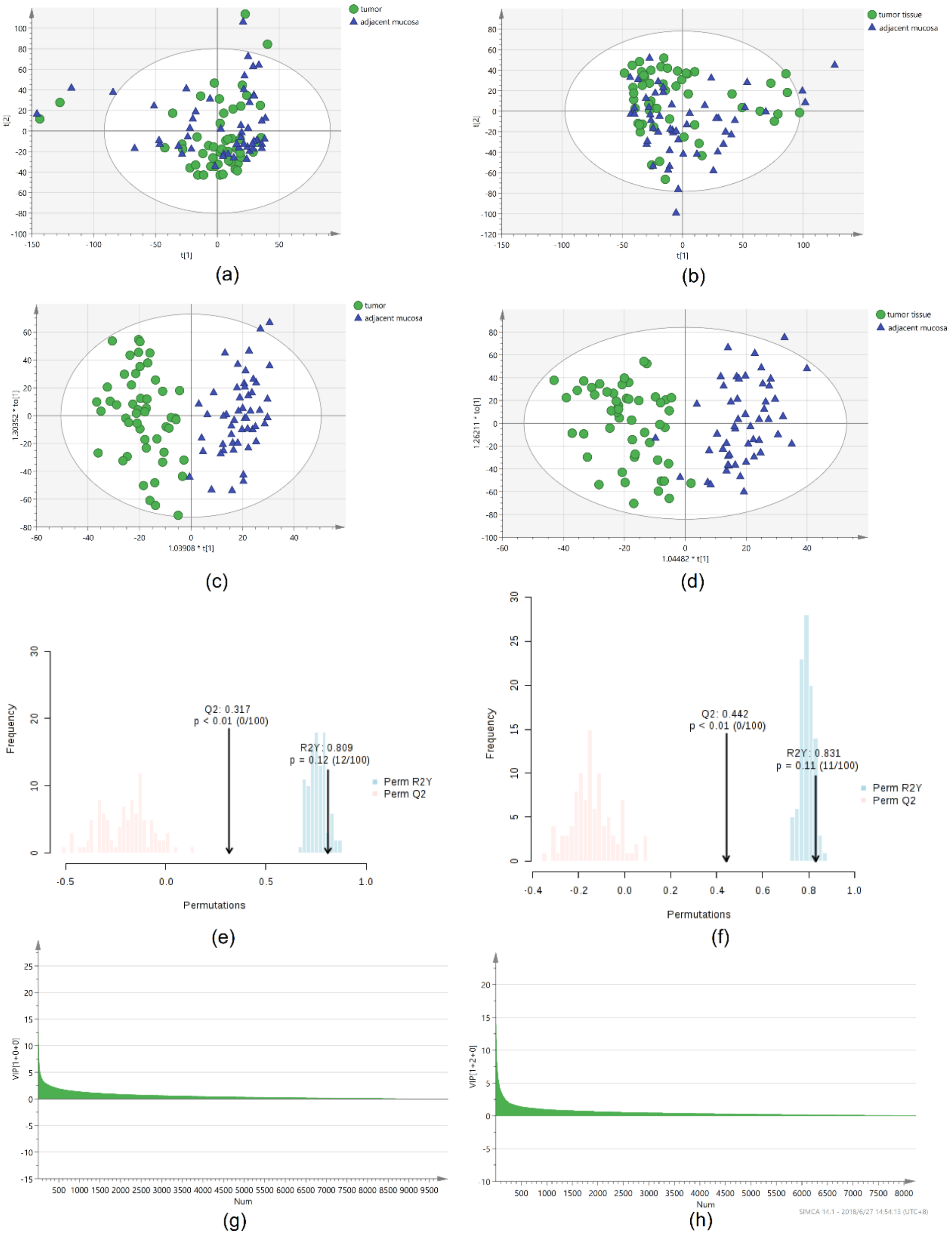

S.Figure 6. PCA and OPLS-DA score plots of tissue from colorectal cancer patients and permutation test results of OPLS-DA model. (a) PCA score plot in ESI^+ model, R^2X=0.531, Q^2=0.215. (b) PCA score plot in ESI^- mode, R^2X=0.461, Q^2=0.247; (c) OPLS-DA score plot in ESI^+ model, R^2X=0.054, R^2Y=0.548, Q^2=0.281. (d) OPLS-DA score plot in ESI^- model, R^2X=0.244, R^2Y=0.794, Q^2=0.406. R^2Y value represents the goodness of fit of the model. Q^2 value represents the

predictability of the model. (e) Permutation test result of the OPLS-DA model in ESI$^+$ model. R^2Y=0.901 and Q^2=0.348; (f) Permutation test result of the OPLS-DA model in ESI$^-$ model. R^2Y=0.831 and Q^2=0.442. (g) VIP value of metabolites in ESI$^+$ model; (h) VIP value of metabolites in ESI$^-$ model.

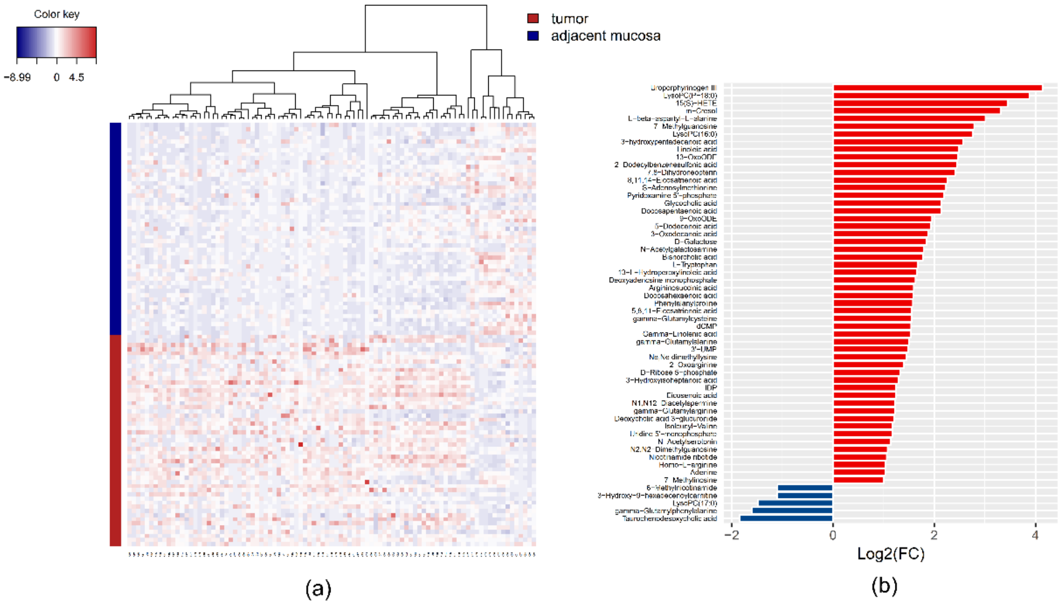

S.Figure 7. General tendency of metabolites discriminated in paired tissue metabolomics analysis. (a) Heatmap visualization constructed based on the 93 identified metabolites. Rows: samples; columns: differential metabolites. Red: tumor tissue; blue: adjacent mucosa. Color key indicates metabolite expression: dark blue: lowest; dark red: highest. Compared with adjacent mucosa, 80 metabolites were upregulated in tumor tissue of CRC patients, whereas 13 were downregulated. (b) Fold-change of 57 metabolites with a threshold of 2.

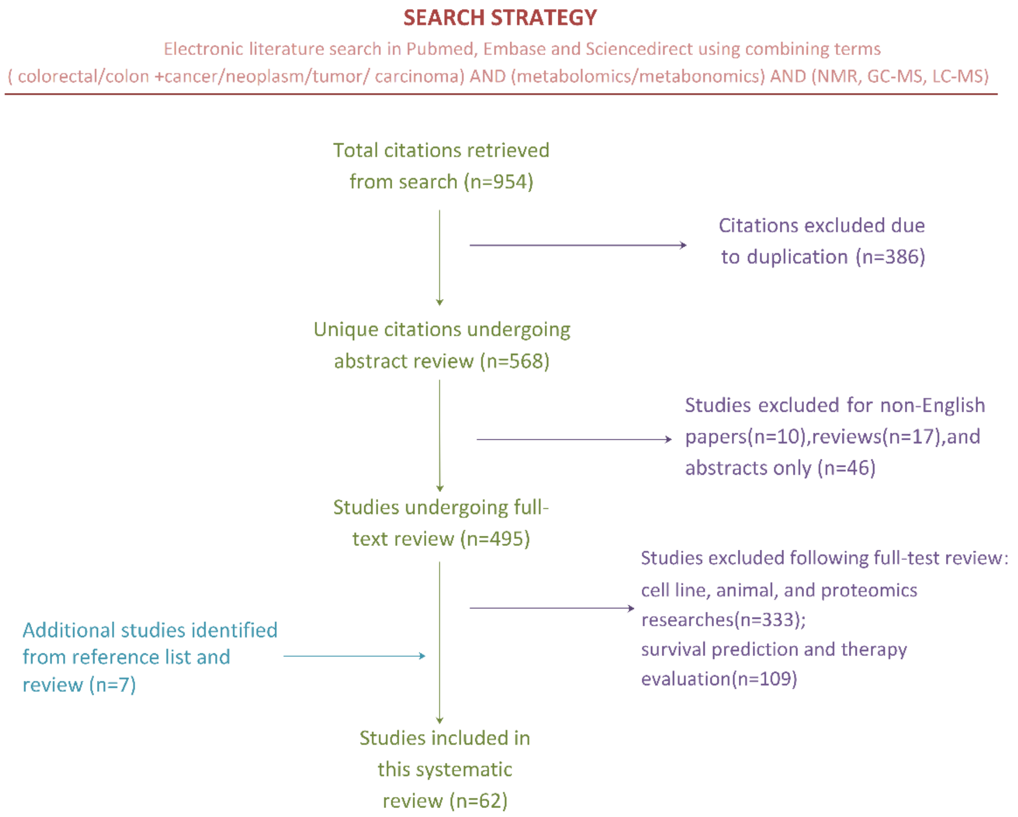

S.Figure 8. Workflow of literature retrieve in the systematic review of metabolomics studies of colorectal cancer.

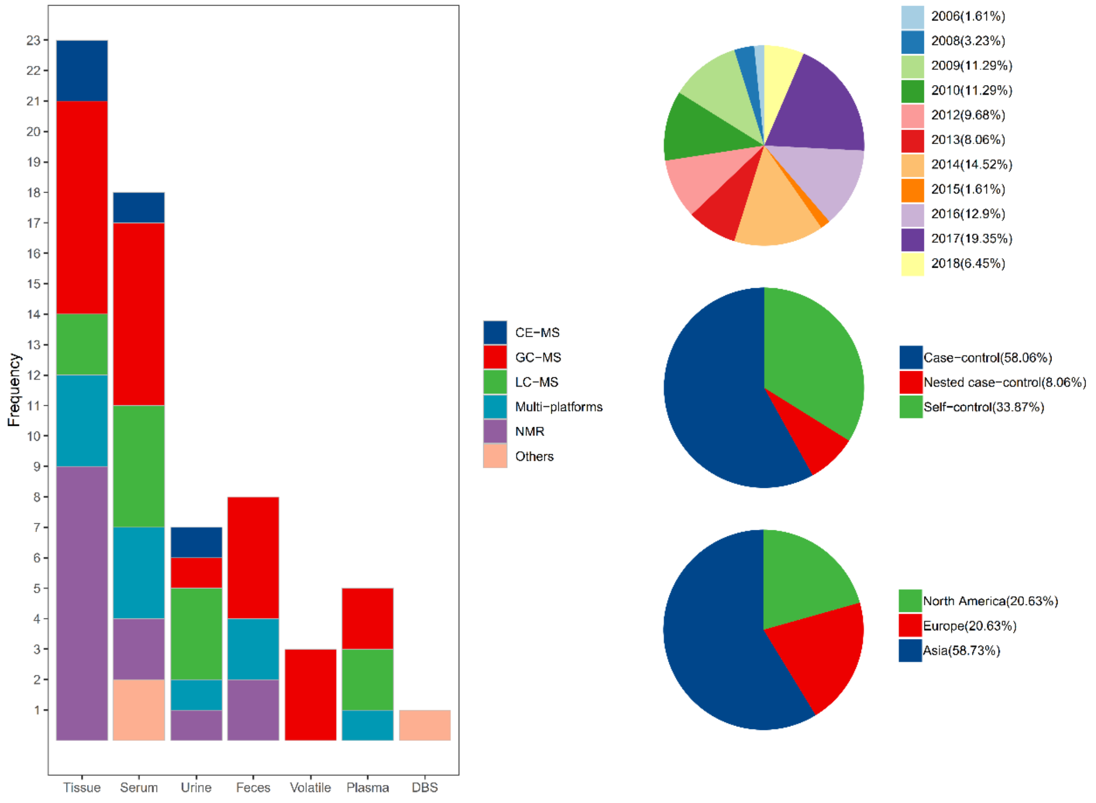

S.Figure 9. Overall distribution of sample types, platforms, publication year, study design, and populations in the systematic review of metabolomics studies of colorectal cancer.

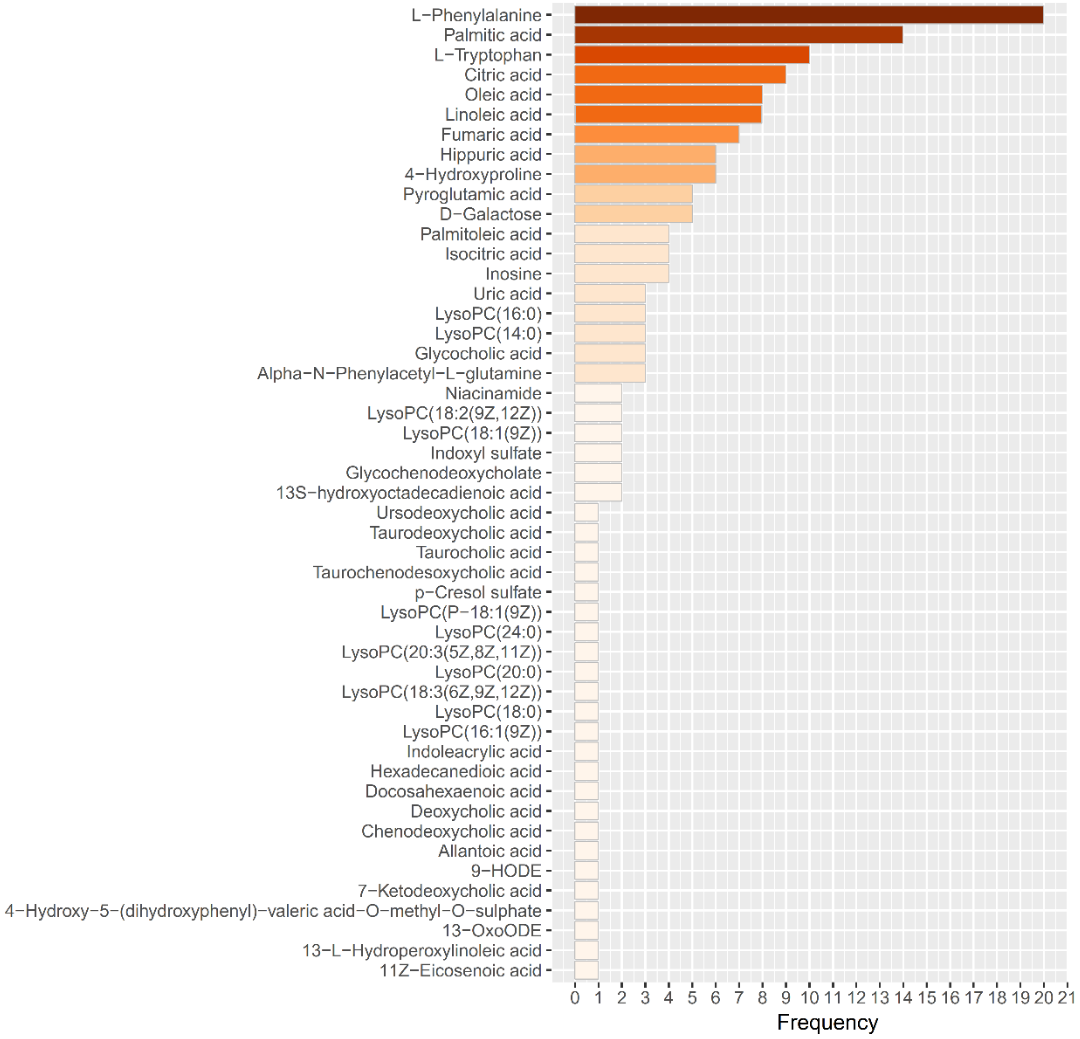

S.Figure 10. Reported frequency in previous studies of the differential metabolites with good diagnostic value in our study

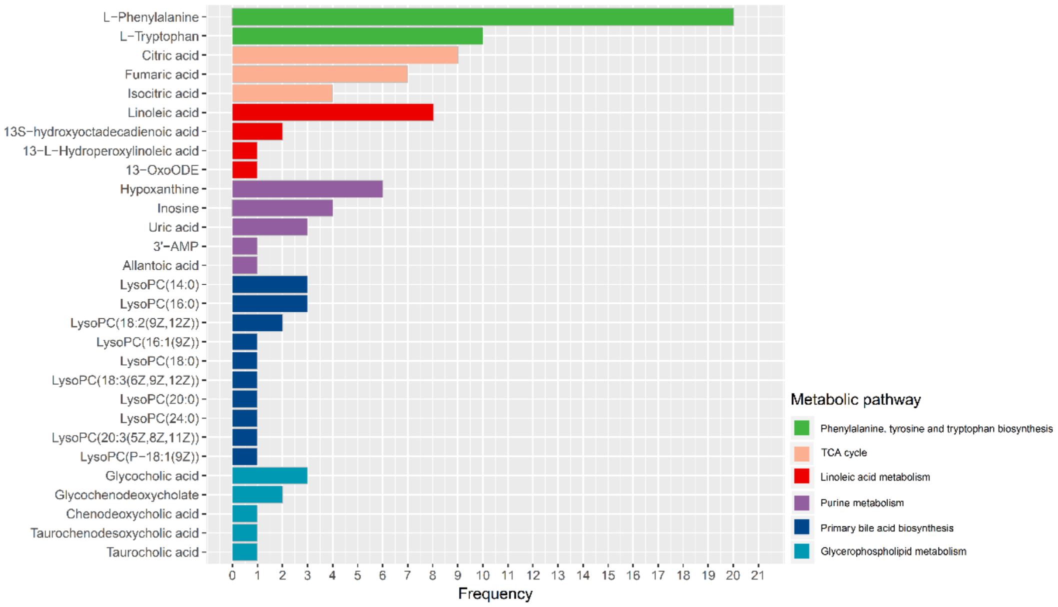

S.Figure 11. Reported frequency in previous studies of the differential metabolites with biological significance in our study

Diagnostic efficiency of the four plasma metabolites in this two-stage case-control study

Metabolites	First stage		Second stage	
	AUC (95% CI)	P-value	AUC (95% CI)	P-value
Eicosenoic acid	1.000 (1.000-1.000)	<0.0001	0.868 (0.820-0.917)	<0.0001
Alpha-N-Phenylacetyl-L-glutamine	0.936 (0.887-0.985)	<0.0001	0.928 (0.885-0.961)	<0.0001
3a,7a-Dihydroxycholanoic acid	0.913 (0.855-0.970)	<0.0001	0.923 (0.904-0.970)	<0.0001
LysoPC(16:1(9Z))	0.969 (0.944-0.994)	<0.0001	0.937 (0.904-0.970)	<0.0001
combined	1.000 (1.000-1.000)	<0.0001	0.989 (0.980-0.999)	<0.0001

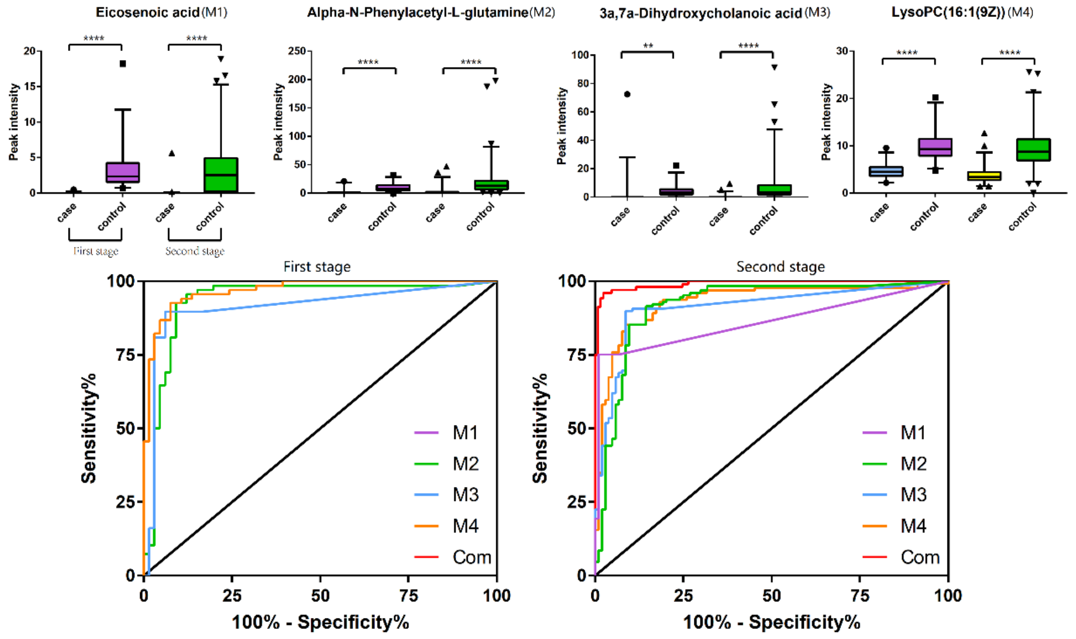

S.Figure 12. Four metabolic biomarkers with the best diagnostic value of colorectal cancer in Northeastern Chinese population. Box plots showing ion intensities of the four metabolites in CRC patients and healthy controls in the first and second stages. **: *P*<0.01; ****: *P*<0.0001. AUCs of the four metabolites [Eicosenoic acid, Alpha-N-phenylacetyl-L-glutamine, 3a,7a-Dihydroxycholanoic acid, lysoPC(16:1(9Z))] and a multi-marker panel (Red lines show the combined AUCs in the first and second atages).

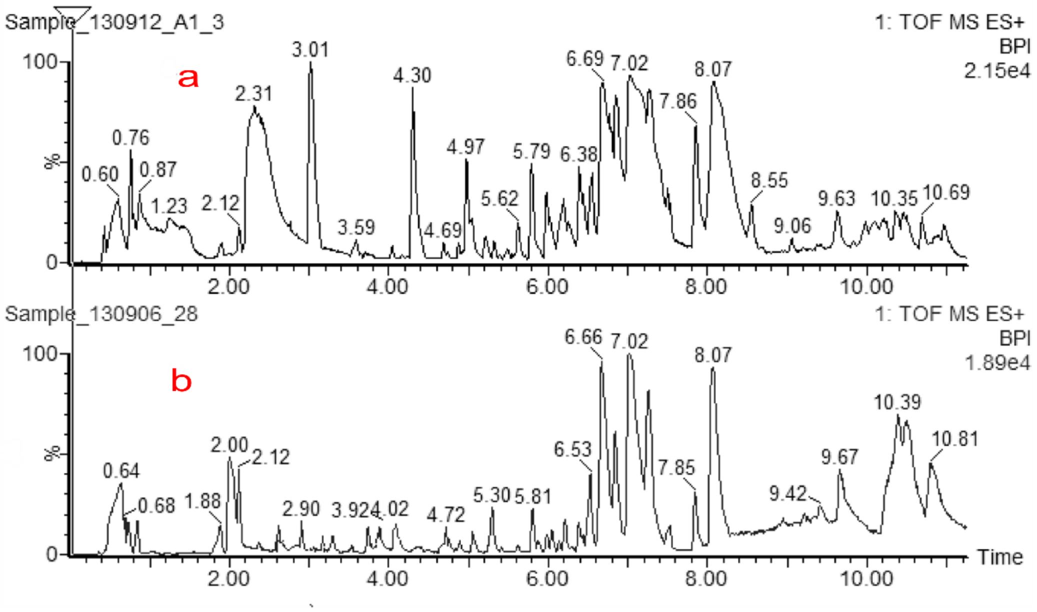

S.Figure 13. Comparison of based peak intensity chromatograms between our and previous reported methods of preparing human plasma. (a) Optimized preparation method with the recycle of polar metabolites in this study; (b) General preparation method in previous studies.

Diagnostic efficiency of the four metabolites (Panel 3) in plasma and tissue

Metabolites	First stage		Second stage		Paired-tissue	
	AUC (95% CI)	*P*-value	AUC (95% CI)	*P*-value	AUC (95% CI)	*P*-value
L-Tryptophan	0.764 (0.683-0.844)	<0.0001	0.768 (0.706-0.830)	<0.0001	0.735 (0.633-0.836)	<0.0001
Linoleic acid	0.794 (0.713-0.874)	<0.0001	0.688 (0.620-0.756)	<0.0001	0.751 (0.653-0.849)	<0.0001
Glycocholic acid	0.641 (0.548-0.735)	<0.0001	0.659 (0.587-0.731)	<0.0001	0.831 (0.747-0.916)	<0.0001
LysoPC(16:0)	0.927 (0.884-0.971)	<0.0001	0.891 (0.848-0.933)	<0.0001	0.828 (0.746-0.911)	<0.0001
Combined	0.942 (0.901-0.983)	<0.0001	0.937 (0.906-0.968)	<0.0001	0.966 (0.938-0.995)	<0.0001

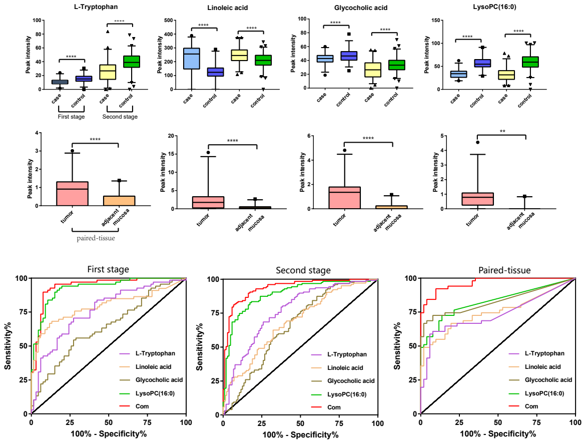

S.Figure 14. The optimal candidate metabolic biomarkers for colorectal cancer diagnosis in diverse populations. Box plots showing ion intensities of the four metabolites (Panel 3) in both the first and second stages. **, *P*<0.01; ****, *P*<0.0001. AUCs of the four metabolites [L-Tryptophan, Linoleic acid, Glycocholic acid, and LysoPC(16:0)] and a multi-marker panel in the first and second stages.

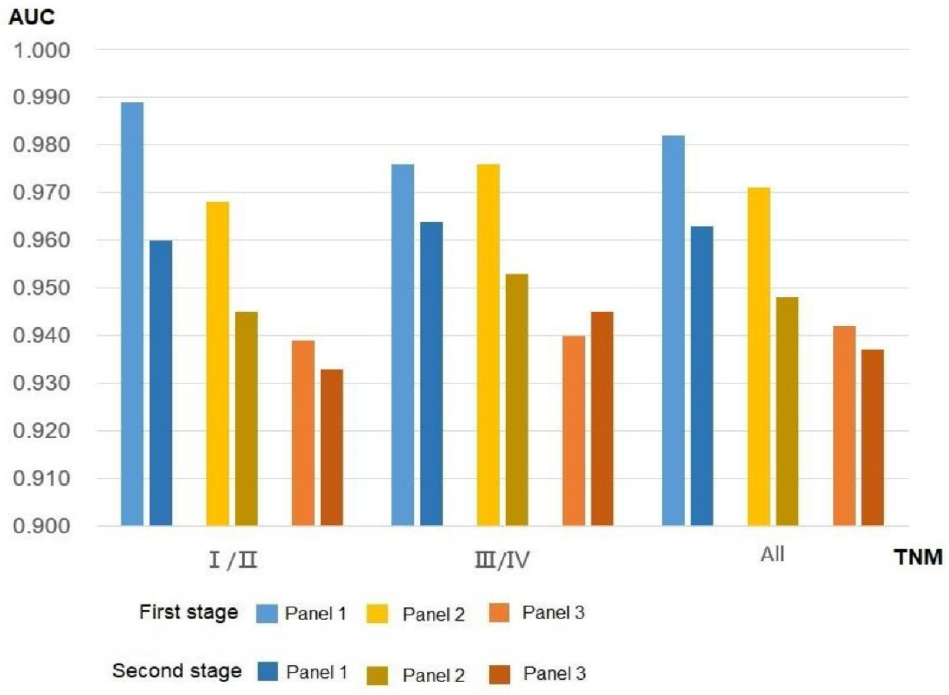

S.Figure 15. The diagnositic efficacy of panels in different TNM stages of colorectal cancer.

S.Table 1. Demographic characteristics of participants in this two-stage case-control study in a population from China

Variables	Training Set			Validation Set		
	Case[1]	Control[2]	*P*-value	Case[1]	Control[2]	*P*-value
Total	66	68		104	129	
Age (mean$\pm SD$)	59.15$\pm$11.03	58.98$\pm$10.84	0.930	59.84$\pm$10.56	58.95$\pm$11.06	0.549
Sex (F/M)	31/34	31/33	0.933	39/59	50/72	0.877
BMI (Kg/m^2)	23.26$\pm$3.80	23.93$\pm$4.03	0.332	23.81$\pm$3.36	24.17$\pm$3.34	0.437
TNM						
Stage I	5			13		
Stage II	31			45		
Stage III	23			32		
Stage IV	5			6		

Missing data: Training set: Age, 1 case, 4 controls; Sex, 1 case, 4 controls; BMI, 2 cases, 4 controls; TNM, 2 cases. Validation set: Age, 6 cases, 7controls; Sex, 6 cases, 7 controls; BMI, 7 cases, 9 controls; TNM: 8 cases.

[1]Any patient with neuroendocrine carcinoma, malignant melanoma, non-Hodgkin's lymphoma, gastrointestinal stromal tumors, and Lynch syndrome CRC was excluded. Only newly diagnosed, histopathologically confirmed cases were retained. No form of any treatment, including surgery and chemo- or radiation therapy, was given to the patients prior to the collection of blood samples. No form of any treatment, including surgery, chemotherapy or radiation therapy, was given to the patients prior to collecting of blood samples.

[2]Any subject in the control group with inflammatory conditions, gastrointestinal tract disorders, or a history of polyps, adenoma, or other cancer-related disease was excluded.

S.Table 2. Colorectal cancer metabolites identified in the two-stage case-control study in a population from China

NO.	[a]RT	Mass	HMDB number	Compounds	Adduct type	ESI mode	Delta (ppm)	Tendency (case/control)	Fold change
1	0.63	357.0287	HMDB0059977	4-Hydroxy-5-(dihydroxyphenyl)-valeric acid-O-methyl-O-sulphate[c]	M+Na-2H	ESI-	7	↑	1.75
2	0.64	737.0502	HMDB0001155	Diadenosine triphosphate[b]	M-H20-H	ESI-	18	↑	2.87
3	0.64	273.0716	HMDB0000071	Deoxyinosine[b]	M+Na-2H	ESI-	41	↑	3.05
4	0.64	313.0649	HMDB0000195	Inosine[b]	M+FA-H	ESI-	45	↑	2.48
5	0.65	472.9486	HMDB0003537	2'-Deoxyinosine triphosphate[b]	M-H20-H	ESI-	38	↑	1.93
6	0.66	220.8725	HMDB0059921	Trimetaphosphoric acid[d]	M-H20-H	ESI-	37	↓	1.94
7	0.69	203.054	HMDB0000143	D-Galactose[c]	M+Na	ESI+	7	↑	1.61
8	0.70	215.039	HMDB0028776	Cysteinyl-Hydroxyproline[c]	M-H20-H	ESI-	47	↑	15.88
9	0.70	160.0395	HMDB0001123	2-Aminobenzoic acid[b]	M+Na	ESI+	16	↓	1.38
10	0.71	335.0491	HMDB0001068	D-Sedoheptulose 7-phosphate[c]	M+FA-H	ESI-	32	↑	1.36
11	0.73	346.0567	HMDB0003540	3'-AMP[b]	M-H	ESI-	3	↓	2.79
12	0.74	167.0191	HMDB0000289	Uric acid[b]	M-H	ESI-	12	↓	2.20
13	0.76	252.9758	HMDB0001031	Deoxyribose 5-phosphate[d]	M+K	ESI+	46	↓	45.39
14	0.77	315.0745	HMDB0001308	5'-Phosphoribosyl-N-formylglycinamide[d]	M+H	ESI+	50	↑	1.85
15	0.81	247.095	HMDB0028754	Aspartyl-Hydroxyproline[d]	M+H	ESI+	35	↑	1.89
16	0.81	160.059	HMDB0029433	L-2-Amino-4-methylenepentanedioic acid[d]	M+H	ESI+	9	↑	1.90
17	0.82	132.0707	HMDB0000725	4-Hydroxyproline[b]	M+H	ESI+	39	↑	1.60
18	0.82	191.0172	HMDB0000094	Citric acid[b]	/	ESI-&ESI+	13	↓	2.51
19	0.82	137.045	HMDB0000157	Hypoxanthine[b]	M+H	ESI+	6	↑	2.75
20	0.84	128.0338	HMDB0000267	Pyroglutamic acid[b]	M-H	ESI-	12	↑	3.27
21	0.84	374.1111	HMDB0000794	N-Acetyl-9-O-acetylneuraminic acid[d]	M+H	ESI+	14	↓	7.72
22	0.86	429.0258	HMDB0003335	IDP[b]	M+H	ESI+	12	↓	6.49
23	0.87	337.0643	HMDB0001107	7-Methylguanosine[d]	M+K	ESI+	12	↑	5.10
24	0.92	247.0948	HMDB0000497	5,6-Dihydrouridine[b]	/	ESI-&ESI+	9	↑	2.08
25	1.07	215.0175	HMDB0001209	Allantoic acid[b]	M+K	ESI+	1	↓	6.68
26	1.08	210.0626	HMDB0000734	Indoleacrylic acid[b]	M+Na	ESI+	48	↓	8.81

NO.	[a]RT	Mass	HMDB number	Compounds	Adduct type	ESI mode	Delta (ppm)	Tendency (case/control)	Fold change
27	1.09	383.0474	HMDB0001517	AICAR[b]	M+FA-H	ESI-	35	↓	7.03
28	1.09	191.0176	HMDB0000193	Isocitric acid[b]	/	ESI-&ESI+	11	↓	9.22
29	1.09	139.0029	HMDB0000134	Fumaric acid[b]	M+Na	ESI+	20	↓	7.62
30	1.09	175.0257	HMDB0001264	Dehydroascorbic acid[b]	M+H	ESI+	11	↓	6.66
31	1.09	420.9737	HMDB0001498	Inositol 1,4,5-trisphosphate[d]	M+H	ESI+	10	↓	4.06
32	2.02	166.0876	HMDB0000159	L-Phenylalanine[b]	M+H	ESI+	8	↓	1.51
33	2.06	123.0544	HMDB0001406	Niacinamide[b]	M+H	ESI+	7	↑	45.78
34	2.30	203.0817	HMDB0000929	L-Tryptophan[b]	/	ESI-&ESI+	4	↓	1.44
35	2.52	220.0507	HMDB0002249	6-Methyltetrahydropterin[b]	M+H	ESI+	40	↑	301.24
36	2.60	263.1038	HMDB0006344	Alpha-N-Phenylacetyl-L-glutamine[c]	/	ESI-&ESI+	2	↓	12.91
37	2.60	212.0025	HMDB0000682	Indoxyl sulfate[b]	M-H	ESI-	1	↓	6.29
38	2.62	178.051	HMDB0000714	Hippuric acid[b]	M-H	ESI-	0	↓	14.08
39	2.64	130.0503	HMDB0001843	N-Acryloylglycine[d]	M+H	ESI+	3	↓	6.25
40	2.64	287.1052	HMDB0028891	Histidinyl-Methionine[d]	M+H	ESI+	42	↓	9.12
41	2.87	107.0473	HMDB0002048	m-Cresol[b]	M-H	ESI-	27	↓	12.33
42	2.88	187.0021	HMDB0011635	p-Cresol sulfate[b]	M-H	ESI-	26	↓	16.10
43	3.00	696.4384	HMDB0007900	PC(14:1(9Z)/14:1(9Z))[d]	M+Na	ESI+	27	↓	14.16
44	3.05	170.094	HMDB0000001	1-Methylhistidine[b]	M+H	ESI+	9	↑	60.14
45	3.05	220.0443	HMDB0002184	L-Threo-3-Phenylserine[c]	M+K	ESI+	33	↑	720.13
46	3.14	204.1019	HMDB0002096	3-Indolebutyric acid[b]	M+H	ESI+	0	↑	2.01
47	3.17	290.1157	HMDB0012144	2-Amino-4-oxo-6-(1',2',3'-trihydroxypropyl)-diquinoid-7,8-dihydroxypterin[d]	M+H	ESI+	21	↑	94.94
48	3.18	201.0239	HMDB0000707	4-Hydroxyphenylpyruvic acid[b]	M+Na-2H	ESI-	35	↓	28.99
49	3.31	213.0241	HMDB0001351	Deoxyribose 1-phosphate[d]	M-H	ESI-	34	↓	2.32
50	3.39	567.3157	HMDB0002596	Deoxycholic acid 3-glucuronide[d]	M-H	ESI-	3	↓	3.22
51	3.48	329.2322	HMDB0004710	9,10,13-TriHOME[c]	M-H	ESI-	3	↑	3.01
52	3.49	448.307	HMDB0000637	Glycochenodeoxycholate[b]	M-H	ESI-	0	↓	2.21
53	3.72	568.3004	HMDB0061698	1-Stearoylglycerophosphoserine[d]	M+FA-H	ESI-	20	↑	1.88

NO .	[a]RT	Mass	HMDB number	Compounds	Adduct type	ESI mode	Delta (ppm)	Tendency (case/control)	Fold change
54	3.73	255.1577	HMDB0000394	3-Hydroxytetradecanedioic acid[d]	M-H20-H	ESI-	8	↑	2.55
55	3.77	448.2995	HMDB0000631	Deoxycholic acid glycine conjugate[c]	M-H	ESI-	16	↓	1.36
56	3.79	544.3038	HMDB0000951	Taurochenodesoxycholic acid[b]	M+FA-H	ESI-	16	↑	3.61
57	3.79	391.2793	HMDB0000626	Deoxycholic acid[c]	M-H	ESI-	16	↓	1.44
58	3.92	391.2809	HMDB0000946	Ursodeoxycholic acid[b]	M-H	ESI-	11	↓	1.55
59	3.92	335.2223	HMDB0001085	Leukotriene B4[c]	M-H	ESI-	1	↑	21.36
60	3.96	213.1486	HMDB0010727	3-Oxododecanoic acid[b]	M-H	ESI-	5	↑	2.31
61	4.04	948.2733	HMDB0004257	3Z-dodecenoyl-CoA[d]	M+H	ESI+	1	↓	2.22
62	4.09	452.2815	HMDB0011503	LysoPE(16:0/0:0)[d]	M-H	ESI-	7	↓	14.78
63	4.18	526.2928	HMDB0011495	LysoPE(0:0/22:5(7Z,10Z,13Z,16Z,19Z))[d]	M-H	ESI-	2	↓	1.54
64	4.19	552.289	HMDB0011499	LysoPE(0:0/24:6(6Z,9Z,12Z,15Z,18Z,21Z)) [d]	M-H	ESI-	37	↓	1.55
65	4.20	452.2743	HMDB0011473	LysoPE(0:0/16:0)[d]	M-H	ESI-	9	↓	2.12
66	4.21	391.2832	HMDB0000686	Isoursodeoxycholic acid[d]	M-H	ESI-	6	↓	5.08
67	4.25	285.2068	HMDB0000672	Hexadecanedioic acid[c]	M-H	ESI-	1	↑	2.15
68	4.31	391.285	HMDB0000384	3a,7a-Dihydroxycholanoic acid[d]	M-H	ESI-	1	↓	31.07
69	4.31	427.2633	HMDB0000391	7-Ketodeoxycholic acid[b]	M+Na-2H	ESI-	39	↓	31.12
70	4.41	478.2925	HMDB0011506	LysoPE(18:1(9Z)/0:0)[b]	M-H	ESI-	3	↓	1.69
71	4.43	528.3045	HMDB0011523	LysoPE(22:4(7Z,10Z,13Z,16Z)/0:0)[d]	M-H	ESI-	10	↓	2.45
72	4.50	271.228	HMDB0010734	(R)-3-Hydroxy-hexadecanoic acid[d]	M-H	ESI-	0	↑	2.54
73	4.53	466.2939	HMDB0010379	LysoPC(14:0)[c]	/	ESI-&ESI+	0	↓	1.72
74	4.53	516.2872	HMDB0010387	LysoPC(18:3(6Z,9Z,12Z))[c]	/	ESI-&ESI+	43	↓	1.59
75	4.53	526.3146	HMDB0011494	LysoPE(0:0/22:5(4Z,7Z,10Z,13Z,16Z)) [d]	M-H	ESI-	39	↓	1.71
76	4.55	528.3005	HMDB0011493	LysoPE(0:0/22:4(7Z,10Z,13Z,16Z))[d]	M-H	ESI-	17	↓	1.58
77	4.57	311.2232	HMDB0003871	13-L-Hydroperoxylinoleic acid[c]	M-H	ESI-	1	↑	1.84
78	4.63	241.1817	HMDB0010730	3-Oxotetradecanoic acid[d]	M-H	ESI-	3	↑	2.58
79	4.64	414.3018	HMDB0013333	3-Hydroxy-9-hexadecenoylcarnitine[d]	M+H	ESI+	47	↓	2.69
80	4.72	295.2242	HMDB0004667	13S-hydroxyoctadecadienoic acid[c]	M-H	ESI-	12	↑	1.79
81	4.73	552.3327	HMDB0011529	LysoPE(24:6(6Z,9Z,12Z,15Z,18Z,21Z)/0:0)[d]	M-H	ESI-	42	↓	2.45

NO .	[a]RT	Mass	HMDB number	Compounds	Adduct type	ESI mode	Delta (ppm)	Tendency (case/control)	Fold change
82	4.75	277.2175	HMDB0010223	9-HODE[d]	M-H	ESI-	3	↑	1.73
83	4.81	540.3244	HMDB0010397	LysoPC(20:5(5Z,8Z,11Z,14Z,17Z))[d]	/	ESI-&ESI+	27	↓	1.46
84	4.92	293.2115	HMDB0004668	13-OxoODE[c]	/	ESI-&ESI+	2	↑	2.77
85	5.23	478.2939	HMDB0011475	LysoPE(0:0/18:1(11Z))[c]	M-H	ESI-	0	↓	1.32
86	5.28	566.3409	HMDB0010404	LysoPC(22:6(4Z,7Z,10Z,13Z,16Z,19Z))[c]	/	ESI-&ESI+	28	↓	1.20
87	5.31	500.3153	HMDB0000896	Taurodeoxycholic acid[b]	M+H	ESI+	23	↑	2.06
88	5.32	464.3125	HMDB0000138	Glycocholic acid[c]	/	ESI-&ESI+	23	↑	1.51
89	5.34	466.3259	HMDB0006319	Alpha-linolenyl carnitine[c]	M+FA-H	ESI-	18	↓	1.60
90	5.38	518.3218	HMDB0010388	LysoPC(18:3(9Z,12Z,15Z))[c]	M+H	ESI+	4	↓	1.68
91	5.63	368.2799	HMDB0013331	3, 5-Tetradecadiencarnitine[d]	M+H	ESI+	1	↑	2.62
92	5.69	494.3224	HMDB0010382	LysoPC(16:0)[c]	/	ESI-&ESI+	6	↓	1.69
93	5.69	544.3186	HMDB0010393	LysoPC(20:3(5Z,8Z,11Z))d	/	ESI-&ESI+	41	↓	1.58
94	5.69	554.347	HMDB0011522	LysoPE(22:2(13Z,16Z)/0:0)[d]	M+Na-2H	ESI-	44	↓	1.77
95	5.81	317.2129	HMDB0010210	15-KETE[d]	M-H	ESI-	2	↑	2.39
96	5.93	520.3427	HMDB0002815	LysoPC(18:1(9Z))[d]	M-H	ESI-	4	↓	1.93
97	6.07	370.2969	HMDB0002014	cis-5-Tetradecenoylcarnitine[d]	M+H	ESI+	5	↑	2.44
98	6.11	664.4146	HMDB0008832	PE(14:0/18:4(6Z,9Z,12Z,15Z))[d]	M-H20-H	ESI-	30	↑	2.05
99	6.20	568.3572	HMDB0010403	LysoPC(22:5(7Z,10Z,13Z,16Z,19Z))[d]	M-H	ESI-	29	↓	1.27
100	6.20	558.3323	HMDB0011520	LysoPE(22:0/0:0)[d]	M+Na-2H	ESI-	39	↓	1.40
101	6.26	396.3135	HMDB0013334	9,12-Hexadecadienoylcarnitine[d]	M+H	ESI+	7	↑	2.53
102	6.40	357.2803	HMDB0002685	Prostaglandin F1a[c]	M+H	ESI+	47	↓	8.73
103	6.52	480.3068	HMDB0011130	LysoPE(18:0/0:0)[c]	M+H	ESI+	3	↓	1.30
104	6.60	516.3097	HMDB0000036	Taurocholic acid[b]	M+H	ESI+	21	↓	1.52
105	6.60	494.3124	HMDB0010383	LysoPC(16:1(9Z))[b]	/	ESI-&ESI+	24	↓	1.59
106	6.60	568.351	HMDB0010402	LysoPC(22:5(4Z,7Z,10Z,13Z,16Z))[d]	/	ESI-&ESI+	18	↓	1.44
107	6.68	398.3295	HMDB0006317	trans-Hexadec-2-enoyl carnitine[d]	M+H	ESI+	8	↑	1.73
108	6.75	520.3267	HMDB0010386	LysoPC(18:2(9Z,12Z))[c]	M+H	ESI+	25	↓	1.25
109	6.75	544.3364	HMDB0010395	LysoPC(20:4(5Z,8Z,11Z,14Z))[d]	M+H	ESI+	6	↓	1.46

NO .	[a]RT	Mass	HMDB number	Compounds	Adduct type	ESI mode	Delta (ppm)	Tendency (case/control)	Fold change
110	6.81	482.3221	HMDB0010381	LysoPC(15:0)[c]	/	ESI-&ESI+	4	↓	1.84
111	6.89	544.3219	HMDB0010396	LysoPC(20:4(8Z,11Z,14Z,17Z))[d]	/	ESI-&ESI+	35	↓	1.50
112	6.89	534.3571	HMDB0011491	LysoPE(0:0/22:1(13Z)) [d]	/	ESI-&ESI+	1	↓	1.52
113	7.06	508.3406	HMDB0011482	LysoPE(0:0/20:1(11Z)) [d]	M+H	ESI+	2	↓	1.97
114	7.10	464.315	HMDB0000331	3a,7b,12a-Trihydroxyoxocholanyl-Glycine[b]	M+FA-H	ESI-&ESI+	29	↓	1.36
115	7.26	546.3469	HMDB0010394	LysoPC(20:3(8Z,11Z,14Z))[d]	M+H	ESI+	16	↓	1.67
116	7.29	517.3906	HMDB0034227	alpha-Tocopherol acetate[d]	M+FA-H	ESI-	1	↓	1.67
117	7.36	277.2183	HMDB0003073	Gamma-Linolenic acid[b]	M-H	ESI-	4	↑	2.01
118	7.40	277.2199	HMDB0006547	Stearidonic acid[b]	M+H	ESI+	13	↑	4.70
119	7.64	572.3727	HMDB0010401	LysoPC(22:4(7Z,10Z,13Z,16Z))[d]	/	ESI-&ESI+	3	↓	1.32
120	7.77	391.2871	HMDB0000518	Chenodeoxycholic acid[b]	M-H	ESI-	4	↓	1.78
121	7.83	548.3687	HMDB0010392	LysoPC(20:2(11Z,14Z))[c]	M+H	ESI+	4	↓	1.62
122	7.84	530.3615	HMDB0013122	LysoPC(P-18:0)[d]	M+Na	ESI+	6	↓	1.26
123	7.86	293.2143	HMDB0010733	3-Oxohexadecanoic acid[d]	M+Na	ESI+	19	↑	2.49
124	7.91	510.3488	HMDB0012108	LysoPC(17:0)[b]	/	ESI-&ESI+	13	↓	1.63
125	8.17	327.2343	HMDB0002183	Docosahexaenoic acid[b]	M-H	ESI-	4	↑	1.46
126	8.19	391.2866	HMDB0000664	Isohyodeoxycholic acid[d]	M-H	ESI-	3	↓	1.74
127	8.28	524.3539	HMDB0011128	LysoPC(0:0/18:0)[c]	M+H	ESI+	33	↓	1.22
128	8.37	253.2186	HMDB0003229	Palmitoleic acid[b]	M-H	ESI-	5	↑	1.79
129	8.51	506.3566	HMDB0010408	LysoPC(P-18:1(9Z))[b]	M+H	ESI+	8	↓	1.20
130	8.59	524.3626	HMDB0010384	LysoPC(18:0)[b]	/	ESI-&ESI+	16	↓	1.70
131	8.69	550.3867	HMDB0010391	LysoPC(20:1(11Z))c	/	ESI-&ESI+	0	↓	1.44
132	8.98	808.5719	HMDB0008242	PC(18:4(6Z,9Z,12Z,15Z)/20:1(11Z))[d]	M+H	ESI+	16	↑	2.55
133	9.02	329.2501	HMDB0006528	Docosapentaenoic acid[b]	M-H	ESI-	5	↑	1.84
134	9.02	279.2266	HMDB0000673	Linoleic acid[b]	M-H	ESI-	23	↑	1.69
135	9.14	538.3888	HMDB0011490	LysoPE(0:0/22:0) [d]	/	ESI-&ESI+	4	↓	1.75
136	9.34	694.4669	HMDB0008885	PE(14:1(9Z)/P-18:1(11Z))[d]	M+Na	ESI+	16	↑	1.97
137	9.52	824.5801	HMDB0009211	PE(18:4(6Z,9Z,12Z,15Z)/24:0)[d]	M+H	ESI+	44	↑	4.27

NO.	aRT	Mass	HMDB number	Compounds	Adduct type	ESI mode	Delta (ppm)	Tendency (case/control)	Fold change
138	9.79	552.4039	HMDB0010390	LysoPC(20:0)[b]	M+H	ESI+	3	↓	1.48
139	10.18	255.2304	HMDB0000220	Palmitic acid[b]	M-H	ESI-	10	↑	1.46
140	10.12	331.2642	HMDB0002231	Eicosenoic acid[b]	M+Na-2H	ESI-&ESI+	7	↓	1.61
141	10.27	796.5497	HMDB0007955	PC(15:0/22:4(7Z,10Z,13Z,16Z))[d]	M+H	ESI+	44	↑	1.60
142	10.20	646.4139	HMDB0010405	LysoPC(24:0)[d]	M+K	ESI+	11	↑	1.69
143	10.28	836.5541	HMDB0010167	PS(18:0/22:6(4Z,7Z,10Z,13Z,16Z,19Z))[d]	M+H	ESI+	13	↑	1.80
144	10.37	281.2433	HMDB0000207	Oleic acid[b]	M-H	ESI-	19	↑	2.06
145	10.37	772.5507	HMDB0008330	PC(20:2(11Z,14Z)/15:0)[d]	M+H	ESI+	45	↑	1.81
146	10.31	812.5452	HMDB0010165	PS(18:0/20:4(8Z,11Z,14Z,17Z))[c]	M+H	ESI+	2	↑	1.87
147	10.34	790.551	HMDB0010163	PS(18:0/18:1(9Z))[c]	M+H	ESI+	10	↑	1.85
148	0.58	214.919	/	/	/	ESI+	/	↑	1.52
149	0.59	304.9141	/	/	/	ESI-	/	↑	1.72
150	0.63	693.0838	/	/	/	ESI-	/	↑	3.06
151	0.63	783.0272	/	/	/	ESI+	/	↑	3.00
152	0.66	286.8577	/	/	/	ESI-	/	↓	1.40
153	0.69	383.1186	/	/	/	ESI+	/	↑	1.95
154	0.70	365.1079	/	/	/	ESI+	/	↑	4.90
155	0.72	225.0838	/	/	/	ESI-	/	↑	1.88
156	0.72	359.0467	/	/	/	ESI+	/	↑	2.12
157	0.74	185.0922	/	/	/	ESI-	/	↑	2.57
158	0.74	291.0738	/	/	/	ESI+	/	↓	2.72
159	0.82	407.0429	/	/	/	ESI+	/	↓	2.25
160	0.84	291.0782	/	/	/	ESI-	/	↑	6.36
161	0.86	313.0636	/	/	/	ESI-&ESI+	/	↑	4.54
162	0.87	343.9938	/	/	/	ESI-	/	↑	2.61
163	0.96	215.0187	/	/	/	ESI+	/	↓	3.18
164	2.08	100.0653	/	/	/	ESI+	/	↓	7.79
165	2.10	202.0842	/	/	/	ESI+	/	↑	184.24

NO.	[a]RT	Mass	HMDB number	Compounds	Adduct type	ESI mode	Delta (ppm)	Tendency (case/control)	Fold change
166	2.61	415.2547	/	/	/	ESI+	/	↓	2.47
167	2.76	330.2037	/	/	/	ESI-	/	↑	5.76
168	2.77	243.1354	/	/	/	ESI-	/	↑	4.55
169	2.91	385.1694	/	/	/	ESI-	/	↑	2.35
170	2.95	652.4118	/	/	/	ESI+	/	↓	2.01
171	2.95	652.4118	/	/	/	ESI+	/	↓	15.36
172	3.18	383.1548	/	/	/	ESI-	/	↑	2.31
173	3.19	530.1236	/	/	/	ESI+	/	↑	93.77
174	3.30	331.1918	/	/	/	ESI-	/	↑	1.53
175	3.41	583.2572	/	/	/	ESI-	/	↓	2.99
176	3.90	359.2226	/	/	/	ESI-	/	↑	1.88
177	4.04	283.1908	/	/	/	ESI-	/	↑	2.32
178	4.09	311.2222	/	/	/	ESI-	/	↑	3.90
179	4.20	969.5724	/	/	/	ESI-	/	↓	6.86
180	4.53	981.599	/	/	/	ESI-	/	↓	2.08
181	4.71	554.3217	/	/	/	ESI-	/	↓	1.72
182	4.73	552.3327	/	/	/	ESI-	/	↓	2.45
183	4.87	502.2961	/	/	/	ESI-	/	↓	1.86
184	4.90	580.3178	/	/	/	ESI-	/	↓	1.32
185	5.25	999.6469	/	/	/	ESI-	/	↓	1.55
186	5.29	342.2664	/	/	/	ESI+	/	↑	2.52
187	5.75	489.3591	/	/	/	ESI-	/	↓	1.66
188	6.35	935.6134	/	/	/	ESI+	/	↓	19.36
189	6.40	518.3217	/	/	/	ESI+	/	↓	1.71
190	6.41	184.0746	/	/	/	ESI+	/	↓	2.46
191	6.60	987.644	/	/	/	ESI+	/	↓	3.83
192	6.90	584.3497	/	/	/	ESI-	/	↓	1.54
193	7.58	504.3435	/	/	/	ESI+	/	↓	1.53
194	7.82	464.3168	/	/	/	ESI+	/	↓	1.34

NO.	ªRT	Mass	HMDB number	Compounds	Adduct type	ESI mode	Delta (ppm)	Tendency (case/control)	Fold change
195	7.90	532.3409	/	/	/	ESI+	/	↓	1.70
196	8.21	666.4349	/	/	/	ESI+	/	↑	2.32
197	8.48	464.3177	/	/	/	ESI+	/	↓	1.39
198	8.52	184.0745	/	/	/	ESI+	/	↓	1.49
199	10.08	467.3719	/	/	/	ESI-	/	↓	1.82
200	10.34	381.1713	/	/	/	ESI-	/	↑	1.95

[a]RT, retention time

[b]metabolites confirmed by standards.

[c]metabolites identified by MS/MS spectra and MS fragmentation.

[d]possible elemental compositions identified based on MS fragmentation, exact mass.

S.Table 3. Demographic and pathological characteristics of fifty-one colorectal cancer patients in the paired tissue metabolomics study

No.	Sex	Age	Location	T Stage	N Stage	M Stage	Overall stage	Pathological Differentiation
1	Female	55	Distal colon	T4	N0	M0	IIIB	Moderate
2	Male	67	Sigmoid colon	T4	N0	M0	IIIB	Moderate
3	Male	59	Sigmoid colon	T4	N0	M0	IIIB	Moderate
4	Female	60	Rectum	T4	N1	M0	IIIB	Moderate
5	Male	52	Rectum	T3	N0	M0	IIB	Moderate
6	Male	56	Descending colon	T4	N0	M0	IIIB	Moderate
7	Female	74	Spleen area	T3	N0	M0	IIB	Moderate
8	Female	57	Sigmoid colon	T4	N0	M0	IIIB	Moderate
9	Female	50	Sigmoid colon	T3	N0	M0	IIB	Poor
10	Male	65	Rectum	T3	N1	M0	IIIA	Moderate
11	Male	55	Rectum	T3	N0	M0	IIB	Moderate
12	Male	54	Sigmoid colon	T3	N0	M0	IIB	Moderate
13	Male	62	Sigmoid colon	T4	N0	M1	IV	Moderate
14	Female	71	Sigmoid colon	T3	N1	M0	IIIA	Moderate
15	Male	45	Ascending colon	T3	N1	M0	IIIA	Moderate
16	Male	38	Sigmoid colon	T4	N0	M1	IV	Poor-Moderate
17	Female	56	Rectum	T4	N1	M0	IIIB	Moderate
18	Male	56	Ascending colon	T4	N1	M0	IIIB	Moderate
19	Female	74	Transverse colon	T3	N1	M0	IIIA	Moderate

No.	Sex	Age	Location	T Stage	N Stage	M Stage	Overall stage	Pathological Differentiation
20	Male	61	Sigmoid colon	T4	N1	M0	IIIB	Moderate
21	Female	44	Rectum	T3	N1	M0	IIIA	Poor-Moderate
22	Male	56	Rectum	T4	N1	M0	IIIB	Poor.Moderate
23	Male	66	Rectum	T3	N0	M0	IIB	Moderate
24	Male	48	Sigmoid colon	T3	N0	M0	IIB	Moderate
25	Female	62	Rectum	T3	N1	M0	IIIA	Poor
26	Male	60	Spleen area	T3	N1	M0	IIIA	Poor.Moderate
27	Female	72	Cecum	T4	N0	M0	IIIB	Moderate
28	Male	49	Rectum	T3	N1	M0	IIIA	Poor.Moderate
29	Male	79	Rectum	T3	N0	M0	IIB	Moderate
30	Female	57	Sigmoid colon	T3	N1	M0	IIIA	High
31	Female	68	Ascending colon	T4	N1	M0	IIIB	Poor
32	Male	72	Liver area	T3	N0	M1	IV	Moderate
33	Female	75	Rectum	T4	N0	M0	IIIB	Moderate
34	Male	65	Spleen area	T3	N0	M0	IIB	Moderate
35	Male	73	Rectum	T3	N0	M0	IIB	Moderate
36	Female	68	Rectum	T2	N0	M0	IIA	Moderate
37	Female	70	Rectum	T2	N0	M0	IIA	Moderate
38	Male	56	Rectum	T3	N0	M0	IIB	Moderate
39	Female	50	Rectum	T4	N0	M0	IIIB	Poor-Moderate
40	Female	39	Rectum	T1	N0	M0	I	High

No.	Sex	Age	Location	T Stage	N Stage	M Stage	Overall stage	Pathological Differentiation
41	Male	56	Rectum	T4	N1	M0	IIIB	Moderate
42	Male	47	Distal colon	T3	N0	M0	IIB	Moderate
43	Male	59	Transverse colon	T4	N0	M0	IIIB	Moderate
44	Female	22	Proximal colon	T5	N0	M1	IV	Poor
45	Female	62	rectum	T3	N1	M0	IIIA	Moderate
46	Female	58	rectum	T2	N0	M0	IIA	Moderate
47	Female	39	Cecum	T4	N0	M0	IIIB	Moderate
48	Female	39	rectum	T1	N0	M0	I	High
49	Male	45	/	T2	/	/	/	Poor
50	Male	34	/	T1	N0	/	/	High
51	Male	/	/	/	/	/	/	/

Note: Missing data: age,1; tissue location, 3; N Stage, 2; M Stage, 3; TNM Stage, 3; Pathological differentiation, 1.

S.Table 4. Colorectal cancer metabolites identified from the paired tissue metabolomics study

NO.	RT[a]	Mass	HMDB ID	Name	Adduct	ESI	Delta (ppm)	Changing Trend	Overlapped[b]	Fold-change
1	0.63	333.0537	HMDB0000229	Nicotinamide ribotide	M-H	ESI-	11	↑	no	2.09
2	0.71	242.0382	HMDB0001254	Glucosamine 6-phosphate	M-H2O+H	ESI+	20	↑	no	1.77
3	0.78	450.9788	HMDB0003335	IDP	M+Na	ESI+	6	↑	yes	2.36
4	0.97	231.1684	HMDB0028920	Isoleucyl-Valine	M+H	ESI+	8	↑	no	2.26
5	0.97	322.0385	HMDB0000095	Cytidine monophosphate	M-H	ESI-	19	↑	no	1.85
6	1.00	323.0237	HMDB0060282	3'-UMP	M-H	ESI-	15	↑	no	2.81
7	1.00	352.0486	HMDB0001202	dCMP	M+FA-H	ESI-	19	↑	no	2.91
8	1.02	146.0971	HMDB0003464	4-Guanidinobutanoic acid	M+H	ESI+	32	↑	no	1.75
9	1.05	345.0069	HMDB0000288	Uridine 5'-monophosphate	M+Na-2H	ESI-/ESI+	11	↑	no	2.26
10	1.06	249.0534	HMDB0001555	Pyridoxamine 5'-phosphate	M+H	ESI+	40	↑	no	4.58
11	1.07	199.1019	HMDB0029027	Prolyl-Threonine	M-H2O+H	ESI+	32	↑	no	1.88
12	1.09	227.0768	HMDB0000929	L-Tryptophan	M+Na	ESI+	10	↑	yes	3.20
13	1.11	204.0868	HMDB0000212	N-Acetylgalactosamine	M-H2O+H	ESI+	2	↑	no	3.45
14	1.14	256.1012	HMDB0002275	7,8-Dihydroneopterin	M+H	ESI+	11	↑	no	5.32
15	1.18	148.0601	HMDB0001566	3-Methylguanine	M-H2O+H	ESI+	15	↑	no	1.72
16	1.27	213.0222	HMDB0001548	D-Ribose 5-phosphate	M-H2O+H	ESI+	27	↑	no	2.50
17	1.28	181.0722	HMDB0000143	D-Galactose	M+H	ESI+	8	↑	yes	3.60
18	1.84	205.0836	HMDB0011162	L-beta-aspartyl-L-alanine	M+H	ESI+	8	↑	no	8.06
19	1.86	136.0602	HMDB0000034	Adenine	M+H	ESI+	12	↑	no	2.04
20	1.88	374.0459	HMDB0000058	Cyclic AMP	M+FA-H	ESI-	13	↑	no	1.73
21	1.89	175.1403	HMDB0013287	Ne,Ne dimethyllysine	M+H	ESI+	22	↑	no	2.74
22	1.89	210.9929	HMDB0000618	D-Ribulose 5-phosphate	M-H20-H	ESI-	37	↑	no	1.55
23	1.90	164.0564	HMDB0002273	4-Hydroxy-L-glutamic acid	M+H	ESI+	6	↑	no	1.54
24	1.91	434.0699	HMDB0006268	N-Acetylneuraminate 9-phosphate	M+FA-H	ESI-	1	↑	no	1.76
25	1.91	447.1004	HMDB0001117	4-Phosphopantothenoylcysteine	M+FA-H	ESI-	36	↑	no	1.74
26	1.93	148.0606	HMDB0002393	N-Methyl-D-aspartic acid	M+H	ESI+	1	↑	no	1.54
27	1.93	219.0986	HMDB0006248	gamma-Glutamylalanine	M+H	ESI+	5	↑	no	2.83
28	1.94	173.0497	HMDB0001212	Hydantoin-5-propionic acid	M+H	ESI+	35	↓	no	1.47

NO.	RT[a]	Mass	HMDB ID	Name	Adduct	ESI	Delta (ppm)	Changing Trend	Overlapped[b]	Fold-change
29	1.97	240.0907	HMDB0002224	5 Methyldeoxycytidine	M-H	ESI+/ESI-	34	↑	no	1.96
30	1.97	273.1193	HMDB0000052	Argininosuccinic acid	M-H2O+H	ESI+	2	↑	no	3.02
31	1.99	160.1305	HMDB0000991	DL-2-Aminooctanoic acid	M+H	ESI+	17	↑	no	1.72
32	2.01	376.0652	HMDB0000905	Deoxyadenosine monophosphate	M+FA-H	ESI-	3	↑	no	3.09
33	2.01	422.1492	HMDB0001185	S-Adenosylmethionine	M+Na	ESI+	13	↑	no	4.70
34	2.02	348.0849	HMDB0003540	3'-AMP	M+H	ESI+	42	↑	yes	1.72
35	2.03	835.2475	HMDB0001086	Uroporphyrinogen III	M-H	ESI-	24	↑	no	17.61
36	2.08	312.1423	HMDB0004824	N2，N2-Dimethylguanosine	M+H	ESI+	39	↑	no	2.11
37	2.13	114.0552	HMDB0000725	4-Hydroxyproline	M-H2O+H	ESI+	3	↑	yes	1.67
38	2.14	304.0941	HMDB0003950	7-Methylinosine	M+Na-2H	ESI-	5	↑	no	2.01
39	2.20	241.0965	HMDB0001238	N-Acetylserotonin	M+Na	ESI+	7	↑	no	2.20
40	2.20	326.151	HMDB0029143	gamma-Glutamylarginine	M+Na	ESI+	23	↑	no	2.33
41	2.24	131.0511	HMDB0002048	m-Cresol	M+Na	ESI+	33	↑	yes	9.93
42	2.27	488.2868	HMDB0000637	Glycochenodeoxycholate	M+K	ESI+	19	↑	yes	1.67
43	2.31	319.1003	HMDB0001107	7-Methylguanosine	M+Na-2H	ESI-	16	↑	yes	6.90
44	2.33	178.0894	HMDB0001855	5 Hydroxytryptophol	M+H	ESI+	18	↑	no	1.67
45	2.37	233.0643	HMDB0001049	gamma-Glutamylcysteine	M-H2O+H	ESI+	20	↓	no	2.93
46	2.38	512.2458	HMDB0002497	Glycochenodeoxycholate-3-sulfate	M-H2O+H	ESI+	44	↑	no	1.45
47	2.41	371.1781	HMDB0031031	2-Dodecylbenzenesulfonic acid	M+FA-H	ESI-/ESI+	31	↑	no	5.44
48	2.52	174.0921	HMDB0004225	2-Oxoarginine	M+H	ESI+	27	↑	no	2.64
49	2.54	233.1298	HMDB0000670	Homo-L-arginine	M+FA-H	ESI-/ESI+	18	↓	no	2.05
50	2.68	157.124	HMDB0031513	3-hydroxynonanoic acid	M+H	ESI+	7	↑	no	1.49
51	2.75	127.0697	HMDB0002207	3-Hydroxyisoheptanoic acid	M-H20-H	ESI-	49	↓	no	2.46
52	2.76	239.0907	HMDB0028853	Glycyltyrosine	M+H	ESI+/ESI-	50	↓	no	1.75
53	2.86	195.0843	HMDB0013704	6-Methylnicotinamide	M+FA-H	ESI+/ESI-	35	↓	no	2.14
54	2.90	162.0613	HMDB0000714	Hippuric acid	M-H2O+H	ESI+	36	↑	yes	1.82
55	2.91	130.0823	HMDB0000070	Pipecolic acid	M+H	ESI+	30	↑	no	1.58
56	2.98	213.0968	HMDB0004224	N-(o)-Hydroxyarginine	M+Na	ESI-/ESI+	5	↓	no	1.95
57	3.12	522.2927	HMDB0000951	Taurochenodesoxycholic acid	M+Na	ESI+	13	↑	yes	3.61

NO.	RT[a]	Mass	HMDB ID	Name	Adduct	ESI	Delta (ppm)	Changing Trend	Overlapped[b]	Fold-change
58	3.28	243.1162	HMDB0011177	Phenylalanylproline	M-H20-H	ESI-	12	↓	no	2.98
59	3.29	295.1187	HMDB0000594	gamma-Glutamylphenylalanine	M-H	ESI-	14	↓	no	3.03
60	3.30	249.0978	HMDB0004185	5 Hydroxyindoleacetylglycine	M+H	ESI+	43	↓	no	1.74
61	3.49	261.1268	HMDB0003426	Pantetheine	M-H2O+H	ESI+	2	↑	no	1.68
62	3.62	569.3181	HMDB0002596	Deoxycholic acid 3-glucuronide	M+H	ESI+	24	↑	yes	2.32
63	3.70	379.235	HMDB0002082	Bisnorcholic acid	M-H	ESI-	37	↑	no	3.43
64	3.72	229.1247	HMDB0011166	L-beta-aspartyl-L-leucine	M-H2O+H	ESI+	25	↑	no	1.42
65	3.75	349.198	HMDB0002183	Docosahexaenoic acid	M+Na-2H	ESI-/ESI+	48	↑	yes	2.99
66	4.01	293.2042	HMDB0003871	13-L-Hydroperoxylinoleic acid	M-H20-H	ESI-	25	↑	yes	3.15
67	4.31	365.2281	HMDB0003876	15(S)-HETE	M+FA-H	ESI-	14	↑	no	10.97
68	4.40	482.3197	HMDB0000648	Galactosylsphingosine	M+Na-2H	ESI-	20	↑	no	1.79
69	4.51	464.3115	HMDB0000138	Glycocholic acid	M-H	ESI-	21	↑	yes	4.41
70	4.54	353.2269	HMDB0001220	Prostaglandin E2	M+H	ESI-/ESI+	15	↓	no	1.33
71	4.86	295.2168	HMDB0000782	Octadecanedioic acid	M-H20-H	ESI-	36	↑	no	1.59
72	5.02	167.1004	HMDB0010724	3-Oxodecanoic acid	M-H20-H	ESI-	41	↑	no	3.66
73	5.29	508.34	HMDB0012108	LysoPC(17:0)	M-H	ESI-	2	↓	yes	2.79
74	5.37	284.2157	HMDB0013250	Myristoylglycine	M-H	ESI-	26	↑	no	1.40
75	5.43	271.2193	HMDB0010734	(R)-3-Hydroxy-hexadecanoic acid	M-H	ESI-	32	↓	yes	1.81
76	6.01	496.3322	HMDB0010382	LysoPC(16:0)	M+H	ESI+	15	↑	yes	6.80
77	6.44	524.3651	HMDB0010384	LysoPC(18:0)	M+H	ESI+	11	↓	yes	1.68
78	6.93	546.3508	HMDB0013122	LysoPC(P-18:0)	M+K	ESI+	34	↑	yes	14.68
79	7.00	257.2197	HMDB0061657	3-hydroxypentadecanoic acid	M-H	ESI-	29	↑	no	5.91
80	7.00	301.2106	HMDB0000673	Linoleic acid	M+Na-2H	ESI-	14	↑	yes	5.62
81	7.25	277.2094	HMDB0003073	Gamma-Linolenic acid	M-H20-H	ESI-	29	↑	yes	2.91
82	7.26	277.2136	HMDB0004669	9-OxoODE	M-H2O+H	ESI+/ESI-	12	↑	no	3.88
83	7.49	231.203	HMDB0061655	3-hydroxytridecanoic acid	M+H	ESI+	33	↑	no	1.85
84	7.49	295.2278	HMDB0004668	13-OxoODE	M-H	ESI-	0	↑	yes	5.50
85	7.93	414.3224	HMDB0013333	3-Hydroxy-9-hexadecenoylcarnitine	M+H	ESI+	2	↓	yes	2.15
86	8.53	361.1857	HMDB0000319	18 Hydroxycorticosterone	M-H	ESI-	45	↑	no	1.96

NO.	RT[a]	Mass	HMDB ID	Name	Adduct	ESI	Delta (ppm)	Changing Trend	Overlapped[b]	Fold-change
87	9.07	329.245	HMDB0002925	8,11,14-Eicosatrienoic acid	M+Na	ESI+	0	↑	no	4.78
88	9.29	221.1516	HMDB0000529	5-Dodecenoic acid	M+Na	ESI+/ESI-	2	↑	no	3.82
89	9.42	331.2588	HMDB0006528	Docosapentaenoic acid	M+H	ESI+	13	↑	yes	4.40
90	9.46	267.2256	HMDB0002172	N1,N12-Diacetylspermine	M-H20-H	ESI-	27	↑	no	2.34
91	9.54	305.2399	HMDB0010378	5,8,11-Eicosatrienoic acid	M-H	ESI-	29	↑	no	2.93
92	9.95	313.2681	HMDB0060043	13-HDoHE	M-H	ESI-	46	↓	no	1.96
93	10.05	331.2562	HMDB0002231	Eicosenoic acid	M-H	ESI-	17	↑	yes	2.36
94	1.01	210.9931	/	/	/	ESI-	/	↑	/	3.15
95	1.84	223.0934	/	/	/	ESI+	/	↑	/	2.74
96	1.88	346.0483	/	/	/	ESI-	/	↑	/	1.89
97	1.89	348.0664	/	/	/	ESI+	/	↑	/	2.03
98	1.90	376.0606	/	/	/	ESI-	/	↑	/	2.14
99	1.90	378.0786	/	/	/	ESI+	/	↑	/	2.21
100	1.91	417.0904	/	/	/	ESI-	/	↑	/	2.53
101	1.92	237.1095	/	/	/	ESI+	/	↑	/	2.05
102	1.92	449.1183	/	/	/	ESI+	/	↑	/	2.70
103	1.99	114.1213	/	/	/	ESI+	/	↑	/	1.47
104	2.00	390.0764	/	/	/	ESI-	/	↑	/	2.11
105	2.01	210.9933	/	/	/	ESI-	/	↑	/	2.07
106	2.02	392.0974	/	/	/	ESI+	/	↑	/	2.42
107	2.22	194.1160	/	/	/	ESI+	/	↑	/	2.09
108	2.23	148.1056	/	/	/	ESI+	/	↑	/	1.87
109	2.52	144.0809	/	/	/	ESI+	/	↑	/	4.56
110	3.00	388.1507	/	/	/	ESI+	/	↑	/	2.00
111	3.26	827.4454	/	/	/	ESI-	/	↑	/	2.54
112	3.44	436.1966	/	/	/	ESI-	/	↑	/	2.52
113	3.48	387.2142	/	/	/	ESI-	/	↓	/	1.58
114	3.64	355.2401	/	/	/	ESI-	/	↑	/	2.19
115	5.65	323.2506	/	/	/	ESI-	/	↑	/	2.67

NO.	RT[a]	Mass	HMDB ID	Name	Adduct	ESI	Delta (ppm)	Changing Trend	Overlapped[b]	Fold-change
116	6.65	498.3490	/	/	/	ESI+	/	↑	/	6.44
117	7.60	293.1713	/	/	/	ESI-	/	↓	/	1.61
118	7.91	722.5008	/	/	/	ESI+	/	↓	/	1.60
119	8.03	283.2352	/	/	/	ESI-	/	↑	/	6.96
120	8.23	253.2086	/	/	/	ESI-	/	↑	/	2.31
121	8.53	259.2356	/	/	/	ESI-	/	↑	/	2.83
122	8.53	303.2213	/	/	/	ESI-	/	↑	/	2.59
123	9.29	305.2451	/	/	/	ESI+	/	↑	/	2.95

Note: RT[a], retention time. [b]overlapped, the differential metabolites overlapped with that identified from plasma.

S.Table 5. Differential metabolites and reported frequency in previous metabolomics study of colorectal cancer

Metabolite Name	HMDB ID	Frequency	Metabolite Name	HMDB ID	Frequency
Glycine	HMDB0000123	22	3-Oxodecanoic acid	HMDB0010724	1
L-Valine	HMDB0000883	22	3-Phosphoglyceric acid	HMDB0000807	1
L-Lactic acid	HMDB0000190	21	Acetaminophen	HMDB0001859	1
L-Alanine	HMDB0000161	20	4-Amino-1-piperidinecarboxylic acid	HMDB0060385	1
L-Phenylalanine	HMDB0000159	20	4-Aminohippuric acid	HMDB0001867	1
L-Leucine	HMDB0000687	19	4-Hydroxy-5-(dihydroxyphenyl)-valeric	HMDB0059977	1
L-Glutamic acid	HMDB0000148	17	acid-O-methyl-O-sulphate	HMDB0000710	1
L-Proline	HMDB0000162	18	4-Hydroxybutyric acid	HMDB0004072	1
Taurine	HMDB0000251	15	4-Hydroxystyrene	HMDB0033647	1
L-Aspartic acid	HMDB0000191	14	5,6:8,9-Diepoxyergost-22-ene-3,7beta-diol	HMDB0001235	1
L-Isoleucine	HMDB0000172	13	5-Aminoimidazole ribonucleotide	HMDB0000763	1
L-Methionine	HMDB0000696	14	5-Hydroxyindoleacetic acid	HMDB0000259	1
Palmitic acid	HMDB0000220	14	Serotonin	HMDB0001316	1
L-Tyrosine	HMDB0000158	13	6-Phosphogluconic acid	HMDB0000501	1
L-Serine	HMDB0000187	13	7-Ketocholesterol	HMDB0000391	1
L-Threonine	HMDB0000167	13	7-Ketodeoxycholic acid	HMDB0003333	1
Choline	HMDB0000097	11	8-Hydroxy-deoxyguanosine	HMDB0010223	1
D-Glucose	HMDB0000122	12	9-HODE	HMDB0004669	1
L-Glutamine	HMDB0000641	11	9-OxoODE	HMDB0001659	1
Glycerol	HMDB0000131	12	Acetone	HMDB0000448	1
L-Arginine	HMDB0000517	11	Adipic acid	HMDB0001539	1
L-Asparagine	HMDB0000168	10	Asymmetric dimethylarginine	HMDB0028689	1
L-Lysine	HMDB0000182	11	Alanyl-Histidine	HMDB0028691	1
myo-Inositol	HMDB0000211	11	Alanyl-Leucine	HMDB0028698	1

Metabolite Name	HMDB ID	Frequency	Metabolite Name	HMDB ID	Frequency
Acetic acid	HMDB0000042	10	Alanyl-Tryptophan	HMDB0001209	1
Myristic acid	HMDB0000806	10	Allantoic acid	HMDB0000557	1
Succinic acid	HMDB0000254	10	L-Alloisoleucine	HMDB0000381	1
L-Tryptophan	HMDB0000929	10	Allolithocholic acid	HMDB0005843	1
Uracil	HMDB0000300	9	Allyl isothiocyanate	HMDB0028832	1
Uridine	HMDB0000296	10	Glutamylvaline	HMDB0001149	1
3-Hydroxybutyric acid	HMDB0000357	9	5-Aminolevulinic acid	HMDB0028729	1
Citric acid	HMDB0000094	9	Asparaginyl-Glutamine	HMDB0004985	1
L-Cysteine	HMDB0000574	8	Aspartylysine	HMDB0000784	1
scyllo-Inositol	HMDB0006088	8	Azelaic acid	HMDB0006115	1
Creatine	HMDB0000064	8	Benzaldehyde	HMDB0011168	1
Glutathione	HMDB0000125	7	L-beta-aspartyl-L-serine	HMDB0003640	1
L-Histidine	HMDB0000177	8	Beta-Leucine	HMDB0000852	1
Linoleic acid	HMDB0000673	8	Beta-Sitosterol	HMDB0013126	1
Oleic acid	HMDB0000207	8	Butenylcarnitine	HMDB0002013	1
Ornithine	HMDB0000214	8	Butyrylcarnitine	HMDB0013207	1
Pyruvic acid	HMDB0000243	8	9-Hexadecenoylcarnitine	HMDB0062703	1
Stearic acid	HMDB0000827	8	Octadec-9-enoic Acid	HMDB0013124	1
L-Alpha-aminobutyric acid	HMDB0000452	7	Propenoylcarnitine	HMDB0013125	1
2-Hydroxybutyric acid	HMDB0000008	7	Hydroxypropionylcarnitine	HMDB0000518	1
Creatinine	HMDB0000562	7	Chenodeoxycholic acid	HMDB0001413	1
Fumaric acid	HMDB0000134	7	Citicoline	HMDB0000634	1
Alpha-Linolenic acid	HMDB0001388	6	Citraconic acid	HMDB0000426	1
L-Palmitoylcarnitine	HMDB0000222	7	Citramalic acid	HMDB0005047	1
4-Hydroxyproline	HMDB0000725	6	9E,11E-Octadecadienoic acid	HMDB0001218	1

Metabolite Name	HMDB ID	Frequency	Metabolite Name	HMDB ID	Frequency
Arachidonic acid	HMDB0001043	6	Coumarin	HMDB0003315	1
Cholesterol	HMDB0000067	6	Cyclohexanone	HMDB0000656	1
Glycerophosphocholine	HMDB0000086	6	Cysteineglutathione disulfide	HMDB0000651	1
Hippuric acid	HMDB0000714	6	Decanoylcarnitine	HMDB0000626	1
Hypoxanthine	HMDB0000157	6	Deoxycholic Acid	HMDB0004437	1
Pyroglutamic acid	HMDB0000267	5	Diethanolamine	HMDB0004983	1
L-Acetylcarnitine	HMDB0000201	5	Dimethyl sulfone	HMDB0000087	1
Beta-Alanine	HMDB0000056	5	Dimethylamine	HMDB0001976	1
Citrulline	HMDB0000904	5	Docosapentaenoic acid (22n-6)	HMDB0000073	1
L-Cystine	HMDB0000192	5	Dopamine	HMDB0001548	1
D-Galactose	HMDB0000143	5	D-Ribose 5-phosphate	HMDB0011740	1
Elaidic acid	HMDB0000573	5	Turanose	HMDB0014789	1
Inosine	HMDB0000195	4	Dyphylline	HMDB0002925	1
L-Malic acid	HMDB0000156	5	8,11,14-Eicosatrienoic acid	HMDB0006464	1
Malic acid	HMDB0000744	5	Elaidic carnitine	HMDB0000068	1
Oleamide	HMDB0002117	5	Epinephrine	HMDB0000149	1
Phosphate	HMDB0001429	5	Ethanolamine	HMDB0037620	1
Trimethylamine N-oxide	HMDB0000925	5	Ethyl 4-(methylthio)butyrate	HMDB0031217	1
L-Arabinose	HMDB0000646	4	Ethyl acetate	HMDB0031231	1
Ascorbic acid	HMDB0000044	4	xi-2-Ethyl-1-hexanol	HMDB0000622	1
Beta-D-Glucose	HMDB0000516	4	Ethylmalonic acid	HMDB0000124	1
Betaine	HMDB0000043	4	Fructose 6-phosphate	HMDB0001586	1
L-Carnitine	HMDB0000062	4	Glucose 1-phosphate	HMDB0005826	1
Dodecanoic acid	HMDB0000638	4	Galactinol	HMDB0001492	1
Formic acid	HMDB0000142	4	Gamma-Tocopherol	HMDB0028796	1

Metabolite Name	HMDB ID	Frequency	Metabolite Name	HMDB ID	Frequency
D-Fructose	HMDB0000660	4	Glutaminylglutamic acid	HMDB0003514	1
Glyceric acid	HMDB0000139	4	Alpha-D-Glucose 1,6-bisphosphate	HMDB0028818	1
Glycolic acid	HMDB0000115	4	Glutamylglutamic acid	HMDB0000661	1
Isocitric acid	HMDB0000193	4	Glutaric acid	HMDB0031075	1
Heptadecanoic acid	HMDB0002259	4	Glycerol 1-octadecanoate	HMDB0011533	1
Palmitoleic acid	HMDB0003229	4	Glyceryl 2-palmitate	HMDB0000114	1
Pantothenic acid	HMDB0000210	4	Glycerylphosphorylethanolamine	HMDB0003344	1
Cytidine monophosphate	HMDB0000095	4	Glycolaldehyde	HMDB0002639	1
Phosphorylcholine	HMDB0001565	4	Sulfolithocholylglycine	HMDB0000708	1
Ribitol	HMDB0000508	4	Glycoursodeoxycholic acid	HMDB0028854	1
Threonic acid	HMDB0000943	4	Glycyl-Valine	HMDB0001397	1
Xanthine	HMDB0000292	4	Guanosine monophosphate	HMDB0000128	1
Eicosadienoic acid	HMDB0005060	2	Guanidoacetic acid	HMDB0003178	1
Monooleoylglycerol	HMDB0094684	3	Heme	HMDB0000672	1
Acetoacetic acid	HMDB0000060	3	Hexadecanedioic acid	HMDB0000535	1
Adenine	HMDB0000034	3	Caproic acid	HMDB0003431	1
Adenosine	HMDB0000050	3	L-Histidinol	HMDB0000745	1
Oxoglutaric acid	HMDB0000208	3	Homocarnosine	HMDB0000719	1
Alpha-Tocopherol	HMDB0001893	3	L-Homoserine	HMDB0002434	1
Adenosine monophosphate	HMDB0000045	3	Hydroquinone	HMDB0000738	1
Benzoic acid	HMDB0001870	3	Indole	HMDB0000734	1
cis-Aconitic acid	HMDB0000072	3	Indoleacrylic acid	HMDB0004094	1
Cytidine	HMDB0000089	3	Indoxyl	HMDB0002985	1
Capric acid	HMDB0000511	3	Inositol phosphate	HMDB0003903	1
Dimethylglycine	HMDB0000092	3	2-Hydroxyethanesulfonate	HMDB0006009	1

Metabolite Name	HMDB ID	Frequency	Metabolite Name	HMDB ID	Frequency
D-Mannose	HMDB0000169	3	Isoputreanine	HMDB0000718	1
Glyceraldehyde	HMDB0001051	3	Isovaleric acid	HMDB0000715	1
Glycerol 3-phosphate	HMDB0000126	3	Kynurenic acid	HMDB0040937	1
Glycocholic acid	HMDB0000138	3	Lactitol	HMDB0028922	1
Isobutyric acid	HMDB0001873	3	Leucyl-Alanine	HMDB0028928	1
L-Kynurenine	HMDB0000684	3	Leucyl-Glutamate	HMDB0028933	1
Tetracosanoic acid	HMDB0002003	3	Leucyl-Leucine	HMDB0011175	1
LysoPC(16:0)	HMDB0010382	3	Leucylproline	HMDB0028938	1
LysoPC(14:0)	HMDB0010379	3	Leucyl-Serine	HMDB0028940	1
Palmitic amide	HMDB0012273	3	Leucyl-Tryptophan	HMDB0000174	1
O-Phosphoethanolamine	HMDB0000224	2	L-Fucose	HMDB0007850	1
Pelargonic acid	HMDB0000847	3	LysoPA(0:0/18:0)	HMDB0007854	1
Phenylacetic acid	HMDB0000209	3	LysoPA(18:0/0:0)	HMDB0010384	1
Alpha-N-Phenylacetyl-L-glutamine	HMDB0006344	3	LysoPC(18:0)	HMDB0010387	1
Propionic acid	HMDB0000237	3	LysoPC(18:3(6Z,9Z,12Z))	HMDB0010393	1
Putrescine	HMDB0001414	3	LysoPC(20:3(5Z,8Z,11Z))	HMDB0000667	1
S-Adenosylhomocysteine	HMDB0000939	3	L-Thyronine	HMDB0010405	1
Sarcosine	HMDB0000271	3	LysoPC(24:0)	HMDB0029205	1
Urea	HMDB0000294	3	lysoPC(26:0)	HMDB0029220	1
Uric acid	HMDB0000289	3	lysoPC(26:1(5Z))	HMDB0029206	1
D-Xylose	HMDB0000098	3	lysoPC(28:0)	HMDB0029207	1
11-Eicosenoic acid	HMDB0034296	2	lysoPC(6:0)	HMDB0010383	1
13S-hydroxyoctadecadienoic acid	HMDB0004667	2	LysoPC(16:1(9Z))	HMDB0010390	1
1,5-Anhydrosorbitol	HMDB0002712	2	LysoPC(20:0)	HMDB0010408	1
Deoxyuridine	HMDB0000012	2	LysoPC(P-18:1(9Z))	HMDB0000176	1

Metabolite Name	HMDB ID	Frequency	Metabolite Name	HMDB ID	Frequency
Aminoadipic acid	HMDB0000510	2	Maleic acid	HMDB0000703	1
2-Hydroxy-3-methylpentanoic acid	HMDB0000317	2	Mandelic acid	HMDB0011657	1
3-Hydroxyisovaleric acid	HMDB0000754	2	2,6-Diamino-4-hydroxy-5-N-methylformamido	HMDB0000639	1
3-Methylhistidine	HMDB0000479	2	pyrimidine	HMDB0005066	1
Gamma-Aminobutyric acid	HMDB0000112	2	Galactaric acid	HMDB0002172	1
4-Pyridoxic acid	HMDB0000017	2	Tetradecanoylcarnitine	HMDB0000439	1
5-Methylcytidine	HMDB0000982	2	N1,N12-Diacetylspermine	HMDB0001562	1
Alpha-D-Glucose	HMDB0003345	2	2-Furoylglycine	HMDB0004044	1
Aminomalonic acid	HMDB0001147	2	N5-Formyl-THF	HMDB0001238	1
D-Arabitol	HMDB0000568	2	N6-Methyladenosine	HMDB0000766	1
Arachidic acid	HMDB0002212	2	N-Acetylserotonin	HMDB0000812	1
Behenic acid	HMDB0000944	2	N-Acetyl-L-alanine	HMDB0006029	1
Butyric acid	HMDB0000039	2	N-Acetyl-L-aspartic acid	HMDB0000446	1
11Z-Eicosenoic acid	HMDB0002231	1	N-Acetylglutamine	HMDB0002931	1
Cholic acid	HMDB0000619	2	N-Alpha-acetyllysine	HMDB0000221	1
CPA(18:0/0:0)	HMDB0007004	2	N-Acetylserine	HMDB0001488	1
L-Cystathionine	HMDB0000099	2	NADPH	HMDB0029595	1
Cytosine	HMDB0000630	1	Nicotinic acid	HMDB0001645	1
Ethanol	HMDB0000108	2	Nonane	HMDB0012267	1
Gluconic acid	HMDB0000625	2	L-Norleucine	HMDB0032549	1
D-Glucuronic acid	HMDB0000127	2	N-Succinyl-L,L-2,6-diaminopimelate	HMDB0000782	1
Glutarylcarnitine	HMDB0013130	2	N-Undecylbenzenesulfonic acid	HMDB0000848	1
Glycerol 1-hexadecanoate	HMDB0031074	2	Octadecanedioic acid	HMDB0000893	1
Glycochenodeoxycholate	HMDB0000637	2	Stearoylcarnitine	HMDB0005765	1
Homocysteine	HMDB0000742	2	Suberic acid	HMDB0000226	1

Metabolite Name	HMDB ID	Frequency	Metabolite Name	HMDB ID	Frequency
Homovanillic acid	HMDB0000118	2	Ophthalmic acid	HMDB0000788	1
Hypotaurine	HMDB0000965	2	Orotic acid	HMDB0002329	1
Indoleacetic acid	HMDB0000197	2	Orotidine	HMDB0010316	1
Indoxyl sulfate	HMDB0000682	2	Oxalic acid	HMDB0012328	1
LysoPC(18:2(9Z,12Z))	HMDB0010386	2	Acetaminophen glucuronide	HMDB0061712	1
LysoPC(18:1(9Z))	HMDB0002815	2	Palmitelaidic acid	HMDB0062802	1
Malonic acid	HMDB0000691	2	Palmitoyl sphingomyelin	HMDB0013410	1
D-Maltose	HMDB0000163	2	Parabanic Acid	HMDB0013415	1
Erythritol	HMDB0002994	2	PC(o-16:1(9Z)/14:1(9Z))	HMDB0013409	1
Methylamine	HMDB0000164	2	PC(o-16:1(9Z)/20:4(8Z,11Z,14Z,17Z))	HMDB0013433	1
N-Acetylneuraminic acid	HMDB0000230	2	PC(o-16:0/22:6(4Z,7Z,10Z,13Z,16Z,19Z))	HMDB0013437	1
Acetylglycine	HMDB0000532	2	PC(o-18:1(9Z)/22:0)	HMDB0013442	1
Nervonic acid	HMDB0002368	2	PC(o-18:2(9Z,12Z)/22:0)	HMDB0013438	1
Niacinamide	HMDB0001406	2	PC(o-20:0/20:4(8Z,11Z,14Z,17Z))	HMDB0013451	1
Caprylic acid	HMDB0000482	2	PC(o-18:2(9Z,12Z)/24:0)	HMDB0007920	1
L-Octanoylcarnitine	HMDB0000791	2	PC(o-22:1(13Z)/20:4(8Z,11Z,14Z,17Z))	HMDB0000564	1
p-Aminobenzoic acid	HMDB0001392	2	PC(14:1(9Z)/22:1(13Z))	HMDB0008166	1
PC(o-20:1(11Z)/20:4(8Z,11Z,14Z,17Z))	HMDB0013444	2	PC(16:0/16:0)	HMDB0008727	1
p-Cresol	HMDB0001858	2	PC(18:3(6Z,9Z,12Z)/16:0)	HMDB0013413	1
Pentadecanoic acid	HMDB0000826	2	PC(22:6(4Z,7Z,10Z,13Z,16Z,19Z)/18:0)	HMDB0011635	1
Phenol	HMDB0000228	2	PC(o-16:1(9Z)/18:2(9Z,12Z))	HMDB0009123	1
Phosphoric acid	HMDB0002142	2	p-Cresol sulfate	HMDB0009225	1
4-Hydroxybenzaldehyde	HMDB0011718	2	PE(18:3(6Z,9Z,12Z)/18:0)	HMDB0009682	1
Picolinic acid	HMDB0002243	2	PE(20:0/18:2(9Z,12Z))	HMDB0011342	1
Pipecolic acid	HMDB0000070	2	PE(22:6(4Z,7Z,10Z,13Z,16Z,19Z)/16:0)	HMDB0011352	1

Metabolite Name	HMDB ID	Frequency	Metabolite Name	HMDB ID	Frequency
Propionylcarnitine	HMDB0000824	2	PE(P-16:0/18:1(9Z))	HMDB0004586	1
S-Adenosylmethionine	HMDB0001185	2	PE(P-16:0/20:4(5Z,8Z,11Z,14Z))	HMDB0060015	1
L-Sorbose	HMDB0001266	2	Perillic acid	HMDB0001511	1
Spermidine	HMDB0001257	2	Phenol sulphate	HMDB0011185	1
Proline betaine	HMDB0004827	2	Phosphocreatine	HMDB0000500	1
D-Threitol	HMDB0004136	2	O-Phosphothreonine	HMDB0000020	1
Xanthosine	HMDB0000299	2	4-Hydroxybenzoic acid	HMDB0000857	1
Octadecenoylcarnitine	HMDB0094687	2	p-Hydroxyphenylacetic acid	HMDB0009923	1
D-Xylitol	HMDB0002917	2	Pimelic acid	HMDB0029010	1
Glyceric acid 1,3-biphosphate	HMDB0001270	1	PIP(16:0/18:0)	HMDB0012341	1
Terephthalic Acid	HMDB0002428	1	Prolyl-Alanine	HMDB0012378	1
cis-Vaccenic acid	HMDB0240219	1	PS(14:1(9Z)/14:0)	HMDB0000767	1
12a-Hydroxy-3-oxocholadienic acid	HMDB0000385	1	PS(18:0/18:0)	HMDB0000802	1
12-Ketodeoxycholic acid	HMDB0000328	1	Pseudouridine	HMDB0001545	1
12-Keto-leukotriene B4	HMDB0004234	1	Pterin	HMDB0001431	1
13'-Carboxy-alpha-tocopherol	HMDB0012555	1	Pyridoxal	HMDB0000239	1
13-L-Hydroperoxylinoleic acid	HMDB0003871	1	Pyridoxamine	HMDB0062558	1
13-OxoODE	HMDB0004668	1	Pyridoxine	HMDB0061890	1
13-Methylmyristic acid	HMDB0061707	1	Pyroglutamine	HMDB0000867	1
Myristoleic acid	HMDB0002000	1	Pyroglutamylglycine	HMDB0000621	1
17-Methylstearate	HMDB0061710	1	Ribonic acid	HMDB0004122	1
19(S)-HETE	HMDB0011136	1	D-Ribulose	HMDB0029045	1
1-Deoxy-D-xylulose 5-phosphate	HMDB0001213	1	Selenocystine	HMDB0000494	1
1-Hexadecanol	HMDB0003424	1	Serylmethionine	HMDB0002108	1
1-Methyladenosine	HMDB0003331	1	Stigmastanol	HMDB0001256	1

Metabolite Name	HMDB ID	Frequency	Metabolite Name	HMDB ID	Frequency
N-Methylhydantoin	HMDB0003646	1	Methylcysteine	HMDB0000269	1
N2,N2-Dimethylguanosine	HMDB0004824	1	Spermine	HMDB0000256	1
2,3-Diphosphoglyceric acid	HMDB0001294	1	Sphinganine	HMDB0034146	1
Eicosapentaenoic acid	HMDB0001999	1	Squalene	HMDB0006547	1
Erucic acid	HMDB0002068	1	Octadecanamide	HMDB0000912	1
Docosahexaenoic acid	HMDB0002183	1	Stearidonic acid	HMDB0000258	1
Hexacosanoic acid	HMDB0002356	1	Succinyladenosine	HMDB0000951	1
2-Acetyl-5-methylpyridine	HMDB0040222	1	Sucrose	HMDB0000036	1
Tromethamine	HMDB0240288	1	Taurochenodesoxycholic acid	HMDB0000896	1
2-Aminoisobutyric acid	HMDB0001906	1	Taurocholic acid	HMDB0002580	1
2-Dodecylbenzenesulfonic acid	HMDB0031031	1	Taurodeoxycholic acid	HMDB0004626	1
2-Hydroxyacetaminophen sulfate	HMDB0062547	1	Taurolithocholic acid 3-sulfate	HMDB0004041	1
2-Hydroxyestradiol	HMDB0000338	1	Tetrahydrogestrinone	HMDB0029057	1
Salicyluric acid	HMDB0000840	1	L-Allothreonine	HMDB0029070	1
2-Hydroxy-3-methylbutyric acid	HMDB0000407	1	Threoninyl-Aspartate	HMDB0000262	1
2-Hydroxystearic acid	HMDB0062549	1	Threoninyl-Serine	HMDB0000906	1
2-Keto-glutaramic acid	HMDB0001552	1	Thymine	HMDB0000958	1
Dmpec 2-methylpropanoate	HMDB0037640	1	Trimethylamine	HMDB0002163	1
Glyceryl 2-stearate	HMDB0011535	1	trans-Aconitic acid	HMDB0029083	1
2-Ketobutyric acid	HMDB0000005	1	Trihydroxycoprostanoic acid	HMDB0029103	1
Alpha-ketoisovaleric acid	HMDB0000019	1	Tryptophyl-Glycine	HMDB0001072	1
3,4,5-Trimethoxycinnamic acid	HMDB0002511	1	Tyrosyl-Glutamine	HMDB0001018	1
m-Aminobenzoic acid	HMDB0001891	1	Coenzyme Q10	HMDB0000288	1
3'-AMP	HMDB0003540	1	UDP-D-Xylose	HMDB0000286	1
3-Dehydrocarnitine	HMDB0012154	1	Uridine 5'-monophosphate	HMDB0000290	1

Metabolite Name	HMDB ID	Frequency	Metabolite Name	HMDB ID	Frequency
3-Hydroxyisovalerylcarnitine	HMDB0061189	1	Uridine diphosphate glucose	HMDB0000946	1
3-Hydroxy-L-proline	HMDB0002113	1	Uridine diphosphate-N-acetylglucosamine	HMDB0029123	1
3-Hydroxymethylglutaric acid	HMDB0000355	1	Ursodeoxycholic acid	HMDB0029129	1
1-O-Heptadecylglycerol	/	2	LPA(18:0)	/	1
Cholesterol derivative	/	2	LPC C20:4	/	1
Isocytosine	/	2	LPS(20:4)	/	1
Marganic acid	/	2	lysine minor	/	1
PC/GPC	/	2	LysoPE(0:0/20:4)	/	1
Propyl octadecanoate	/	2	Malonic acid, Bis TMS	/	1
Isoglutamine	/	2	Methioninamide	/	1
1,1,4,4-Tetramethyl-2,5-dimethylene-cyclohexane	/	1	Methyl palmitate (15 or 2)	/	1
10-Hydroxydecanoic acid	/	1	MG(0:0/18:3/0:0)	/	1
10-undecenoate (11:1n1)	/	1	Monoacyl glycerol	/	1
1-palmitoyl glycerophosphoethanolamine	/	1	Monostearin	/	1
2,21-Dimethyldecane	/	1	MUFA	/	1
2,3-epoxymenaquinone	/	1	Myristamide	/	1
21-Methylpropanoate	/	1	N2,N2-dimethylguanine	/	1
2-hydroxypalmitate	/	1	N-acetyl signal of glycoproteins	/	1
3-Cystein-S-YL-acetaminophen	/	1	O-acetyl glycoprotein	/	1
4- methyl octane	/	1	octenedioate	/	1
41-Ethyl-111-octyn1-31-ol	/	1	PC aa C24:0	/	1
5M(38:8)	/	1	PC aa C26:0	/	1
61-t1-Butyl-12,2,9,91-tetramethyl-13,51-	/	1	PC aa C40:1	/	1

Metabolite Name	HMDB ID	Frequency	Metabolite Name	HMDB ID	Frequency
decadien-171-yne					
6-t-Butyl-2,2,9,9-tetramethyl-3,5-decadien-7-yne	/	1	PC aa C40:2	/	1
9-HpODE	/	1	PC aa C40:3	/	1
Acylcarnitine (C13:0)	/	1	PC aa C42:2	/	1
Acylcarnitine (C9:0)	/	1	PC aa C42:4	/	1
Acylcarnitine (C9-OH)	/	1	PC aa C42:5	/	1
Andro steroid monosulfate 2	/	1	PC ae C30:1	/	1
beta-alanine(minor)	/	1	PC ae C36:1	/	1
beta-Glu-cys	/	1	PC ae C38:1	/	1
C10:1	/	1	PC ae C38:2	/	1
C11H20O4N2	/	1	PC ae C38:3	/	1
C16:1-OH	/	1	PC ae C40:3	/	1
C18:2	/	1	PC ae C42:1	/	1
carnitine (18:1)	/	1	PC ae C42:3	/	1
Cer(36:1)	/	1	PC ae C42:4	/	1
Cer(44:5)	/	1	PC ae C44:3	/	1
ChoCC	/	1	PC(18:1/16:0)	/	1
choline-containing compounds	/	1	PC(32:2)	/	1
cisovalerylcarnitine	/	1	PC(34:3)	/	1
Cis-Urocanate	/	1	PC(34:4)	/	1
CL(18:1/18:1/20:4/18:2)	/	1	PC(40:5)	/	1
CoA-[4'-phosphopantetheinel]	/	1	PC(O-35:4)	/	1
Conjugated linoleate-18-2N7	/	1	PE(22:6/p-18:1)	/	1
Creatinine enol (enolic form)	/	1	PE(32:1)	/	1

Metabolite Name	HMDB ID	Frequency	Metabolite Name	HMDB ID	Frequency
Cyclo(phe-phe)	/	1	PE(34:1)	/	1
Cyclooctylmethanol	/	1	PE(36:4)	/	1
Cystamine	/	1	PE(36:5)	/	1
DG((40:7)	/	1	PE(38:2)	/	1
DG(37:1)	/	1	PE(38:6)	/	1
DG(38:6)	/	1	PE(40:6)	/	1
Dihomo-linoleate (20:2n6)	/	1	PE(40:7)	/	1
Dihomo-linolenate	/	1	PE(42:1)	/	1
Dodecane 31-hydroxy-12,4,41-trimethylpentyl	/	1	PE(o-18:1/20:4)	/	1
Eicosenoate (20:1n9 or 11)	/	1	PE(O-34:1)	/	1
erythrotetrofuranose	/	1	PE(O-34:2)	/	1
Ethylaniline	/	1	PE(O-34:3)	/	1
Ethylene glycol, di-TMS	/	1	PE(O-36:2)	/	1
F1,6DP	/	1	PE(O-36:3)	/	1
FFA(20:1)	/	1	PE(O-36:5)	/	1
FFA(20:2)	/	1	PE(O-38:3)	/	1
FFA(22:4)	/	1	PE(O-38:5)	/	1
FFA(ZZ:2)	/	1	PE(O-38:6)	/	1
galactonate gamma-lactone	/	1	PE(O-40:3)	/	1
GalCer(33:2)	/	1	PE(p-18:0/18:2)	/	1
GalCer(34:1)	/	1	PEIO-36:4)	/	1
GalCer(36:4)	/	1	Peptide NHs	/	1
GalCer(41:2)	/	1	pG(34:0)	/	1
GalCer(42:2)	/	1	PGP(16:1/22:6)	/	1

Metabolite Name	HMDB ID	Frequency	Metabolite Name	HMDB ID	Frequency
GalCer(42:3)	/	1	Phenyl methylcarbamate	/	1
Ganglioside GM1(d18:0/12:0)	/	1	polyols	/	1
Glanglioside GM2(d18:1/18:1)	/	1	Prolylleucine	/	1
GluCer(42:3)	/	1	PS(37:2)	/	1
Glucosamine_2	/	1	Pyro-glutamyl-glycine	/	1
Glucuronate_1	/	1	S7P	/	1
Glu-Gly/Gly-Glu	/	1	SM C22:3	/	1
Glycerol 1-(9-octadecenoate)	/	1	SM(32:2)	/	1
Glycerol phosphate	/	1	SM(37:1)	/	1
Glycolate-hydroxyacetate	/	1	SM(42:2)	/	1
Gly-Thr/Ser-Ala	/	1	SM(44:2)	/	1
Histidyl-alanine	/	1	Stearic acid, trimethylsilyl ester	/	1
Histidyl-glycine	/	1	TG(52:1)	/	1
Histidyl-phenylalanine	/	1	TG(52:2)	/	1
Hydroxyproline/Aminolevulinate	/	1	TG(56:6)	/	1
inositol stereoisomer	/	1	Trans-121-Dodecen-111-ol	/	1
LacCer(35:1)	/	1	Ursodesoxycholic acid	/	1
LacCer(38:1)	/	1	Lipid	/	8
LacCer(40:1)	/	1	n3	/	1
LacCer(40:2)	/	1	n6	/	1
LacCer(40:4)	/	1	PUFA	/	1
LacCer(42:4)	/	1	ToFA	/	1
LPA(16:0)	/	1	UFA	/	1
			VLDL	/	1

S.Table 6. Altered pathways reported in previous metabolomics study of colorectal cancer

Pathway Name	Frequency[ref]	Pathway Name	Frequency[ref]	Pathway Name	Frequency[ref]
TCA cycle	8[5,15,23,39,42,43,51,61]	Alanine and aspartate metabolism	2[43,61]	Histamine metabolism	1[51]
Glycine, serine and threonine metabolism	6[10,18,26,39,43,61]	Fatty acid biosynthesis	2[42,43]	Fatty acid oxidation	1[50]
Glutathione metabolism	5[15,18,26,39,51]	Tyrosine metabolism	2[39,42]	Glutamine metabolism	1[50]
Pyrimidine metabolism	5[10,26,42,43,61]	Carbohydrate metabolism	2[39,61]	Protein metabolism	1[50]
Fatty acid metabolism	4[15,23,26,39]	Nitrogen metabolism	2[10,26]	Butanoate metabolism	1[26]
Purine metabolism	4[18,26,43,61]	Creatinine metabolism	1[15]	Lysine degradation	1[26]
Arginine and proline metabolism	4[10,18,39,43]	Gluconeogenesis	1[15]	Methane metabolism	1[26]
Glycolysis	4[5,23,42,43]	Glyoxylate Dicarboxylate metabolism	1[15]	Porphyrin and chlorophyll metabolism	1[26]
Gut flora metabolism	4[15,39,42,,51]	Urea metabolism	1[15]	Steroid hormone biosynthesis	1[26]
Tryptophan metabolism	4[39,42,43,51]	Disulfiram Pathway	1[15]	Synthesis and degradation of ketone bodies	1[26]
Urea cycle	4[23,39,42,61]	Methionine metabolism	1[15]	Taurine and hypotaurine metabolism	1[26]
Aminoacyl-tRNA biosynthesis	3[10,18,26]	sphingolipid metabolism	1[12]	Vitamin B6 metabolism	1[26]
Fructose and mannose metabolism	3[10,26,43]	Ascorbate and aldarate metabolism	1[10]	ABC transport	1[43]
Cysteine and methionine metabolism	3[18, 26,39]	Glycine serine and threonine metabolism	1[10]	Eicosanoid biosynthesis	1[43]
beta-Alanine metabolism	3[10,18,43]	Nicotinate and nicotinamide metabolism	1[10]	Glyoxylate and dicarboxylate metabolism	1[43]
Choline metabolism	3[5,23,39]	Lysine biosynthesis	1[10]	Methionine biosynthesis	1[43]
Glutamate metabolism	3[15,51,61]	Pyrimidine metabolism	1[10]	Pentose and glucuronate interconversions	1[43]
Phenylalanine metabolism	3[15,39,61]	Starch and sucrose metabolism	1[10]	Phenylalanine biosynthesis	1[43]
Amino acid metabolism	2[15,23]	Pantothenate and CoA biosynthesis	1[10]	Proline biosynthesis	1[43]

Glutaminolysis	2[15,23]	β-alanine metabolism	1[10]	Steroid biosynthesis	1[43]
Amino sugar and nucleotide sugar metabolism	2[10,26]	Nucleotide	1[5]	Bile acid metabolism	1[43]
Cyanoamino acid metabolism	2[10,26]	lipids pathways	1[5]	Glucose metabolism	1[43]
Primary bile acid biosynthesis	2[26,43]	Ketoplasia	1[23]	L-Isoleucine metabolism	1[43]
Pentose phosphate pathway	2[10,39]	urea cycle (arginine)	1[23]	Nucleic acid metabolism	1[39]
Valine, leucine and isoleucine degradation	2[26,43]	serine synthesis (serine and glycine)	1[23]	Oxidative phosphorylation	1[39]
Glycerolipid metabolism	2[12,43]	Endogenous methanethiol metabolism	1[15]	Phospholipid metabolism	1[39]
Glycerophospholipid metabolism	2[12,43]	tyrosine biosynthesis	1[21]	Proline metabolism	1[39]
Galactose metabolism	2[10,43]	phenylalanine aerobic degradation	1[21]	Purine nucleotide synthetic	1[39]
Inositol phosphate metabolism	2[10,43]	L-alanine, D-glucose transport	1[21]	Thiamine metabolism	1[42]
Histidine metabolism	2[10,43]	glycine biosynthesis	1[21]	Free fatty acid metabolism	1[61]
Alanine,aspartate and glutamate metabolism	2[18,39]	threonine degradation	1[21]	Polyamine metabolism	1[42]
Lipid metabolism	2[15,61]	Aspartic acid metabolism	1[51]	Degradation of actin and myosin	1[51]

Note, the reference numbers are the same as in Supplementary Table 12.

S.Table 7. Pathway enrichment analysis of the 635 metabolites reported in 62 published metabolomics studies of colorectal cancer

Pathways	Total	Hits	*P*-value	FDR	Impact
Glycine, serine and threonine metabolism	48	19	2.09E-06	0.00016738	0.60596
Alanine, aspartate and glutamate metabolism	24	12	9.77E-06	0.0003908	0.88509
Arginine and proline metabolism	77	23	4.39E-05	0.0011699	0.68002
Nitrogen metabolism	39	14	0.00017002	0.0034004	0.0083
Glycerophospholipid metabolism	39	13	0.00066854	0.0099043	0.6366
Aminoacyl-tRNA biosynthesis	75	20	0.00074282	0.0099043	0.22536
Citrate cycle (TCA cycle)	20	8	0.002011	0.02011	0.42756
Taurine and hypotaurine metabolism	20	8	0.002011	0.02011	0.46583
Pyrimidine metabolism	60	16	0.0025049	0.022266	0.38956
Glycolysis or Gluconeogenesis	31	10	0.0036894	0.029515	0.16096
beta-Alanine metabolism	28	9	0.0059778	0.03995	0.33438
Glutathione metabolism	38	11	0.0059924	0.03995	0.43265
Cysteine and methionine metabolism	56	14	0.0085425	0.052012	0.68918
Butanoate metabolism	40	11	0.009102	0.052012	0.2262
Valine, leucine and isoleucine biosynthesis	27	8	0.015878	0.07939	0.19132
Pantothenate and CoA biosynthesis	27	8	0.015878	0.07939	0.32666
Glyoxylate and dicarboxylate metabolism	50	12	0.019957	0.088696	0.39757
Starch and sucrose metabolism	50	12	0.019957	0.088696	0.45217
Phenylalanine metabolism	45	11	0.02227	0.093769	0.25214
Propanoate metabolism	35	9	0.027276	0.1091	0.15844
Synthesis and degradation of ketone bodies	6	3	0.030577	0.11222	0.7
Pentose and glucuronate interconversions	53	12	0.030861	0.11222	0.15587
Linoleic acid metabolism	15	5	0.033124	0.11521	0.65625

S.Table 8. Diagnostic efficiency of metabolites screened out from the two-stage case-control study in a population from China

NO.	HMDB ID	Compounds	VIP		AUC		*P*-value	
			Training set	Validation set	Training set	Validation set	Training set	Validation set
1	HMDB0059977	4-Hydroxy-5-(dihydroxyphenyl)-valeric acid-O-methyl-O-sulphate	4.218	5.043	0.769	0.729	<0.0001	<0.0000
2	HMDB0001155	Diadenosine triphosphate	2.263	3.812	0.756	0.806	<0.0001	<0.0001
3	HMDB0000071	Deoxyinosine	2.18	2.56	0.632	0.661	0.0130	0.0030
4	HMDB0000195	Inosine	3.244	3.222	0.742	0.663	<0.0001	<0.0001
5	HMDB0003537	2'-Deoxyinosine triphosphate	2.54	3.012	0.867	0.814	<0.0001	<0.0001
6	HMDB0059921	Trimetaphosphoric acid	2.239	1.851	0.764	0.695	<0.0001	<0.0001
7	HMDB0000143	D-Galactose	5.569	11.11	0.781	0.796	<0.0001	<0.0001
8	HMDB0028776	Cysteinyl-Hydroxyproline	3.786	6.241	0.774	0.766	<0.0001	<0.0001
9	HMDB0001123	2-Aminobenzoic acid	1.772	1.69	0.836	0.694	<0.0001	<0.0001
10	HMDB0001068	D-Sedoheptulose 7-phosphate	2.295	4.497	0.709	0.701	<0.0001	<0.0001
11	HMDB0003540	3'-AMP	1.512	2.269	0.644	0.638	<0.0001	<0.0001
12	HMDB0000289	Uric acid	6.061	3.092	0.776	0.705	<0.0001	0.0010
13	HMDB0001031	Deoxyribose 5-phosphate	1.705	1.745	0.735	0.654	<0.0001	<0.0001
14	HMDB0001308	5'-Phosphoribosyl-N-formylglycinamide	12.418	4.531	0.832	0.718	<0.0001	<0.0001
15	HMDB0028754	Aspartyl-Hydroxyproline	3.365	1.98	0.811	0.668	<0.0001	<0.0001
16	HMDB0029433	L-2-Amino-4-methylenepentanedioic acid	7.936	6.23	0.815	0.71	<0.0001	<0.0001
17	HMDB0000725	4-Hydroxyproline	4.353	2.333	0.816	0.592	<0.0001	0.0150
18	HMDB0000094	Citric acid	10.6	9.518	0.838	0.733	<0.0001	<0.0001
19	HMDB0000157	Hypoxanthine	1.836	1.851	0.6	0.561	0.0010	0.0020
20	HMDB0000267	Pyroglutamic acid	2.554	3.237	0.817	0.714	<0.0001	<0.0001

NO.	HMDB ID	Compounds	VIP		AUC		*P*-value	
			Training set	Validation set	Training set	Validation set	Training set	Validation set
21	HMDB0000794	N-Acetyl-9-O-acetylneuraminic acid	1.621	1.969	0.836	0.637	<0.0001	<0.0001
22	HMDB0003335	IDP	1.735	2.918	0.775	0.688	<0.0001	<0.0001
23	HMDB0001107	7-Methylguanosine	2.018	1.767	0.741	0.61	<0.0001	0.0030
24	HMDB0000497	5,6-Dihydrouridine	19.121	7.25	0.847	0.652	<0.0001	0.0030
25	HMDB0001209	Allantoic acid	7.161	5.256	0.791	0.683	<0.0001	<0.0001
26	HMDB0000734	Indoleacrylic acid	4.225	6.334	0.849	0.715	<0.0001	<0.0001
27	HMDB0001517	AICAR	1.651	5.708	0.725	0.708	<0.0001	<0.0001
28	HMDB0000193	Isocitric acid	9.132	13.624	0.84	0.699	<0.0001	<0.0001
29	HMDB0000134	Fumaric acid	1.941	1.631	0.816	0.592	<0.0001	<0.0001
30	HMDB0001264	Dehydroascorbic acid	2.294	2.826	0.857	0.685	<0.0001	<0.0001
31	HMDB0001498	Inositol 1,4,5-trisphosphate	1.912	3.017	0.825	0.705	<0.0001	<0.0001
32	HMDB0000159	L-Phenylalanine	1.534	2.936	0.682	0.668	0.0080	<0.0001
33	HMDB0001406	Niacinamide	1.787	2.643	0.736	0.712	<0.0001	<0.0001
34	HMDB0000929	L-Tryptophan	2.565	7.846	0.764	0.768	<0.0001	<0.0001
35	HMDB0002249	6-Methyltetrahydropterin	3.219	4.605	0.851	0.723	<0.0001	<0.0001
36	HMDB0006344	Alpha-N-Phenylacetyl-L-glutamine	3.972	8.111	0.936	0.928	<0.0001	<0.0001
37	HMDB0000682	Indoxyl sulfate	5.647	3.113	0.964	0.72	<0.0001	<0.0001
38	HMDB0000714	Hippuric acid	1.674	4.101	0.832	0.833	<0.0001	<0.0001
39	HMDB0001843	N-Acryloylglycine	2.012	2.834	0.917	0.845	<0.0001	<0.0001
40	HMDB0028891	Histidinyl-Methionine	1.61	1.879	0.861	0.733	<0.0001	<0.0001
41	HMDB0002048	m-Cresol	5.852	2.196	0.954	0.756	<0.0001	<0.0001
42	HMDB0011635	p-Cresol sulfate	13.005	5.739	0.957	0.768	<0.0001	<0.0001
43	HMDB0007900	PC(14:1(9Z)/14:1(9Z))	3.808	2.488	0.874	0.501	<0.0001	0.0360
44	HMDB0000001	1-Methylhistidine	1.668	2.162	0.8	0.662	<0.0001	<0.0001
45	HMDB0002184	L-Threo-3-Phenylserine	17.96	18.121	0.889	0.803	<0.0001	<0.0001

NO.	HMDB ID	Compounds	VIP		AUC		*P*-value	
			Training set	Validation set	Training set	Validation set	Training set	Validation set
46	HMDB0002096	3-Indolebutyric acid	1.795	4.172	0.773	0.572	<0.0001	0.0020
47	HMDB0012144	2-Amino-4-oxo-6-(1',2',3'-trihydroxypropyl)-diquinoid-7,8-dihydroxypterin	3.756	4.552	0.79	0.699	<0.0001	<0.0001
48	HMDB0000707	4-Hydroxyphenylpyruvic acid	2.066	3.426	0.809	0.682	<0.0001	<0.0001
49	HMDB0001351	Deoxyribose 1-phosphate	1.582	1.655	0.767	0.627	<0.0001	<0.0001
50	HMDB0002596	Deoxycholic acid 3-glucuronide	2.536	1.68	0.765	0.568	0.0010	0.0160
51	HMDB0004710	9,10,13-TriHOME	4.183	5.439	0.875	0.734	<0.0001	<0.0001
52	HMDB0000637	Glycochenodeoxycholate	2.109	5.561	0.651	0.686	0.0080	<0.0001
53	HMDB0061698	1-Stearoylglycerophosphoserine	1.534	1.761	0.756	0.663	<0.0001	<0.0001
54	HMDB0000394	3-Hydroxytetradecanedioic acid	1.893	2.69	0.712	0.615	0.0020	<0.0001
55	HMDB0000631	Deoxycholic acid glycine conjugate	4.029	10.138	0.641	0.725	0.0270	<0.0001
56	HMDB0000951	Taurochenodesoxycholic acid	1.57	2.053	0.678	0.63	<0.0001	<0.0001
57	HMDB0000626	Deoxycholic acid	1.571	5.377	0.856	0.703	0.0070	<0.0001
58	HMDB0000946	Ursodeoxycholic acid	2.385	4.173	0.845	0.916	<0.0001	<0.0001
59	HMDB0001085	Leukotriene B4	3.31	5.107	0.71	0.629	<0.0001	<0.0001
60	HMDB0010727	3-Oxododecanoic acid	1.69	1.562	0.847	0.69	<0.0001	<0.0001
61	HMDB0004257	3Z-dodecenoyl-CoA	1.668	1.587	0.673	0.579	<0.0001	0.0080
62	HMDB0011503	LysoPE(16:0/0:0)	2.731	4.059	0.639	0.63	<0.0001	<0.0001
63	HMDB0011495	LysoPE(0:0/22:5(7Z,10Z,13Z,16Z,19Z))	3.705	6.906	0.789	0.769	<0.0001	<0.0001
64	HMDB0011499	LysoPE(0:0/24:6(6Z,9Z,12Z,15Z,18Z,21Z))	1.827	2.908	0.803	0.806	<0.0001	<0.0001
65	HMDB0011473	LysoPE(0:0/16:0)	9.529	13.453	0.936	0.879	<0.0001	<0.0001
66	HMDB0000686	Isoursodeoxycholic acid	3.877	11.769	0.752	0.947	<0.0001	<0.0001
67	HMDB0000672	Hexadecanedioic acid	1.926	3.026	0.769	0.64	<0.0001	<0.0001

NO.	HMDB ID	Compounds	VIP		AUC		*P*-value	
			Training set	Validation set	Training set	Validation set	Training set	Validation set
68	HMDB0000384	3a,7a-Dihydroxycholanoic acid	5.532	2.94	0.913	0.923	<0.0001	<0.0001
69	HMDB0000391	7-Ketodeoxycholic acid	1.795	3.141	0.845	0.813	<0.0001	<0.0001
70	HMDB0011506	LysoPE(18:1(9Z)/0:0)	3.137	5.516	0.644	0.663	0.0030	<0.0001
71	HMDB0011523	LysoPE(22:4(7Z,10Z,13Z,16Z)/0:0)	4.354	8.451	0.716	0.737	<0.0001	<0.0001
72	HMDB0010734	(R)-3-Hydroxy-hexadecanoic acid	1.889	2.131	0.796	0.653	<0.0001	<0.0001
73	HMDB0010379	LysoPC(14:0)	12.464	7.829	0.901	0.885	<0.0001	<0.0001
74	HMDB0010387	LysoPC(18:3(6Z,9Z,12Z))	4.964	2.124	0.862	0.763	<0.0001	<0.0001
75	HMDB0011494	LysoPE(0:0/22:5(4Z,7Z,10Z,13Z,16Z))	2.852	4.036	0.885	0.819	<0.0001	<0.0001
76	HMDB0011493	LysoPE(0:0/22:4(7Z,10Z,13Z,16Z))	5.319	5.052	0.753	0.809	<0.0001	<0.0001
77	HMDB0003871	13-L-Hydroperoxylinoleic acid	2.899	2.662	0.747	0.558	<0.0001	0.0340
78	HMDB0010730	3-Oxotetradecanoic acid	1.756	1.52	0.856	0.691	<0.0001	<0.0001
79	HMDB0013333	3-Hydroxy-9-hexadecenoylcarnitine	1.606	1.665	0.695	0.617	0.0010	<0.0001
80	HMDB0004667	13S-hydroxyoctadecadienoic acid	8.337	14.845	0.811	0.693	<0.0001	<0.0001
81	HMDB0011529	LysoPE(24:6(6Z,9Z,12Z,15Z,18Z,21Z)/0:0)	2.029	3.284	0.975	0.917	<0.0001	<0.0001
82	HMDB0010223	9-HODE	2.86	5.504	0.814	0.672	<0.0001	<0.0001
83	HMDB0010397	LysoPC(20:5(5Z,8Z,11Z,14Z,17Z))	5.91	9.079	0.807	0.791	<0.0001	<0.0001
84	HMDB0004668	13-OxoODE	2.602	1.841	0.879	0.677	<0.0001	<0.0001
85	HMDB0011475	LysoPE(0:0/18:1(11Z))	2.91	2.789	0.71	0.671	<0.0001	0.0010
86	HMDB0010404	LysoPC(22:6(4Z,7Z,10Z,13Z,16Z,19Z))	5.036	7.449	0.802	0.761	<0.0001	<0.0001
87	HMDB0000896	Taurodeoxycholic acid	1.533	1.721	0.732	0.604	<0.0001	<0.0001
88	HMDB0000138	Glycocholic acid	1.514	4.136	0.641	0.659	0.0010	<0.0001
89	HMDB0006319	Alpha-linolenyl carnitine	1.806	4.451	0.614	0.638	0.0260	<0.0001
90	HMDB0010388	LysoPC(18:3(9Z,12Z,15Z))	7.323	9.832	0.874	0.848	<0.0001	<0.0001
91	HMDB0013331	3, 5-Tetradecadiencarnitine	6.461	4.822	0.858	0.717	<0.0001	<0.0001

NO.	HMDB ID	Compounds	VIP		AUC		*P*-value	
			Training set	Validation set	Training set	Validation set	Training set	Validation set
92	HMDB0010382	LysoPC(16:0)	7.302	13.179	0.927	0.894	<0.0001	<0.0001
93	HMDB0010393	LysoPC(20:3(5Z,8Z,11Z))	3.072	4.29	0.913	0.812	<0.0001	<0.0001
94	HMDB0011522	LysoPE(22:2(13Z,16Z)/0:0)	4.785	8.177	0.923	0.874	<0.0001	<0.0001
95	HMDB0010210	15-KETE	2.026	2.398	0.8	0.662	<0.0001	<0.0001
96	HMDB0002815	LysoPC(18:1(9Z))	1.56	2.89	0.881	0.854	<0.0001	<0.0001
97	HMDB0002014	cis-5-Tetradecenoylcarnitine	5.721	5.247	0.86	0.727	<0.0001	<0.0001
98	HMDB0008832	PE(14:0/18:4(6Z,9Z,12Z,15Z))	2.256	2.758	0.783	0.637	<0.0001	<0.0001
99	HMDB0010403	LysoPC(22:5(7Z,10Z,13Z,16Z,19Z))	6.283	9.683	0.909	0.783	<0.0001	<0.0001
100	HMDB0011520	LysoPE(22:0/0:0)	2.172	5.979	0.746	0.726	0.0020	<0.0001
101	HMDB0013334	9,12-Hexadecadienoylcarnitine	3.626	2.76	0.857	0.713	<0.0001	<0.0001
102	HMDB0002685	Prostaglandin F1a	3.311	4.109	0.844	0.733	<0.0001	<0.0001
103	HMDB0011130	LysoPE(18:0/0:0)	4.161	3.248	0.728	0.655	<0.0001	0.0010
104	HMDB0000036	Taurocholic acid	1.968	2.432	0.833	0.780	<0.0001	<0.0001
105	HMDB0010383	LysoPC(16:1(9Z))	3.549	6.158	0.969	0.937	<0.0001	<0.0001
106	HMDB0010402	LysoPC(22:5(4Z,7Z,10Z,13Z,16Z))	5.091	6.67	0.888	0.806	<0.0001	<0.0001
107	HMDB0006317	trans-Hexadec-2-enoyl carnitine	3.123	1.613	0.812	0.615	<0.0001	0.0090
108	HMDB0010386	LysoPC(18:2(9Z,12Z))	7.201	7.008	0.744	0.653	<0.0001	<0.0001
109	HMDB0010395	LysoPC(20:4(5Z,8Z,11Z,14Z))	6.348	4.22	0.829	0.604	<0.0001	<0.0001
110	HMDB0010381	LysoPC(15:0)	8.648	10.106	0.919	0.858	<0.0001	<0.0001
111	HMDB0010396	LysoPC(20:4(8Z,11Z,14Z,17Z))	5.381	5.323	0.698	0.662	<0.0001	<0.0001
112	HMDB0011491	LysoPE(0:0/22:1(13Z))	2.396	5.752	0.88	0.845	<0.0001	<0.0001
113	HMDB0011482	LysoPE(0:0/20:1(11Z))	4.657	5.502	0.947	0.849	<0.0001	<0.0001
114	HMDB0000331	3a,7b,12a-Trihydroxyoxocholanyl-Glycine	2.778	3.983	0.703	0.721	<0.0001	<0.0001
115	HMDB0010394	LysoPC(20:3(8Z,11Z,14Z))	12.045	13.707	0.914	0.816	<0.0001	<0.0001

NO.	HMDB ID	Compounds	VIP		AUC		*P*-value	
			Training set	Validation set	Training set	Validation set	Training set	Validation set
116	HMDB0034227	alpha-Tocopherol acetate	1.955	3.429	0.755	0.691	<0.0001	<0.0001
117	HMDB0003073	Gamma-Linolenic acid	4.693	6.532	0.754	0.669	<0.0001	<0.0001
118	HMDB0006547	Stearidonic acid	1.982	1.959	0.645	0.6	<0.0001	<0.0001
119	HMDB0010401	LysoPC(22:4(7Z,10Z,13Z,16Z))	2.998	4.203	0.92	0.803	<0.0001	<0.0001
120	HMDB0000518	Chenodeoxycholic acid	1.757	3.328	0.673	0.634	<0.0001	<0.0001
121	HMDB0010392	LysoPC(20:2(11Z,14Z))	7.414	9.142	0.917	0.837	<0.0001	<0.0001
122	HMDB0013122	LysoPC(P-18:0)	1.629	3.217	0.774	0.714	<0.0001	<0.0001
123	HMDB0010733	3-Oxohexadecanoic acid	2.077	2.038	0.688	0.605	<0.0001	<0.0001
124	HMDB0012108	LysoPC(17:0)	11.342	12.608	0.931	0.822	<0.0001	<0.0001
125	HMDB0002183	Docosahexaenoic acid	2.69	3.093	0.701	0.6	<0.0001	0.0110
126	HMDB0000664	Isohyodeoxycholic acid	1.747	3.933	0.695	0.705	<0.0001	<0.0001
127	HMDB0011128	LysoPC(0:0/18:0)	9.879	13.001	0.873	0.796	<0.0001	<0.0001
128	HMDB0003229	Palmitoleic acid	2.747	3.925	0.715	0.634	<0.0001	<0.0001
129	HMDB0010408	LysoPC(P-18:1(9Z))	6.017	6.247	0.858	0.612	<0.0001	0.0030
130	HMDB0010384	LysoPC(18:0)	10.739	3.331	0.832	0.822	<0.0001	<0.0001
131	HMDB0010391	LysoPC(20:1(11Z))	4.795	5.87	0.828	0.778	<0.0001	<0.0001
132	HMDB0008242	PC(18:4(6Z,9Z,12Z,15Z)/20:1(11Z))	3.352	2.525	0.778	0.608	<0.0001	<0.0001
133	HMDB0006528	Docosapentaenoic acid	2.045	3.198	0.725	0.691	<0.0001	<0.0001
134	HMDB0000673	Linoleic acid	12.741	11.724	0.794	0.688	<0.0001	<0.0001
135	HMDB0011490	LysoPE(0:0/22:0)	3.731	4.683	0.881	0.844	<0.0001	<0.0001
136	HMDB0008885	PE(14:1(9Z)/P-18:1(11Z))	3.209	2.664	0.757	0.618	<0.0001	<0.0001
137	HMDB0009211	PE(18:4(6Z,9Z,12Z,15Z)/24:0)	2.206	1.776	0.594	0.559	0.0020	0.0230
138	HMDB0010390	LysoPC(20:0)	2.65	3.397	0.768	0.75	<0.0001	<0.0001
139	HMDB0000220	Palmitic acid	6.065	11.471	0.746	0.674	<0.0001	<0.0001
140	HMDB0002231	Eicosenoic acid	3.44	4.5	1	0.868	<0.0001	<0.0001

NO.	HMDB ID	Compounds	VIP		AUC		*P*-value	
			Training set	Validation set	Training set	Validation set	Training set	Validation set
141	HMDB0007955	PC(15:0/22:4(7Z,10Z,13Z,16Z))	4.538	4.262	0.758	0.6	<0.0001	0.0020
142	HMDB0010405	LysoPC(24:0)	1.504	1.697	0.723	0.641	<0.0001	0.0010
143	HMDB0010167	PS(18:0/22:6(4Z,7Z,10Z,13Z,16Z,19Z))	4.121	3.303	0.811	0.642	<0.0001	<0.0001
144	HMDB0000207	Oleic acid	12.594	12.9	0.819	0.688	<0.0001	<0.0001
145	HMDB0008330	PC(20:2(11Z,14Z)/15:0)	3.886	1.968	0.761	0.601	<0.0001	0.0070
146	HMDB0010165	PS(18:0/20:4(8Z,11Z,14Z,17Z))	7.22	6.172	0.846	0.668	<0.0001	<0.0001
147	HMDB0010163	PS(18:0/18:1(9Z))	7.991	3.808	0.783	0.608	<0.0001	0.0030

S.Table 9. Diagnostic efficiency of the eleven metabolites in a population from China

HMDB ID	Compounds	AUC 95%CI		Sensitivity		Specificity	
		Training set	Validation set	Training set	Validation set	Training set	Validation set
HMDB0000384	3a,7a-Dihydroxycholanoic acid	0.913 (0.855, 0.970)	0.923 (0.904, 0.970)	0.897	0.913	0.939	0.915
HMDB0006344	Alpha-N-Phenylacetyl-L-glutamine	0.936 (0.887, 0.985)	0.928 (0.885, 0.961)	0.926	0.929	0.909	0.858
HMDB0002815	LysoPC(18:1(9Z))	0.881 (0.821, 0.940)	0.854 (0.805, 0.903)	0.882	0.819	0.758	0.802
HMDB0010382	LysoPC(16:0)	0.927 (0.884, 0.971)	0.885 (0.841, 0.928)	0.897	0.827	0.848	0.811
HMDB0010383	LysoPC(16:1(9Z))	0.969 (0.944, 0.994)	0.937 (0.904, 0.970)	0.926	0.866	0.924	0.915
HMDB0011473	LysoPE(0:0/16:0)	0.936 (0.899, 0.974)	0.879 (0.835, 0.923)	0.809	0.835	0.924	0.774
HMDB0011522	LysoPE(22:2(13Z,16Z)/0:0)	0.923 (0.874, 0.971)	0.874 (0.827, 0.920)	0.926	0.803	0.864	0.849
HMDB0011529	LysoPE(24:6(6Z,9Z,12Z,15Z,18Z,21Z)/0:0)	0.975 (0.951, 0.999)	0.917 (0.880, 0.954)	1.000	0.874	0.879	0.83
HMDB0010379	LysoPC(14:0)	0.924 (0.878, 0.970)	0.876 (0.829, 0.923)	0.809	0.843	0.909	0.811
HMDB0002231	Eicosenoic acid	1.000 (1.000, 1.000)	0.868 (0.820, 0.917)	1.000	0.752	0.985	0.99
HMDB0010381	LysoPC(15:0)	0.919 (0.871, 0.966)	0.858 (0.808, 0.908)	0.868	0.791	0.864	0.837

S.Table 10. Variables in the equation of binary logistic regression of eleven metabolites

	HMDB ID	B	S.E.	Wald	df	Sig.	Exp(B)
Step 1	HMDB0000384	0.704	0.235	8.985	1	0.003	2.021
	HMDB0006344	0.098	0.048	4.226	1	0.04	1.103
	HMDB0002815	-0.731	0.751	0.947	1	0.331	0.482
	HMDB0010382	-0.045	0.056	0.643	1	0.423	0.956
	HMDB0010383	0.514	0.272	3.578	1	0.059	1.672
	HMDB0011473	0.019	0.043	0.193	1	0.66	1.019
	HMDB0011522	0.085	0.081	1.098	1	0.295	1.088
	HMDB0011529	-0.105	0.601	0.03	1	0.862	0.901
	HMDB0010379	0.086	0.1	0.735	1	0.391	1.09
	HMDB0002231	1.261	0.415	9.224	1	0.002	3.528
	HMDB0010381	0.071	0.041	2.931	1	0.087	1.073
	Constant	-7.703	1.497	26.486	1	0	0
Step 2	HMDB0000384	0.696	0.229	9.238	1	0.002	2.006
	HMDB0006344	0.097	0.047	4.217	1	0.04	1.102
	HMDB0002815	-0.779	0.689	1.277	1	0.258	0.459
	HMDB0010382	-0.042	0.053	0.627	1	0.428	0.959
	HMDB0010383	0.501	0.26	3.728	1	0.054	1.651
	HMDB0011473	0.017	0.041	0.166	1	0.684	1.017
	HMDB0011522	0.085	0.08	1.118	1	0.29	1.088
	HMDB0010379	0.087	0.1	0.769	1	0.381	1.091
	HMDB0002231	1.266	0.418	9.157	1	0.002	3.545
	HMDB0010381	0.069	0.041	2.927	1	0.087	1.072
	Constant	-7.715	1.501	26.406	1	0	0
Step 3	HMDB0000384	0.681	0.219	9.63	1	0.002	1.975
	HMDB0006344	0.096	0.047	4.13	1	0.042	1.101
	HMDB0002815	-0.775	0.686	1.275	1	0.259	0.461
	HMDB0010382	-0.043	0.053	0.657	1	0.418	0.958
	HMDB0010383	0.551	0.231	5.679	1	0.017	1.735
	HMDB0011522	0.082	0.078	1.078	1	0.299	1.085
	HMDB0010379	0.111	0.082	1.85	1	0.174	1.118
	HMDB0002231	1.253	0.417	9.007	1	0.003	3.5
	HMDB0010381	0.069	0.04	2.952	1	0.086	1.072
	Constant	-7.673	1.483	26.767	1	0	0
Step 4	HMDB0000384	0.656	0.223	8.636	1	0.003	1.927
	HMDB0006344	0.094	0.046	4.151	1	0.042	1.099
	HMDB0002815	-0.962	0.618	2.423	1	0.12	0.382
	HMDB0010383	0.48	0.211	5.187	1	0.023	1.617
	HMDB0011522	0.068	0.074	0.825	1	0.364	1.07
	HMDB0010379	0.063	0.055	1.286	1	0.257	1.065
	HMDB0002231	1.229	0.407	9.097	1	0.003	3.418
	HMDB0010381	0.063	0.041	2.414	1	0.12	1.066

	Constant	-7.572	1.459	26.925	1	0	0.001
Step 5	HMDB0000384	0.631	0.219	8.342	1	0.004	1.88
	HMDB0006344	0.094	0.045	4.353	1	0.037	1.099
	HMDB0002815	-0.607	0.484	1.573	1	0.21	0.545
	HMDB0010383	0.546	0.2	7.465	1	0.006	1.726
	HMDB0010379	0.062	0.055	1.273	1	0.259	1.064
	HMDB0002231	1.162	0.383	9.226	1	0.002	3.196
	HMDB0010381	0.066	0.042	2.519	1	0.112	1.069
	Constant	-7.296	1.389	27.608	1	0	0.001
Step 6	HMDB0000384	0.654	0.223	8.593	1	0.003	1.924
	HMDB0006344	0.088	0.045	3.796	1	0.051	1.092
	HMDB0002815	-0.522	0.49	1.134	1	0.287	0.593
	HMDB0010383	0.628	0.196	10.291	1	0.001	1.873
	HMDB0002231	1.186	0.373	10.096	1	0.001	3.275
	HMDB0010381	0.066	0.04	2.787	1	0.095	1.069
	Constant	-6.787	1.25	29.5	1	0	0.001
Step 7	HMDB0000384	0.604	0.212	8.104	1	0.004	1.83
	HMDB0006344	0.073	0.041	3.241	1	0.072	1.076
	HMDB0010383	0.499	0.137	13.217	1	0	1.648
	HMDB0002231	1.236	0.391	10.001	1	0.002	3.443
	HMDB0010381	0.05	0.036	1.944	1	0.163	1.051
	Constant	-6.533	1.157	31.899	1	0	0.001
Step 8	HMDB0000384	0.594	0.201	8.733	1	0.003	1.812
	HMDB0006344	0.07	0.039	3.195	1	0.074	1.073
	HMDB0010383	0.556	0.135	16.946	1	0	1.744
	HMDB0002231	1.417	0.424	11.178	1	0.001	4.125
	Constant	-5.77	0.926	38.8	1	0	0.003

S.Table 11. Diagnostic efficiency of metabolites screened out from a tumor-adjacent non-malignant paired tissue metabolomics study

NO.	HMDB ID	Compounds	VIP	AUC	*P*-value
1	HMDB0000229	Nicotinamide ribotide	1.84	0.69	0.0017
2	HMDB0001254	Glucosamine 6-phosphate	1.54	0.71	0.0017
3	HMDB0003335	IDP	2.03	0.73	<0.0001
4	HMDB0028920	Isoleucyl-Valine	2.44	0.77	0.0004
5	HMDB0000095	Cytidine monophosphate	3.01	0.75	<0.0001
6	HMDB0060282	3'-UMP	3.41	0.61	0.0011
7	HMDB0001202	dCMP	2.24	0.71	<0.0001
8	HMDB0003464	4-Guanidinobutanoic acid	2.73	0.75	0.0008
9	HMDB0000288	Uridine 5'-monophosphate	1.80	0.74	<0.0001
10	HMDB0001555	Pyridoxamine 5'-phosphate	2.24	0.81	<0.0001
11	HMDB0029027	Prolyl-Threonine	1.72	0.68	0.0011
12	HMDB0000929	L-Tryptophan	1.91	0.73	<0.0001
13	HMDB0000212	N-Acetylgalactosamine	2.43	0.76	<0.0001
14	HMDB0002275	7,8-Dihydroneopterin	2.76	0.78	<0.0001
15	HMDB0001566	3-Methylguanine	2.22	0.74	0.0011
16	HMDB0001548	D-Ribose 5-phosphate	1.53	0.72	<0.0001
17	HMDB0000143	D-Galactose	2.47	0.82	<0.0001
18	HMDB0011162	L-beta-aspartyl-L-alanine	1.69	0.61	0.0017
19	HMDB0000034	Adenine	3.88	0.77	<0.0001
20	HMDB0000058	Cyclic AMP	4.00	0.82	<0.0001
21	HMDB0013287	Ne,Ne dimethyllysine	1.80	0.72	<0.0001
22	HMDB0000618	D-Ribulose 5-phosphate	5.65	0.75	<0.0001
23	HMDB0002273	4-Hydroxy-L-glutamic acid	2.15	0.80	<0.0001
24	HMDB0006268	N-Acetylneuraminate 9-phosphate	2.41	0.72	<0.0001
25	HMDB0001117	4-Phosphopantothenoylcysteine	5.55	0.74	<0.0001
26	HMDB0002393	N-Methyl-D-aspartic acid	4.04	0.74	0.0002
27	HMDB0006248	gamma-Glutamylalanine	2.92	0.66	0.0003
28	HMDB0001212	Hydantoin-5-propionic acid	2.27	0.70	0.0007
29	HMDB0002224	5 Methyldeoxycytidine	3.25	0.74	0.0002
30	HMDB0000052	Argininosuccinic acid	1.68	0.63	0.0022
31	HMDB0000991	DL-2-Aminooctanoic acid	12.50	0.86	<0.0001
32	HMDB0000905	Deoxyadenosine monophosphate	2.60	0.70	0.0001
33	HMDB0001185	S-Adenosylmethionine	1.61	0.64	0.0002
34	HMDB0003540	3'-AMP	3.04	0.73	0.0001
35	HMDB0001086	Uroporphyrinogen III	1.56	0.69	0.0019
36	HMDB0004824	N2，N2-Dimethylguanosine	2.75	0.67	0.0011
37	HMDB0000725	4-Hydroxyproline	1.78	0.73	0.0003
38	HMDB0003950	7-Methylinosine	1.78	0.77	<0.0001
39	HMDB0001238	N-Acetylserotonin	1.50	0.74	<0.0001
40	HMDB0029143	gamma-Glutamylarginine	2.82	0.81	<0.0001

41	HMDB0002048	m-Cresol	2.14	0.83	<0.0001
42	HMDB0000637	Glycochenodeoxycholate	1.80	0.78	<0.0001
43	HMDB0001107	7-Methylguanosine	4.78	0.87	<0.0001
44	HMDB0001855	5 Hydroxytryptophol	1.88	0.78	<0.0001
45	HMDB0001049	gamma-Glutamylcysteine	2.95	0.69	0.0002
46	HMDB0002497	Glycochenodeoxycholate-3-sulfate	7.60	0.68	0.0010
47	HMDB0031031	2-Dodecylbenzenesulfonic acid	1.51	0.61	0.0008
48	HMDB0004225	2-Oxoarginine	1.58	0.80	<0.0001
49	HMDB0000670	Homo-L-arginine	2.74	0.80	<0.0001
50	HMDB0031513	3-hydroxynonanoic acid	4.61	0.67	0.0027
51	HMDB0002207	3-Hydroxyisoheptanoic acid	1.95	0.72	<0.0001
52	HMDB0028853	Glycyltyrosine	1.73	0.73	<0.0001
53	HMDB0013704	6-Methylnicotinamide	2.01	0.80	<0.0001
54	HMDB0000714	Hippuric acid	2.03	0.72	0.0020
55	HMDB0000070	Pipecolic acid	1.66	0.72	0.0003
56	HMDB0004224	N-(o)-Hydroxyarginine	1.70	0.74	<0.0001
57	HMDB0000951	Taurochenodesoxycholic acid	2.41	0.70	0.0001
58	HMDB0011177	Phenylalanylproline	2.00	0.73	<0.0001
59	HMDB0000594	gamma-Glutamylphenylalanine	3.15	0.71	0.0001
60	HMDB0004185	5 Hydroxyindoleacetylglycine	1.89	0.70	0.0003
61	HMDB0003426	Pantetheine	6.72	0.67	0.0011
62	HMDB0002596	Deoxycholic acid 3-glucuronide	3.38	0.70	0.0002
63	HMDB0002082	Bisnorcholic acid	2.03	0.73	<0.0001
64	HMDB0011166	L-beta-aspartyl-L-leucine	2.93	0.74	<0.0001
65	HMDB0002183	Docosahexaenoic acid	2.31	0.65	0.0009
66	HMDB0003871	13-L-Hydroperoxylinoleic acid	2.69	0.65	0.0005
67	HMDB0003876	15(S)-HETE	3.59	0.60	0.0011
68	HMDB0000648	Galactosylsphingosine	3.56	0.65	0.0022
69	HMDB0000138	Glycocholic acid	2.76	0.80	0.0037
70	HMDB0001220	Prostaglandin E2	4.98	0.67	0.0025
71	HMDB0000782	Octadecanedioic acid	9.57	0.68	0.0014
72	HMDB0010724	3-Oxodecanoic acid	1.59	0.76	<0.0001
73	HMDB0012108	LysoPC(17:0)	2.23	0.68	0.0002
74	HMDB0013250	Myristoylglycine	2.37	0.67	0.0004
75	HMDB0010734	(R)-3-Hydroxy-hexadecanoic acid	5.51	0.66	0.0020
76	HMDB0010382	LysoPC(16:0)	2.21	0.83	<0.0001
77	HMDB0010384	LysoPC(18:0)	1.55	0.67	0.0022
78	HMDB0013122	LysoPC(P-18:0)	2.06	0.72	<0.0001
79	HMDB0061657	3-hydroxypentadecanoic acid	1.65	0.75	<0.0001
80	HMDB0000673	Linoleic acid	2.64	0.75	<0.0001
81	HMDB0003073	Gamma-Linolenic acid	2.40	0.65	0.0025
82	HMDB0004669	9-OxoODE	5.81	0.67	0.0017
83	HMDB0061655	3-hydroxytridecanoic acid	1.60	0.67	0.0024
84	HMDB0004668	13-OxoODE	3.53	0.66	0.0005

85	HMDB0013333	3-Hydroxy-9-hexadecenoylcarnitine	2.65	0.71	0.0002
86	HMDB0000319	18 Hydroxycorticosterone	1.70	0.74	<0.0001
87	HMDB0002925	8,11,14-Eicosatrienoic acid	1.94	0.68	0.0001
88	HMDB0000529	5-Dodecenoic acid	2.20	0.72	<0.0001
89	HMDB0006528	Docosapentaenoic acid	1.56	0.69	0.0002
90	HMDB0002172	N1,N12-Diacetylspermine	2.57	0.76	<0.0001
91	HMDB0010378	5,8,11-Eicosatrienoic acid	8.37	0.77	<0.0001
92	HMDB0060043	13-HDoHE	1.84	0.64	0.0027
93	HMDB0002231	Eicosenoic acid	5.86	0.74	0.0001

Note: RT[a], retention time.

S.Table 12 Candidate biomarkers and panels screened out from this comprehensive analysis for colorectal cancer diagnosis based on metabolomics studies

Datasets	Metabolic Biomarkers			Panels	Metabolic Biomarkers	
**Dataset A**	L-Tryptophan	IDP	LysoPC(P-18:0)	**Panel 1**	L-Tryptophan	Glycochenodeoxycholat
	13-L-Hydroperoxylinoleic acid	Taurochenodesoxycholic acid	LysoPC(18:0)		13-OxoODE	e LysoPC(16:0)
	Gamma-Linolenic acid	Glycochenodeoxycholate	LysoPC(16:0)		IDP	
	Linoleic acid	Glycocholic acid	LysoPC(17:0)			
	13-OxoODE					
**Dataset B**	L-Phenylalanine	Inosine	LysoPC(16:0)	**Panel 2**	L-Phenylalanine	Inosine
	L-Tryptophan	Uric acid	LysoPC(18:2(9Z,12Z))		Linoleic acid	Glycocholic acid
	Linoleic acid	Allantoic acid	LysoPC(P-18:1(9Z))		Citric acid	LysoPC(14: 0)
	13S-hydroxyoctadecadienoic	Glycocholic acid	LysoPC(24:0)			
	acid	Glycochenodeoxycholate	LysoPC(20:3(5Z,8Z,11Z))			
	13-L-Hydroperoxylinoleic acid	Taurocholic acid	LysoPC(20:0)			
	13-OxoODE	Chenodeoxycholic acid	LysoPC(18:3(6Z,9Z,12Z))			
	Citric acid	Taurochenodesoxycholic acid	LysoPC(18:0)			
	Fumaric acid	LysoPC(14:0)	LysoPC(16:1(9Z))			
	Isocitric acid					
**Dataset C**	L-Tryptophan	13-OxoODE	Taurochenodesoxycholic acid	**Panel 3**	L-Tryptophan	Glycocholic acid
	Linoleic acid	Glycocholic acid	LysoPC(16:0)		Linoleic acid	LysoPC(16:0)
	13-L-Hydroperoxylinoleic acid	Glycochenodeoxycholate	LysoPC(18:0)			

S.Table 13. The diagnostic efficacy of different tumor stages of the biomarker panels

TNM	First stage				Second stage			
	AUC（95% CI）	*P*-value	Sensitivity	Specificity	AUC（95% CI）	*P*-value	Sensitivity	Specificity
**Ⅰ/Ⅱ (case/control: 36/68)**					**Ⅰ/Ⅱ (case/control: 54/127)**			
Panel 1	0.989(0.976-1.000)	0.000	0.941	0.972	0.960(0.935-0.985)	0.000	0.858	0.926
Panel 2	0.968(0.939-0.997)	0.000	0.926	0.889	0.945(0.909-0.981)	0.000	0.937	0.870
Panel 3	0.939(0.884-0.994)	0.000	0.926	0.917	0.933(0.894-0.973)	0.000	0.874	0.889
**Ⅲ/Ⅳ (case/control: 28/68)**					**Ⅲ/Ⅳ (case/control: 37/127)**			
Panel 1	0.976(0.943-1.000)	0.000	0.956	0.964	0.964(0.939-0.989)	0.000	0.866	0.973
Panel 2	0.976(0.952-1.000)	0.000	0.897	1.000	0.953(0.920-0.986)	0.000	0.929	0.865
Panel 3	0.940(0.880-1.000)	0.000	0.912	0.929	0.945(0.907-0.983)	0.000	0.945	0.838
**All (case/control: 66/68)**					**All (case/control: 104/127)**			
Panel 1	0.982(0.963-1.000)	0.000	0.956	0.970	0.963(0.943-0.984)	0.000	0.858	0.962
Panel 2	0.971(0.949-0.994)	0.000	0.897	0.939	0.948(0.921-0.976)	0.000	0.945	0.856
Panel 3	0.942(0.901-0.983)	0.000	0.897	0.924	0.937(0.906-0.968)	0.000	0.811	0.933

S.Table 14. Characteristics of metabolomics studies of colorectal cancer included in this systematic review

First Author[ref]year	Study Design	Number of Subjects Subjects Recruitment	Matched (Cases/Controls)	Sample Source	Sample Type	Country (Institution)	Platform	Metabolites of colorectal cancer
Djukovic, D. [1]2018	Case-control study	Serum samples were obtained from patients undergoing either colonoscopy for CRC screening or CRC surgery after overnight fasting and identical bowel preparation prior to their procedure. Samples were collected from CRC patients (n=66), polyp patients (n=76), and healthy controls (n=92).	Age- and gender-matched	Hospital	Serum	United States (Purdue University and Indiana University School of Medicine)	LC-MS	2'-deoxyuridine, Arginine, Dimethylglycine, Glutamic acid, Glutamine, Glyceraldehyde, Glycochenodeoxycholate, Glycocholate, Hippuric acid, Histidine, Hydroxyproline/Aminolevulinate, Linolenic acid, Lysine, Methionine, N-Acetyl-glycine, Uridine
Farshidfar, F. [2]2018	Case-control study *(two-stage case-control design)	Samples were collected from CRC patients (N=62), adenoma (N=31), and controls (N=81). Training set: 32 CRC and 21 disease-free controls. Validation set: 28 CRC and 20 healthy controls. Colorectal cancer patients were diagnosed at the	Age- and gender-matched	Hospital	Serum	Canada (University of Calgary, Foothills Medical Center)	FIA-MS/MS	C10:1, C16, C16:1, C16:1-OH, C18:1, C18:2, C3, C3:1, C3-OH, Gln, lysoPC a C24:0, lysoPC a C26:0, lysoPC a C26:1, lysoPC a C28:0, lysoPC a C6:0, Met, Orn, PC aa C24:0, PC aa C26:0, PC aa C40:1, PC aa C40:2, PC aa C40:3, PC aa C42:2, PC aa C42:4, PC aa C42:5, PC ae C30:1, PC ae C30:2, PC ae C36:1, PC ae C36:5, PC ae C38:1, PC ae C38:2, PC ae C38:3, PC ae C38:6, PC ae C40:1, PC ae C40:2, PC ae C40:3, PC ae C40:4, PC ae C40:5, PC ae C42:1, PC ae C42:2,

		Foothills Medical Center. Disease-free controls consisted of individuals who underwent screening colonoscopy and were found not to have CRC or adenoma.						PC ae C42:3, PC ae C42:4, PC ae C42:5, PC ae C44:3, Ser, SM C22:3, Trp, Val
Shu, X. [3]2018	Nested case -control study	Samples were collected from female 122 pairs (case/control) and male 123 pairs (case/control).	Matched (matched to the case on age (± 2 years), sex, date of sample collection (± 30 days), time of sample collection (morning or afternoon), time interval after last meal (± 2 hours), recent antibiotic use (yes or no), and menopausal status (for women))	Populati on	Plasm a	China (Shanghai Women's Health Study (SWHS) and the Shanghai Men's Health Study (SMHS))	GC-T OFMS UPLC-QTOF/ MS	MG(0:0/18:3/0:0), PE(20:0/18:2), Tetracosanoic acid, PE(22:6/p-18:1), PE(p-16:0/20:4), PC(22:6/18:0), 5,6:8,9-diepoxyergost-22-ene-3,7beta-diol, PE(22:6/16:0), PE(p-18:0/18:2), PS(18:0/18:0), PE(o-18:1/20:4), 2,3-epoxymenaquinone, Butenylcarnitine, PE(18:3/18:0), PC(18:3/16:0), PE(p-16:0/18:1), PC(16:0/16:0), Ethyl 4-(methylthio)butyrate, 13'-carboxy-alpha-tocopherol, N-undecylbenzenesulfonic acid, 2-dodecylbenzenesulfonic acid, 4-hydroxy-5-(dihydroxyphenyl)-valeric acid-o-methyl-o-sulphate, Coumarin, 3-aminobenzoic acid, 2-methyl-4-phenyl-2-butyl 2-methylpropanoate, Benzoic acid, Selenocystine, Isoputreanine, Glutamyl-glutamate, Homocarnosine, 2-keto-glutaramic acid, Picolinic acid,

								4-amino-1-piperidinecarboxylic acid, 2-acetyl-5-methylpyridine, 1-deoxy-d-xylulose 5-phosphate
Venalainen, M.K. [4]2018	Case-control study	Samples were collected from 116 patients including 57 patients with CRC (38 colonic, 19 rectal), 13 with IBD, 12 with adenoma, and 34 healthy controls (19 patients scheduled for hernia repair and 15 for laparoscopic cholecystectomy)	NA.	Hospital	Urine	Singapore (Seinajoki Central Hospital from Kuopio University Hospital)	LC-MS/MS	N1,N12-diacetylspermine
Chen, C. [5]2017	Case-control study	Samples were collected from CRC patients (n=36), polyp patients (n=39), and healthy controls (n=83).<br>All consenting participants undergoing colonoscopy or CRC surgery were evaluated.	NA.	Hospital	Serum	United States (Purdue University and Indiana University School of Medicine)	LC-MS/MS	2′-Deoxyuridine, Alanine, Alpha-ketoglutaric acid, Aminolevulinate, Aspartic acid, Cystathionine, Epinephrine, glucose-1,6-bisphosphate, Glutamine, Glyceraldehyde, Hippuric acid, Histidine, Hydroxyproline, Linoleic acid, Linolenic acid, Lysine, Methionine, Orotate, Phenylalanine, Xanthine, Xanthosine
Guertin, K.A. [6]2017	Nested case -control study	Samples were collected from ATBC Study. All identified colorectal cancer cases (n=644) and an equivalent number of controls.	Age- matched	Population	Serum	Finland (The Alpha Tocopherol, Beta Carotene	LC-MS/MS	choline

		Incident colorectal cancer cases in ATBC were identified by the Finnish Cancer Registry.				Cancer Prevention (ATBC) Study)		
Jing, Y. [7]2017	Case-control study	Samples were collected from 81 polyp patients (47 men and 34 women) and 85 CRC patients (50 men and 35 women).<br>Polyps and CRC were identified by colonoscopy and confirmed by histology.	Non-matched	Hospital	Dried blood spot	China (The First Affiliated Hospital of Jinzhou Medical University.)	AB Sciex 4000 QTrap system	3-hydroxyisovalerylcarnitine, alanine, arginine, asparagine, aspartate, butyrylcarnitine, C18:1, cisovalerylcarnitine, citrulline, glutarylcarnitine, methionine, myristoylcarnitine, octadecanoylcarnitine, octanoylcarnitine, ornithine, palmitoylcarnitine, phenylalanine, propionylcarnitine, tyrosine, valine
Kinross, J. [8]2017	Self-control study	Samples were collected from 18 tumor tissue and at 5 cm and 10 cm away from the tumor.<br>Patients with histologically confirmed invasive malignancy or high-grade dysplasia of the colon, having either open or laparoscopic surgery without the use of bowel preparation.	Paired tissue	Hospital	Tissue	United Kingdom (St Mary's Hospital (London, UK))	NMR	Acetate, Alanine, Creatine, Formate, Glycerophosphorylcholine, Glycine, Iso-butyrate, Lactate, Leucine, Phosphocholine, Scylloinositol, Taurine, Valine
Mika, A. [9]2017	Case-control study	Blood samples were collected from 19 patients with stage I-III CRC and 17	Non-matched for blood;<br>Paired tissue	Hospital	#Serum/Tissue	Poland (Medical University	GC-MS	14:0, 14:1, 16:0, 16:1, 18:0, 18:1, 18:3n-3, 20:0, 20:4n-6, 20:5n-3, 22:0, 22:1, 22:6n-3, 24:0, 24:1, 26:0

		healthy volunteers (10 male/7 female), tissue samples were collected from 19 patients with stage I-III CRC				of Gdansk)		
Ning, W. [10]2017	Self-control study	Fresh CRC tissues and their matched adjacent nontumor tissues (located more than 5cm from the tumor) were collected from 20 CRC patients.	Paired tissue	Hospital	Tissue	China (China-Japan Friendship Hospital)	GC-TOF/MS	Alanine, Aspartic acid, Glucose, Glutamic acid, Glycine, Hypoxanthine, Inositol, Isoleucine, Lactic acid, Leucine, Mannose, Methionine, Norleucine, O-phosphorylethanolamine, Oxo-proline, Palmitic acid, Phenylalanine, Phosphate, Proline, Serine, Threonine, Tyrosine, Uracil, Valine
Nishiumi, S. [11]2017	Case-control study	Samples were collected from 282 colorectal cancer patients and 291 healthy volunteers.<br>Patients who were diagnosed with stage 0, I or II colorectal cancer at the National Cancer Center Hospital; were histologically confirmed to have adenocarcinoma.<br>The control plasma samples were obtained from healthy	Matched (gender, age, and the year of blood collection)	Hospital	Plasma	Japan (National Cancer Center Hospital)	GC/QqQMS	2-aminobutyric acid, 2-ketoglutaric acid, 3-hydroxyisovaleric acid, Arabinose, Arabitol, Cysteine, Elaidic acid, Fructose, Fumaric acid, Glycolic acid, Isocitric acid, Lactic acid, Leucine, Lysine, Malic acid, Maltose, meso-erythritol, Ornithine, Palmitoleic acid, Proline, Pyruvic acid, Sorbose, Sucrose, Threitol, Tryptophan, Uric acid, Valine, Xylitol, Xylose

		individuals who underwent cancer screening at the Research Center for Cancer Prevention and Screening.						
Shen, S. [12]2017	Case-control study	Samples were collected from 25 CRC patients (aged 31–80 years) and 10 healthy controls.<br>The CRC patients were categorized according to histopathological features and confirmed by immunohistochemistry.	NA.	Hospital	plasma	China (Affiliated Hospital of Guangdong Medical University)	LC-QToF/MS	5M(38:8), Cer(36:1), Cer(44:5), DG((40:7), DG(37:1), DG(38:6), FFA(20:1), FFA(20:2), FFA(22:4), FFA(ZZ:2), GalCer(33:2), GalCer(34:1), GalCer(36:4), GalCer(41:2), GalCer(42:2), GalCer(42:3), GluCer(42:3), LacCer(35:1), LacCer(38:1), LacCer(40:1), LacCer(40:2), LacCer(40:4), LacCer(42:4), LPC(14:0), LPC(18:2), LPC(18:3), LPC(20:3), LPS(20:4), PC(32:2), PC(34:3), PC(34:4), PC(40:5), PC(O-34:3), PC(O-35:4), PC(O-40:5), PE(32:1), PE(34:1), PE(36:4), PE(36:5), PE(38:2), PE(38:6), PE(40:6), PE(40:7), PE(42:1), PE(O-34:1), PE(O-34:2), PE(O-34:3), PE(O-36:2), PE(O-36:3), PE(O-36:5), PE(O-38:3), PE(O-38:5), PE(O-38:6), PE(O-40:3), PEIO-36:4), pG(34:0), PS(37:2), SM(32:2), SM(37:1), SM(42:2), SM(44:2), TG(52:1), TG(52:2), TG(56:6)
Uchiyama, K. [13]2017	Case-control study	Samples were collected from CRC patients (n=56), colonic adenoma patients (n=59), and healthy controls (n=60)	Age- and gender-matched	Hospital	Serum	Japan (Hospital of the Kyoto Prefectural	CE-TOFMS	10-Hydroxydecanoic acid, 2-Aminoisobutyric acid, 2-Hydroxybutyric acid, 2-Oxoisovaleric acid, 3-Hydroxybutyric acid, 3-Indoxylsulfuric acid, 3-Methylhistidine, 4-Pyridoxic acid, ADMA,

						University of Medicine)		Ala, Arg, Asn, Benzoic acid, beta-Ala, Betaine, Cholic acid, Choline, cis-Aconitic acid, Citric acid, Citrulline, Cystine, Decanoic acid, Diethanolamine, Dyphylline, Ethanolamine, Ethanolamine phosphate, Gln, Gluconic acid, Glucuronic acid, Glu-Gly/Gly-Glu, Gly, Glycerol 3-phosphate, Gly-Thr/Ser-Ala, Hippuric acid, His, Homovanillic acid, Hydroxyproline, IndolE-3-acetic acid, Isethionic acid, Isobutyric acid, Isocitric acid, Isovaleric acid, Kynurenine, Lauric acid, Lys, Met, Mucic acid, N2-Phenylacetylglutamine, O-Acetylcarnitine, Octanoic acid, Octanoylcarnitine, Ornithine, Pelargonic acid, Perillic acid, Phe, Pipecolic acid, Pro, Sarcosine, S-Methylcysteine, Stachydrine, Succinic acid, Taurine, Thr, Threonic acid, Trimethylamine N-oxide, Trp, Tyr, Uric acid, Uridine, Val
Wang, X. [14]2017	Case-control study	Samples were collected from the 15 patients with colorectal cancer (nine males and six females) and 12 healthy control individuals. All individuals come from the Physical Examination Center at the Department of	NA.	Hospital	Feces	China (Third Hospital Affiliated of Nanchang University)	GC-MS	Aspartic acid, Cholesterol derivatives, Elaidic acid, Glutamic acid, Glycerin, Glycerin, Glycine, Leucine, Linoleic acid, Monoacyl glycerol, Myristic acid, Oleic acid, Pantothenic acid, Phenylacetic acid, Phenylalanine, Proline, Propionic acid, Serine, Ursodesoxycholic acid, Valine

		Gastroenterology.						
Wang, Z. [15]2017	Case-control study	Samples were collected from CRC patients (n=55) and healthy controls (n=40). Patients did not receive any neoadjuvant chemotherapy or radiation therapy prior to sample collection. Healthy controls were age- and gender-matched patients, and had no declared history of cancer or gastrointestinal symptoms.	Age- and gender-matched	Hospital	Urine	China (Second Affiliated Hospital of Shantou University Medical College)	NMR	Acetoacetate, Alanine, Asparagine, Choline, cis-Aconitate, Creatinine, Cysteine, Dimethyl sulfone, Glutamine, Guanidoacetate, Hippurate, Homocysteine, Isocitrate, Methylamine, Phenylalanine, trans-Aconitate
Zhang, L.J. [16]2017	Case-control study	Samples were collected from 25 CRC patients and 10 healthy volunteers.	Non-matched	Hospital	Serum	China (Affiliated Hospital of GuangDong Medical University)	UPLC-MS/MS	12-keto-LTB4, 13-HODE, 13-HpODE, 13-KODE, 19-HETE, 9-HODE, 9-HpODE, 9-KODE
Yan, L. [17] 2016	Case-control study	Samples were collected from 68 cancer patients and 32 healthy controls. The CRC patients were diagnosed by microscopy, biopsy, or surgical resection.	Non-matched	Hospital	Feces	China (Second affiliated hospital of Shantou University Medical	^{1}H NMR	Acetate, Alanine, Butyrate, Dimethylglycine, Glucose, Glutamate, Glutamine, Isoleucine, Lactate, Leucine, proline, Propionate, succinate, Valine

						College)		
Gao, P.[18] 2016	Self-control study	Set 1: 11 cancer and corresponding paracancerous tissues<br>Set 2: 22 cancer samples and 10 advanced adenoma samples	Set 1: paired tissue<br>Set 2: Non-matched	Hospital	Tissue	China (Dalian University Affiliated Xinhua Hospital)	CE–MS	Betaine, Choline, Cysteine, Glutathione, Glycine, Methionine, S-adenosylhomocysteine, S-adenosylmethionine, Sarcosine, Serine, Taurine, Threonine
Yuan, T.[19] 2016	Self-control study	CRC tumors including 16 colon cancer and 34 rectal cancer together with the corresponding adjacent non-involved tissues (ANIT) were collected from 50 CRC patients。	Paired tissue	Hospital	Tissue	China (Zhongnan Hospital of Wuhan University)	HR MAS NMR and GC-FID/MS	Lipid, Lactate, Leucine, Valine, Isoleucine, Alanine, Glutamine, Glutamate, Aspartate, Aspargine, Cysteine, Glycine, Tyrosine, Phenylalanine, Choline, PC/GPC, PE, Scyllo-inositol, GSH, Taurine, Uracil, Cytosine, Isocytosine, Inosine, C20:1n9, C20:2n6, C18:3n3, C18:1n9, C18:2n6, UFA, MUFA, PUFA, n6, n3, ToFA
Crotti, S. [20] 2016	Case-control study *(two-stage case-control design)	For the untargeted lipidomic approach, samples were collected from 15 CRC patients and 15 healthy subjects who underwent colonoscopy and for whom a negative result was attained. For the targeted analysis, plasma samples from 48 CRC patients at surgery. Control plasma samples	Non-matched	Hospital	Plasma	Italy (First Surgical Clinic of Padua Hospital)	GC-MS	Decanoic acid, Dodecanoic acid, Hexanoic acid , Octanoic acid

		were obtained from 20 healthy subjects who underwent colonoscopy but for whom no pathological affliction was found。						
Farshidfar, F. [21] 2016	Nested case-control study	Samples were collected from 320 cancer patients, 31 adenomas, and 254 healthy controls.<br>Disease-free controls consisted of healthy individuals who had a normal screening colonoscopy.	Age- and gender-matched	Population	Serum	Canada (Forzani and MacPhail Colon Cancer Screening Centre at the University of Calgary)	GC-MS	2-Monostearoylglycerol, Alanine, Arabinose, Butanoic acid, 2-hydroxy-, Butanoic acid, 3-hydroxy-, Citric acid, Cystine, D-Ribose 5-phosphate, Ethylene glycol, di-TMS, Fructose, Glyceric acid, Glycerol, Glycerol-3-phosphate, Glyceryl palmitate, Glycine, Hexadecanoic acid, Inositol, myo-, Isoleucine, Lactic acid, Linoleic acid, Lysine, Malonic acid, Bis TMS, Nonane, Palmitic acid, Phenylalanine, Stearic acid, trimethylsilyl ester, Threonic acid, Threonine, allo-, Tyrosine
Sinha, R. [22]2016	Case-control study	Samples were collected from 69 cancer patients, 114 controls. Newly diagnosed cases with adenocarcinoma of the colon or rectum were recruited prior to surgery and treatment. Controls were the patients awaiting elective surgery for non-oncologic, nongastrointestinal	Gender- and BMI- matched	Hospital	Feces	United States (National Cancer Institute)	HPLC-GC/MS-MS	Palmitoyl_Sphingomyelin, p_Hydroxybenzaldhyde, Conjugated linoleic acid, p_Aminobenzoate, Alpha_Tocopherol

		conditions at these hospitals during the same period.						
Zhang, H. [23]2016	Self-control study	Samples were collected from 125 CRC patients and a total of 166 surgical specimens. Among them, 41 normal control tissues were extracted at least 5-10 cm away from the edge of a tumor from the same sample, thus 82 cases included matched tumor and normal control tissues.<br>The pathological diagnosis of CRC was confirmed using routine histopathological H&E stained specimens.	Paired tissue	Hospital	Tissue	China (West China Hospital of Sichuan University)	NMR	2-Hydroxyisovaleric acid, Acetate, Acetic acid, Acetoacetate, Adenine, Alanine, α-ketoglutarate, Arginine, β-hydroxybutyrate, Creatine, Creatinine, Dimethylamine, DMG, Formate, Glucose, Glutamine, Glutathione, Glycolate, GPC, Isoleucine, Lactate, Leucine, Lipid -CH2-C=O, Lipid -CH2-CH=CH, Lysine, Methylamine, myo-inositol, O-acetyl glycoprotein, PC, Phosphocreatine, Sarcosine, Serine, Succinate, Taurine, Threonine, Thymine, TMA, Trimethylamine-N-oxide, Tryptophan, Uracil, Valine, VLDL: -CH2-CH2-CH2O
Amal, H. [24] 2016	Case-control study	Samples were collected from 65 cancer patients, 22 adenomas and 122 controls. Control group was defined as patients without adenocarcinoma or adenomatous polyps.	Non-matched	Hospital	Breath	Latvia (Riga East University hospital or Digestive Diseases Centre GASTRO (Riga,	GC-MS	4- methyl octane, Acetone, Ethanol, Ethyl acetate

						Latvia))		
Williams, M.D. [25] 2015	Self-control study	Samples included 9 tumor and 9 adjacent non-malignant tissue	Paired tissue	Hospital	Tissue	United States (Providence Regional Cancer Center, Sacred Heart Hospital, Spokane)	UPLC-TWIM MS	1,3-Bisphosphogylcerate, 12-Ketodeoxycholic acid, 2,3-Diphosphoglyceric acid, 3′ AMP, 5-Aminoimidazole ribonucleotide, 5-Methylcytidine, 7-Ketodeoxycholic acid, AMP, Arginine, Ascorbic acid, Asparaginyl-Glutamine, Carnitine, Citicoline, CL(18:1/18:1/20:4/18:2), CMP, CoA-[4'-phosphopantetheinel], CPA(18:0/0:0), Cysteineglutathione disulfide, Cytidine, Galactinol, Ganglioside GM1(d18:0/12:0), Glanglioside GM2(d18:1/18:1), Glutathione, Glutathione[M+K], Glycerylphosphorylethanolamine, Hypoxanthine, Inosine, LPA(0:0/18:0), L-Thyronine, LysoPC(14:0), LysoPC(16:0)[M+K], LysoPC(18:1)[M+H], LysoPC(18:1)[M+K], LysoPC(18:1)[M+Na], LysoPE(0:0/20:4), Malic acid, Me-FapyGua, N5-formyl-THF, N-acetylneuraminate, N-Succinyl-L,L-2,6-diaminopimelate, Palmitic amide, PC(14:1/22:1), PC(18:1/16:0), p-Cresol sulfate, PGP(16:1/22:6), PIP(16:0/18:0), PS(14:1/14:0), Ribonic acid, Serinyl-Methionine, Succinyladenosine, Taurine, Threoninyl-Aspartate, Threoninyl-Serine,

								UDP-D-Xylose, UMP, Uridine diphosphate glucose, Uridine diphosphate-N-acetylglucosamine, Xanthine
Zamani, Z. [26] 2014	Case-control study	Samples were collected from 33 CRC patients and 33 individuals without colon cancer.<br>Patients were diagnosed with cancer by colonoscopy and biopsy.	NA	Hospital	Serum	Iran (Amir Alam Hospital, Tehran)	NMR	3-Hydroxybutyric acid, 3-Hydroxyisovaleric acid, 5-Methylcytidine, 7-Ketocholesterol, Beta-leucine, Cholesterol, Glycine, Glycocholic acid, L-fucose, L-palmitoylcarnitine, Orotidine, Pyridoxamine, Pyridoxine, S-adenosylhomocysteine, Taurocholic acid
Cross, A.J.[27] 2014	Nested case-control study	Samples were collected from 254 first primary incident colorectal cancers (International Classification of Diseases for Oncology10, ICD-0-3 codes: C180-189, C199, C209, C260)<br>Controls (254) were free from any cancer at the time the matched case was diagnosed.	Age-, gender, and smoking status matched	Population	Serum	United States (The Prostate, Lung, Colorectal, and Ovarian cancer screening (PLCO) trial, which is a large randomized controlled trial to test the efficacy	UPLC-MS/MS<br>GC-MS	Leucyl-leucine, Fumarate, 10-undecenoate (11:1n1), Xanthine, Stearate, Glycochenodeoxycholate, Andro steroid monosulfate 2, Alpha-tocopherol, 17-methylstearate, Palmitate, Eicosenoate (20:1n9 or 11), Margarate, Dihomo-linoleate (20:2n6), Dihomo-linolenate, Cyclo(phe-phe), Octadecanedioate, Docosapentaenoate, N-acetylserine, 13-methylmyristic acid, Leucylalanine, Methyl palmitate (15 or 2), 2-hydroxystearate, Scyllo-inositol, Pentadecanoate, 2-hydroxypalmitate, Stearidonate (18:4n3), Glutaroyl carnitine, Glycerate, N-acetylneuraminate, Phenol sulfate, Myristate, Pantothenate, Taurochenodeoxycholate, Pyridoxate,

						of screening methods for each of these 4 cancers)		Glycocholate, Taurolithocholate-3-sulfate, Taurodeoxycholate, Glycylvaline, Glycoursodeoxycholate, Pyroglutamine, Glycolate-hydroxyacetate, Pyroglutamylglycine, Glycolithocholate sulfate, N-acetylalanine, 1-palmitoyl glycerophosphoethanolamine, Succinate, 2-hydroxybutyrate (AHB)
Goedert, J.J. [28] 2014	Case-control study	Samples were collected from 48 cancer patients and 102 controls (contemporaneous patients awaiting elective surgery for non-oncologic, non-gastrointestinal conditions).<br>Only newly diagnosed, histologically confirmed cases of adenocarcinoma of the colon or rectum were retained.	Gender- and BMI matched	Hospital	Feces	United States (Three Washington DC area hospitals)	HPLC-GC/MS-MS	2-Hydroxyacetaminophen sulfate, 3-Cystein-S-YL-acetaminophen, 3-Dehydrocarnitine, 4-Acetamidophenol, Alanyl-histidine, Alanyl-leucine, Alanyl-tryptophan, α-Glutamyl-valine, α-Tocopherol, Cis-Urocanate, Conjugated linoleate-18-2N7, γ-Tocopherol, Heme, Histidine, Histidyl-alanine, Histidyl-glycine, Histidyl-phenylalanine, Leucyl-glutamate, Leucyl-serine, Leucyl-tryptophan, Mandelate, N-2-Furoyl-glycine, PABA, p-Acetamidophenylglucuronide, Palmitoyl-sphingomyelin, p-Hydroxybenzaldehyde, Prolyl-alanine, Pterin, Pyro-glutamyl-glycine, Sitostanol, Tryptophyl-glycine, Tyrosyl-glutamine, Valyl-aspartate, Valyl-histidine
Bae, S.[29] 2014	Nested case-	Participants were selected from the WHI-OS cohort. A	Matched (matched on	Population	Plasma	United States	LC-MS/MS	Betaine, Choline, TMAO

	control study	total of 835 colorectal cancer patients and 835 controls were finally included.	age (±3 years), race/ethnicity, timing of baseline blood draw (±6 months), enrollment date (±1 year), and baseline hysterectomy status.)			(WHI-OS is a prospective cohort study designed to investigate the predictors and causes of morbidity and mortality in postmenopausal women)		
Wang, C.[30] 2014	Case-control study	Samples were collected from 20 cancer patients and 20 healthy individuals	Non-matched	Hospital	Breath gas	China (First Affiliated Hospital of Harbin Medical University)	GC/MS	2,21-Dimethyldecane, 21-Methylpropanoate, 41-Ethyl-111-octyn1-31-ol, 61-t1-Butyl-12,2,9,91-tetramethyl-13,51-decadien-171-yne, Cyclohexanone, Cyclooctylmethanol, Dodecane 31-hydroxy-12,4,41-trimethylpentyl, Ethylaniline, Trans-121-Dodecen-111-ol
Wang, C. [31] 2014	Case-control study	Samples were collected from 16 CRC patients and 20 healthy volunteers	Non-matched	Hospital	Blood volatile compo	China (First Affiliated	GC/MS	1,1,4,4-Tetramethyl-2,5-dimethylene-cyclohexane, 6-t-Butyl-2,2,9,9-tetramethyl-3,5-decadien-7-yne,

					unds	Hospital of Harbin Medical University)		ethylhexanol, Phenyl methylcarbamate
Mirnezami, R.[32] 2014	Self-control study	Samples were retrieved from the center of tumor (n=44) and from healthy mucosa 5cm from the tumor margin (n=44)	Paired tissue	Hospital	Tissue	United Kingdom (St Mary's Hospital, London)	HR-MAS NMR	Acetate, Alanine, alpha-glucose, beta-glucose, Creatine -CH2-, Creatine -CH3, Glycerophosphorylcholine(GPC), Glycine, Isoglutamine, Lactate -CH-, Lactate -CH3, Leucine, Lipids/triglycerides, Scyllo-inositol, Taurine -CH2-NH, Taurine CH2SO3, Valine
Qiu, Y. [33] 2014	Self-control study	A total of 55 patients contributed paired samples and the other 30 patients contributed colorectal cancer tissue only.<br>In addition, three validation batches of samples were collected from Cancer Hospital affiliated with the Chinese Academy of Medical Sciences (Beijing, China; n=23, paired tissue samples with two batches of nontumor tissues located 5 and 2–5 cm from the edge of the tumor, respectively), the Second	Paired tissue	Hospital	Tissue	China and the United States (Fudan University Shanghai Cancer Center)	GC–TOFMS	2-Aminoadipate, 2-Aminobutyrate, 3-Methy-3-hydrpoxybutyrate, 4-Aminobutyrate, 5-Oxoproline, AMP, Ascorbate, Asparagine, Aspartate, b-Alanine, Cystein, Galactose, Glucose, Glutamate, Glyceraldehyde, Glycerate, Glycerol, Glycine, Homocysteine, Hypotaurine, Hypoxanthine, Kynurenine, Lactate, Laurate, Methioninamide, Myo-inositol, Myristate, Nicotinamide, Ornithine, Palmitoleate, Phosphate, Putrescine, Spermidine, Uracil, Xylose

		Affiliated Hospital, Zhejiang University School of Medicine (Hangzhou, China; n= 65, paired tissue samples), and the City of Hope Comprehensive Cancer Center (Duarte,CA; n=20,pairedtissuesamples).						
Phua, L.C. [34] 2014	Case-control study Self-control study	Fecal samples were collected from 11 cancer patients and 10 healthy subjects. Matched tumor and normal mucosa were collected from 8 out of the 11 CRC patients. Matched normal mucosa samples were obtained from excised tissue at least 5 cm away from the edges of the tumor.	Non-matched for fecal. Paired tissue	Hospital	# Feces /Tissue	Singapore (Singapore General Hospital)	GC/TOFMS	3-phosphoglycerate, Citric acid, Creatinine, Fructose , Galactose, Glucose, Inosine, Linoleic acid, Nicotinic acid, Proline, Uracil, Uridine
Jiménez, B. [35] 2013	Self-control study	Samples were retrieved from tumor (n=22) and adjacent normal colorectal mucosa 5 to 10 cm from the tumor margin (n=23). There were 19 matched	Paired tissue	Hospital	Tissue	United Kingdom (St Mary's Hospital (London, UK))	HR-MAS NMR	Acetate, Alanine, β-Glucose, Choline, Creatine, Formate, Iso-butyrate, Iso-glutamine, Lactate, Leucine, Lipids/Triglycerides, Phenylalanine, Phosphocholine, Scyllo-inositol, Taurine, Tyrosine, Valine

		paired samples, 3 additional tumor samples and 4 additional off-tumor samples.						
Kim, S. [36] 2013	Self-control study	Samples were collected from 9 cancer patients. Tissue was divided into normal and cancer samples according to biopsy.	Paired tissue	Hospital	Tissue	Korea (Jeju National University Hospital)	HR-MAS NMR	2-Hydroxybutyrate, Acetoacetate, Arginine, Aspartate, Carnitine, Ethylmalonate, Glutamate, Glutathione, Glycine, Isoleucine, Leucine, Malonate, Myo-inositol, N-Acetylglutamine, O-Phosphocholine, Proline, Taurine, Uracil
Li, F. [37]2013	Case -control study	Samples were collected from 52 CRC patients and 52 healthy controls.<br>The healthy controls were selected by routine clinical examination and the subjects with any clinically diagnosed acute or chronic disease were excluded.	Matched	Hospital	Serum	China (Peking Union Medical Hospital, Heze Municipal Hospital)	DI-ESI (±)-FTICR MS	eicosatrienoic acid, hexadecanedioic acid, LPA(16:0), LPA(18:0), LPC(16:0), myristic acid, octadecanoic acid, oleamide, palmitic amide
Weir, T.L. [38] 2013	Case-control study	Stool samples were collected from healthy individuals (n=11) and recently diagnosed colon cancer patients (n=10) prior to surgery for colonic resection. Stool samples were provided for analyses prior to administration of any	Non-matched	Hospital	Feces	United States (University of Colorado Health-Poudre Valley Hospital in Fort Collins, CO.)	GC-MS	Alanine, Aspartic acid, Benzeneacetic Acid, Cholesterol derivative, Elaidic acid, Glutamate, Glycerol, Glycine, Leucine, Linoleic acid, Lysine, Monooleoylglycerol, Myristic Acid, Oleic acid, Pantothenic acid, Phenylalanine, Proline, Propionic acid, Serine, Threonine, Ursodeoxycholic acid, Valine

		preoperative antibiotics or bowel preparation.						
Tan, B.[39] 2013	Case-control study *(two-stage case-control design)	Learning group: 62 samples from cancer patients and 62 from healthy subjects. Validation group: 39 samples from cancer patients and 40 from healthy subjects (Healthy controls with inflammatory conditions or gastrointestinal tract disorders were excluded).	Age- and gender-matched	Hospital	Serum	China (Ruijin Hospital affiliated with Shanghai Jiao Tong University School of Medicine)	GC−TOFMS and UPLC−QTOFMS	12a-hydroxy-3-oxocholadienic acid, 2-aminobutanoic acid, 2-hydroxy-3-methylpentanoic acid, 2-hydroxybutyric acid, 2-oxobutanoic acid, 3,4,5-trimethoxycinnamic acid, 3-oxodecanoic acid, 4-hydroxyproline, 4-hydroxystyrene, 5-hydroxytryptamine, 6-phosphogluconic acid, acetyl carnitine, adenine, alanine, allantoic acid, allisoleucine, allyl isothiocyanate, alpha-aminoadipic acid, asparagine, aspartate, benzaldehyde, beta-aspartylserine, beta-hydroxybutyrate, carnitine (18:1), chenodeoxycholic acid, cholic acid, cis-aconitate, CPA(18:0/0:0), creatinine, cystine, decanoyl carnitine, elaidic acid, erythrotetrofuranose, fumaratea, glutamate, glyceric acid, glycerol, glycolaldehyde, histidine, hydroquinone, indoleacrylic acid, indoxyl, indoxyl sulfate, inositol, linolic acid, LPA(18:0/0:0), LysoPC(14:0), LysoPC(16:1(9Z)), LysoPC(20:0), LysoPC(P-18:1(9Z)), methionine, N-acetyl-5-hydroxytryptamine, octenedioate, oleamide, oleic acid, ornithine, palmitic acida, phenol, phenylalanine, proline betaine, pyruvate,

								ribitol, serine, sphinganine, tetrahydrogestrinone, threitol, trihydroxycoprostanoic acid, trimethylamine N-oxide, tryptophan, ubiquinone, urea, xanthosine
Nishiumi, S. [40] 2012	Case-control study *(two-stage case-control design)	Training set: samples were collected from 60 CRC patients and 60 healthy volunteers.<br>Validation set: samples were collected from 59 CRC patients and 63 healthy volunteers.<br>The patients were diagnosed by microscopy, biopsy, or surgical resection and classified using the sixth edition of the International Union Against Cancer classification (UICC).<br>The serum samples from the healthy volunteers were obtained from Kobe University Hospital and two other facilities. In Kobe University Hospital, it was confirmed that there is no	Age- and gender-matched	Hospital	Serum	Japan (Kobe University Graduate School of Medicine)	GC/MS	2-hydroxy-butyrate, Arabinose, Asparagine, Aspartic acid, b-alanine, Citrulline, Creatinine, Cystamine, Cystine, Glucosamine_2, Glucuronate_1, Glutamic acid, Inositol, Isoleucine, Kynurenine, Lactitol, meso-erythritol, Nonanoic acid(C9), O-phosphoethanolamine, Ornithine, Palmitoleate, Phosphate, p-hydroxybenzoic acid, Pyroglutamic acid, Pyruvate, Ribulose, Xylitol

		abnormality of blood tests, endoscopic examinations, diagnostic imaging, and/or medical interview. At two other facilities, healthy volunteers were selected via health checks including blood tests, endoscopic examinations, diagnostic imaging, and/or medical interviews.						
Chen, J.L.[41] 2012	Case-control study	Urine samples were collected from 20 CRC patients (aged 37 to 87 years old) and 14 healthy volunteers (female/male, 6/8, aged 50 to 86 years old, and the median age was 68 years old) from Shanghai Sixth People's Hospital.	Non-matched	Hospital	Urine	China (Shanghai Sixth People's Hospital, Shanghai Jiaotong University)	CE-ESI-MS	Arginine, Aspartate, Citric acid, Histidine, Isoleucine, Lactic acid, Leucine, Malic acid, Methionine, Serine, Succinate, Valine
Cheng, Y.[42] 2012	Case-control study *(two-stage case-	Training set 1: samples were collected from 61 cancer patients and 62 healthy controls<br>Testing set 2: samples were collected from 40 cancer	Age- and gender-atched	Hospital	Urine	China (Ruijin Hospital (Shanghai, China).)	GC-TOFMS And UPLC-QTOFMS	2-Aminobutyrate, 2-Hydroxyestradiol, 4-Aminohippurate, 4-Hydroxybutyrate, 5-Hydroxy-tryptophan, Acetyl-carnitine, Alanine, Arabitol, Citrate, Creatinine, Fumarate, Glucuronate, Hippurate, Histidinol, Homovanillate, Hydroxyacetate, Indole,

	control design)	patients and 41 healthy controls<br>Patients were categorized according to histopathological features and stages according to TNM classification of malignant tumors.						Indoleacetate, Kynurenate, Myristate, N-Acetyl-L-lysine, p-Cresol, Phenol, Putrescine, Pyridoxal (Vitamin B6), Pyruvate, Sorbose, Threonate, Trimethylamine N-oxide, Tryptophan, Tyrosine, Uracil, Urea, Uridine, Xylose
Mal, M.[43] 2012	Self-control study	Matched tumor and normal tissues (n=63) obtained from 31 patients	Paired tissue	Hospital	Tissue	United States (Singapore General Hospital)	GC×GC/TOFMS	11-eicosenoic acid, 1-methyl-hydantoin, 1-monooleoylglycerol, Aminomalonic acid, Arachidonic acid, β-alanine, Cholesterol, D-galactose, D-glucose, D-mannose, Fumarate, Glycerol, Glycine, Lactateb, L-alanine, L-arabinose, L-aspartic acid, Lignoceric acid, Linolenic acid, L-isoleucine, L-leucine, L-methionine, L-phenylalanine, L-proline, L-serine, L-threonine, L-valine, Malate, Maleic acid, Margaric acid, Myristic acid, Oleic acid, Oxalate, Palmitic acid, Pantothenic acid, Pentadecanoic acid, Phosphate, Picolinic acid, Ribitol, Squalene, Stearic acid, Succinate, Uracil, Uridine
Ma, Y.[44] 2012	Case-control study	Samples were collected from 30 CRC patients and 30 healthy blood donors and 20 fetuses.	Age- matched	Population	Serum	China (Sixth People’s Hospital	GC/MS	1-Deoxyglucose, 3-Hydroxybutyric, Glycine, L-Threonine, L-Valine, Ribitol

		Control blood samples were obtained from healthy blood donors in Shanghai. The age range of the donors matched with those of the patients and the donors participated in strict screening and a follow-up visit. Fetal blood samples were obtained from the Department of gynecology and obstetrics.				Affiliated to Shanghai JiaoTong University)		
Bertini, I. [45] 2012	Case-control study *(two-stage case-control design)	Training set: samples were collected from 45 CRC patients and 96 healthy individuals.<br>Validation set: samples were collected from 108 CRC patients and 43 healthy individuals.<br>Serum samples were collected from a cohort of 181 Danish patients with mCRC resistant to 5-FU, oxaliplatin, and irinotecan.<br>Serum samples of controls were collected from Danish	Non-matched	Population	Serum	Denmark (Three hospitals in Denmark)	^{1}H NMR	3-hydroxybutyrate, Acetate, Alanine, Citrate, Creatine, Formate, Glutamine, Glycerol, Lactate, Leucine, Lipid (-CH2-OCOR), N-acetyl signal of glycoproteins, Peptide NHs, Phelylalanine, Proline, Pyruvate, Tyrosine, Valine

		healthy blood donors and 43 healthy subjects.						
Ong, E.S.[46] 2010	Self-control study	Colonic tissues including tumor, polyps and adjacent mucosa were obtained from 26 CRC patients. Tumor specimens, polyps and adjacent normal-appearing tissues at least 8 cm away were collected from the same patients undergoing colorectal resection according to procedure reported earlier.	Paired tissue	Hospital	Tissue	Singapore (Singapore General Hospital)	GC/MS LC/MS/MS	Acetylcarnitine, Adenosine, Adenosine monophosphate, Arachidicacid(C20:0), Arachidonicacid(C20:4), Behenicacid(C22:0), Betaine, beta-Sitosterol, Carnitine, Cholesterol, Choline, Citramalicacid, Deoxycholicaicd, Elaidiccarnitine, Glucose, Glutamate, Glycerol 1-(9-octadecenoate), Glycerol 1-palmitate, Glycerol 1-stearate, Glycerol 2-palmitate, Glycerophosphocholine, Hypoxanthine, Inosine, Inositolmonophosphate, Lauricacid(C12:0), Linoleicacid(C18:2), LPC C16:0, LPC C18:0, LPC C18:1, LPC C18:2, LPC C20:4, Lysine, Margaricacid(C17:0), Methionine, myo-Inositol, Myristamide, Myristicacid(C14:0), Oleamide, Oleamide, Oleic acid(C18:1), Palmitamide, Palmiticacid(C16:0), Palmitoylcarnitine, Phenylalanine, Phosphoric acid, Proline, Stearamide, Stearicacid(C18:0), Tyrosine, Uric acid, Uridine, Vitamin C, Xanthine
Ma, Y.[47] 2010	Self-control study	Samples were collected from 30 colorectal cancer patients pre-operatively and post-operatively. The preoperative blood	Matched	Hospital	Serum	China (The Sixth People's Hospital Affiliated to	GC/MS	11-cis-octadecenoic acid, 1-Deoxyglucose, 2-hydroxy-1,2,3-propanetricarboxylic acid, 3-Hydroxybutyric acid, 3-Hydroxy-proline, 5-oxo-L-proline, 9,12-octadecadienoic acid, Aminomalonic acid, Arachidonic acid,

		samples were drawn at 7 O'clock on the second day before hospitalization, and the postoperative blood samples were drawn in 2 h after surgery. During or within 2 h after surgery, the patients were not given any blood transfusion or other transfusion.<br>The diagnosis of CRC was confirmed in all cases by histopathologic examination and immunohistochemistry.				Shanghai Jiao Tong University)		Butanedioic acid, Cholesterol, D-maltose, D-turanose, Glycerol, Glycine, Hexadecanoic acid, L-alanine, L-isoleucine, L-proline, L-threonine, L-tyrosine, L-valine, Malic acid, Monostearin, Myo-inositol, Octadecanoic acid, Palmitelaidic acid, Phenylalanine, Phosphoric acid, Ribitol, Serine, Threonine, Trans-9-octadecenoic acid, Tryptophan
Tessem, M.B.[48] 2010	Self-control study	Fresh frozen colon tissue samples (n=63) from two hospitals in Norway were obtained from 31 newly diagnosed cancer patients (mean age: 73 years (range: 48-93), 16 women and 15 men). Two samples were collected from each patient; one from the tumor area and the other from normal mucosa.	Paired tissue	Hospital	Tissue	Norway	HR-MAS NMR	beta-glucose, choline, creatine, glycerophosphorylcholine(GPC), glycine, lactate, myo-inositol, PC , scyllo-inositol, taurine

Wang, W.[49] 2010	Case-control study	Samples were collected from 50 cancer, 34 benign colorectal tumor (23 of ademona and 11 of other diseases) and 34 healthy volunteers.	Matched	Hospital	Urine	China (Ruijin Hospital, Shanghai Jiaotong University School of Medicine)	UPLC Q-TOFMS SPE-HPLC	1-methyladenosine, 2,2-dimethylguanosine, Acetylcarnitine, Acylcarnitine (C13:0), Acylcarnitine (C9:0), Acylcarnitine (C9-OH), Aspartylysine, C11H20O4N2, Cytidine, Leucylproline, N6-methyladenosine, Phenylacetylglutamine, Prolylleucine, Pseudouridine, Uridine
Shureiqi, I. [50]2010	Case-control study	A total of 125 patients divided into three groups: 49 subjects with normal colon, 36 with colonic polyps, and 40 with colorectal cancer. The colorectal cancer group patients' biopsies were obtained from the colorectal cancers and from normal-appearing mucosa at least 10 cm from the cancer.	NA.	Hospital	Tissue	United States (Gastrointestinal clinics at The university of Texas M. D. Anderson Cancer Center and other hospitals within the Texas Medical Center)	LC-MS/MS	13S-hydroxyoctadecadienoic acid
Qiu, Y. [51] 2010	Case-control	Samples were collected from 60 CRC patients and 63	Age-matched	Hospital	Urine	China (Cancer	GC-MS	2-hydroxyhippurate, 3-methyl-histidine, 5-hydroxyindoleacetate, 5-hydroxytryptophan,

	study	healthy volunteers. All the CRC patients were diagnosed with different histopathological features and stages according to recent TNM classification.				Hospital, Shanghai Medical College, Fudan University)		5-oxoproline, Citrate, Glutamate, Histidine, Isocitrate, N-acetyl-aspartate, p-cresol, Phenylacetate, Phenylacetylglutamine, p-hydroxyphenylacetate, Succinate, Tryptophan
Chae, Y.K. [52] 2010	Self-control study	Cancerous and normal tissues were obtained from 12 patients (4 men, 8 women, mean age: 67.75 years, age range: 41 - 85 years) with histologically proven colorectal cancers.	Paired tissue	Hospital	Tissue	Korea	NMR	choline, glucose, glutamate, glycerol, malate, taurine
Chan, E.C.Y.[53] 2009	Self-control study	Matched CRC and normal mucosae (n=63) were obtained from the 31 CRC patients during surgery. Among these subjects, one patient provided two matched pairs of tissues. None of the patients received neoadjuvant chemotherapy or radiotherapy prior to surgical excision.	Paired tissue	Hospital	Tissue	Singapore (Singapore General Hospital)	HR-MAS NMR and GC/MS	11,14-Eicosadienoic acid, 11-Eicosenoic acid, 1-Hexadecanol, 1-Monooleoylglycerol, 1-O-Heptadecylglycerol, 2-Hydroxy-3-methylvalerate, Arachidonic acid, ChoCC, Cholesterol, D-Galactosed, D-Glucosed, D-Mannosed, Fumarate, Glucose, Glycine, Lactate, Lactate, L-Glycine, Lipids, L-Phenylalanine, L-Proline, Malate, Marganic acid, Oleic acid, Palmitic acid, PC, PE, PEG, Phosphate, Propyl octadecanoate, Scyllo-inositol, Stearic acid, Taurine, Uridine
Qiu, Y. [54] 2009	Case-control	Samples were collected from 64 cancer patients and 65	Non-matched	Hospital	Serum	China (Cancer	GC-TOFMS	2-hydroxybutanoic acid, 2-Piperidinecarboxylic acid, 3-hydroxybutanoic acid, 4-hydroxyproline,

	study	healthy individuals.<br>The clinical diagnosis and pathological reports of all the patients were obtained from the hospital. The healthy volunteers, ages 42-69 years, were selected by a routine physical examination and any subjects with inflammatory conditions or gastrointestinal tract disorders were excluded.				Hospital, Shanghai Medical College, Fudan University)	/UPLC −QTOFMS	Arginine, Carnitine, Citrulline, Dopamine, Glutamic acid, Glycerol phosphate, Hippurate, Lactate, Leucine, Lysine, Malic acid, Myristic acid, Nervonic acid, Nicotinamide, Oleamide, Oleic acid, Ornithine, Palmitic acid, Phenylalanine, Proline, Pyruvate, Pyruvic acid, Threonic acid, Threonine, Tryptophan, Tyrosine, Urea, Uridine, Valine
Righi, V. [55] 2009	Self-control study	Samples of 23 subjects, including14 tumor and 14 normal tissues were obtained from 14 colorectal cancer patients; 9 normal tissues were obtained from 9 healthy individuals.	Paired tissue	Hospital	Tissue	Italy	HR-MAS NMR	Ac, choline-containing compounds, creatine, Glu plus Gln, glucose, Lac, lipids, polyols (Myo and Scy) (scyllo-inositol, myo-inositol), Tau.
Hirayama, A.[56] 2009	Self-control study	Tumor and surrounding grossly normal-appearing tissues were obtained from 16 colon cancer patients.	Paired tissue	Hospital	Tissue	Japan	CE-TOFMS	2-aminobutyrate, 4-aminobutyrate, adenine, Alanine, arginine, asparagine, aspartate, beta-alanine, beta-Glu-cys, citrate, Citrulline, cystathionine, cysteine, cystine, F1,6DP, F6P, fumarate, G1P, gluconate, Glutamate, Glycerol-3P, Glycine, GMP, GSH, histidine, Hydroxyproline, hypotaurine, hypoxanthine,

								Isoleucine, lactate, Leucine, lysine, malate, methionine, NADPH, ophthalmate, phenylalanine, proline, putrescine, pyruvate, S7P, SAH, SAM, serine, spermidine, spermine, succinate, taurine, threonine, tryptophan, tyrosine, valine
Hsu, W.Y.[57] 2009	Case-control study	Patients with colorectal cancer (n=26) were collected at the CMUH. All control subjects (n=18) were healthy people who had undergone a routine annual health examination.	Non-matched	Population	Urine	China (China Medical University Hospital)	LC-MS/MS	8-hydroxy-2′-deoxyguanosine, adenosine, cytidine, N2,N2-dimethylguanine, uridine
Mal, M.[58] 2009	Case-control study	Tumor and normal tissue (n=12) were obtained from 6 patients.<br>Another 6 pairs of tumor and normal tissue were obtained from method validation.	Paired tissue	Hospital	Tissue	Singapore (Singapore General Hospital)	GC/MS	1-O-Heptadecylglycerol, Creatinine enol (enolic form), D-Galactose, D-Glucose, D-Mannose, Lactate, L-Glycine, Malate, Marganic acid, Palmitic acid, Propyl octadecanoate, Stearic acid
Monleón, D. [59] 2009	Case-control study	Samples were collected from 21 CRC patients and 11 healthy individuals.<br>A full-length colonoscopy established the diagnosis in every patient and healthy control. All stool samples	Non-matched	Hospital	Feces	Spain (The Instituto Valenciano de Oncologia)	NMR	Acetate , Butyrate, Cysteine, Leucine, Proline

		were collected before surgery or endoscopic examination, with no bowel preparation.						
Piotto, M.[60] 2008	Self-control study	Cancerous and normal tissues were obtained from 44 patients. For 4 patients, only the healthy or the tumoral biopsy was available.<br>All patients underwent a surgical resection of the primary lesion and a radical lymphadenectomy. Neither neo-adjuvant chemotherapy nor radiotherapy was performed prior to the operation.	Paired tissue	Hospital	Tissue	France (Tumor bank of the University Hospitals of Strasbourg, France.)	NMR	Acetate, Alanine, alpha-Glucose, Arginine, Ascorbic acid, Asparagine, Aspartic acid, beta-Glucose, Choline, Creatine, Ethanol, Fatty acids , Glutamate, Glutamine, Glycerol, Glycerophosphocholine, Glycine, Isoleucine, Lactate, Leucine, Lysine, Methionine, Myo-Inositol, Phenylalanine, Phosphoethanolamine, Phosphorylcholine, Proline, Scyllo-Inositol, Serine, Succinic acid, Taurine, Threonine, Tyrosine, Valine
Denkert, C. [61] 2008	Self-control study	In total, 27 primary colon carcinomas and 18 normal mucosa samples. For 15 cases paired samples of caner tissue and normal tissue were available.	Paired tissue	Hospital	Tissue	Germany	GC-TOF	1,4-benzenedi carboxylic acid, 2-amino-2-(hydroxymethyl)-1,3-propanediol, 4-hydroxyproline, adenosine, adipic acid, arachidonic acid, arginine, asparagine, azelaic acid, benzoate, beta-alanine(minor), capric acid, cholesterol, citraconate, creatinine, cysteine, fructose, galactonate gamma-lactone, glutamate, glutamine, glutarate, glycerol, glycine, glycolate,

								heptadecanoic acid 2, homoserine, hypoxanthine, inositol stereoisomer, isoleucine, leucine, lysine minor, malonate, methionine, N-actylglycine, octanedioic acid, oleic acid, palmitic acid, parabanic acid, Pelargonic acid, phenylalanine, phosphothreonine, pimelate, proline, serine, threonine, uracil, valine
Tadano, T. [62]2006	Case-control study	Serum samples were collected from 22 healthy adults and 20 cancer patients. Feces Samples were collected from 20 healthy adults and 20 cancer patients	Non-matched	Hospital	Feces, serum	Japan	GC-MS	Allolithocholic acid

Note, *six studies [2, 20, 39, 40, 42, 45] used a two-stage case-control design to validate biomarkers in biofluid.

#two studies [9, 34] of metabolic profiling analyses detected in tissue and serum/fecal samples.

Reference:

1. Djukovic D, Zhang J, Raftery D. Colorectal Cancer Detection Using Targeted LC-MS Metabolic Profiling. Methods Mol Biol 2018;1765:229-240.
2. Farshidfar F, Kopciuk KA, Hilsden R, et al. A quantitative multimodal metabolomic assay for colorectal cancer. BMC Cancer 2018;18:26.
3. Shu X, Xiang YB, Rothman N, et al. Prospective study of blood metabolites associated with colorectal cancer risk. Int J Cancer 2018.
4. Venalainen MK, Roine AN, Hakkinen MR, et al. Altered Polyamine Profiles in Colorectal Cancer. Anticancer Res 2018;38:3601-3607.
5. Chen C, Nagana Gowda GA, Zhu J, et al. Altered metabolite levels and correlations in patients with colorectal cancer and polyps detected using seemingly unrelated regression analysis. Metabolomics 2017;13.
6. Guertin KA, Li XS, Graubard BI, et al. Serum Trimethylamine N-oxide, Carnitine, Choline, and Betaine in Relation to Colorectal Cancer Risk in the Alpha Tocopherol, Beta Carotene Cancer Prevention Study. Cancer Epidemiol Biomarkers Prev 2017;26:945-952.
7. Jing Y, Wu X, Gao P, et al. Rapid differentiating colorectal cancer and colorectal polyp using dried blood spot mass spectrometry metabolomic approach. IUBMB Life 2017;69:347-354.
8. Kinross J, Mirnezami R, Alexander J, et al. A prospective analysis of mucosal microbiome-metabonome interactions in colorectal cancer using a combined MAS 1HNMR and metataxonomic strategy. Sci Rep 2017;7:8979.
9. Mika A, Kobiela J, Czumaj A, et al. Hyper-Elongation in Colorectal Cancer Tissue - Cerotic Acid is a Potential Novel Serum Metabolic Marker of Colorectal Malignancies. Cell Physiol Biochem 2017;41:722-730.
10. Ning W, Li H, Meng F, et al. Identification of differential metabolic characteristics between tumor and normal tissue from colorectal cancer patients by gas chromatography-mass spectrometry. Biomed Chromatogr 2017;31.
11. Nishiumi S, Kobayashi T, Kawana S, et al. Investigations in the possibility of early detection of colorectal cancer by gas chromatography/triple-quadrupole mass spectrometry. Oncotarget 2017;8:17115-17126.
12. Shen S, Yang L, Li L, et al. A plasma lipidomics strategy reveals perturbed lipid metabolic pathways and potential lipid biomarkers of human colorectal cancer. Journal of Chromatography B 2017;1068-1069:41-48.
13. Uchiyama K, Yagi N, Mizushima K, et al. Serum metabolomics analysis for early detection of colorectal cancer. J Gastroenterol 2017;52:677-694.
14. Wang X, Wang J, Rao B, et al. Gut flora profiling and fecal metabolite composition of colorectal cancer patients and healthy individuals. Exp Ther Med 2017;13:2848-2854.
15. Wang Z, Lin Y, Liang J, et al. NMR-based metabolomic techniques identify potential urinary biomarkers for early colorectal cancer detection. Oncotarget 2017;8:105819-105831.
16. Zhang LJ, Chen B, Zhang JJ, et al. Serum polyunsaturated fatty acid metabolites as useful tool for screening potential biomarker of colorectal cancer. Prostaglandins Leukot Essent Fatty Acids 2017;120:25-31.

17. Yan L, Ma C, Liu C, et al. NMR-based fecal metabolomics fingerprinting as predictors of earlier diagnosis in patients with colorectal cancer. Oncotarget 2016;7:29454-29464.
18. Gao P, Zhou C, Zhao L, et al. Tissue amino acid profile could be used to differentiate advanced adenoma from colorectal cancer. Journal of Pharmaceutical & Biomedical Analysis 2016;118:349-355.
19. Yuan T, Xu T, Huang J, et al. Tissue Metabonomic Phenotyping for Diagnosis and Prognosis of Human Colorectal Cancer. Sci Rep 2016;6:20790.
20. Crotti S, Agnoletto E, Cancemi G, et al. Altered plasma levels of decanoic acid in colorectal cancer as a new diagnostic biomarker. Anal Bioanal Chem 2016;408:6321-8.
21. Farshidfar F, Weljie AM, Kopciuk KA, et al. A validated metabolomic signature for colorectal cancer: exploration of the clinical value of metabolomics. Br J Cancer 2016;115:848-57.
22. Sinha R, Ahn J, Sampson JN, et al. Fecal Microbiota, Fecal Metabolome, and Colorectal Cancer Interrelations. PloS one 2016;11:e0152126.
23. Zhang H, Qiao L, Li X, et al. Tissue metabolic profiling of lymph node metastasis of colorectal cancer assessed by 1H NMR. Oncol Rep 2016;36:3436-3448.
24. Amal H, Leja M, Funka K, et al. Breath testing as potential colorectal cancer screening tool. International Journal of Cancer 2016;138:229-236.
25. Williams MD, Zhang X, Park JJ, et al. Characterizing metabolic changes in human colorectal cancer. Analytical & Bioanalytical Chemistry 2015;407:4581-4595.
26. Zamani Z, Arjmand M, Vahabi F, et al. A Metabolic Study on Colon Cancer Using 1H Nuclear Magnetic Resonance Spectroscopy. Biochemistry Research International 2014;2014:348712.
27. Cross AJ, Moore SC, Boca S, et al. A prospective study of serum metabolites and colorectal cancer risk. Cancer 2014;120:3049-3057.
28. Goedert JJ, Sampson JN, Moore SC, et al. Fecal metabolomics: assay performance and association with colorectal cancer. Carcinogenesis 2014;35:2089.
29. Bae S, Ulrich CM, Neuhouser ML, et al. Plasma choline metabolites and colorectal cancer risk in the Women's Health Initiative Observational Study. Cancer Research 2014;74:7442.
30. Wang C, Ke C, Wang X, et al. Noninvasive detection of colorectal cancer by analysis of exhaled breath. Analytical & Bioanalytical Chemistry 2014;406:4757-4763.
31. Wang C, Li P, Lian A, et al. Blood volatile compounds as biomarkers for colorectal cancer. Cancer Biology & Therapy 2014;15:200-206.
32. Mirnezami R, Jiménez B, Li JV, et al. Rapid diagnosis and staging of colorectal cancer via high-resolution magic angle spinning nuclear magnetic resonance (HR-MAS NMR) spectroscopy of intact tissue biopsies. Annals of Surgery 2014;259:1138-1149.
33. Qiu Y, Cai G, Zhou B, et al. A distinct metabolic signature of human colorectal cancer with prognostic potential. Clinical Cancer Research An Official Journal of the American Association for Cancer Research 2014;20:2136.
34. Phua LC, Xiu PC, Koh PK, et al. Non-invasive fecal metabonomic detection of colorectal cancer. Cancer Biology & Therapy 2014;15:389-397.

35. Jiménez B, Mirnezami R, Kinross J, et al. 1H HR-MAS NMR spectroscopy of tumor-induced local metabolic "field-effects" enables colorectal cancer staging and prognostication. Journal of Proteome Research 2013;12:959.
36. Kim S, Lee S, Maeng YH, et al. Study of Metabolic Profiling Changes in Colorectal Cancer Tissues Using 1D 1 H HR-MAS NMR Spectroscopy. Bulletin of the Korean Chemical Society 2013;34:1467-1472.
37. Li F, Qin X, Chen H, et al. Lipid profiling for early diagnosis and progression of colorectal cancer using direct-infusion electrospray ionization Fourier transform ion cyclotron resonance mass spectrometry. Rapid Commun Mass Spectrom 2013;27:24-34.
38. Weir TL, Manter DK, Sheflin AM, et al. Stool microbiome and metabolome differences between colorectal cancer patients and healthy adults. Plos One 2013;8:e70803.
39. Tan B, Qiu Y, Xia Z, et al. Metabonomics Identifies Serum Metabolite Markers of Colorectal Cancer. Journal of Proteome Research 2013;12:3000.
40. Nishiumi S, Kobayashi T, Ikeda A, et al. A Novel Serum Metabolomics-Based Diagnostic Approach for Colorectal Cancer. Plos One 2012;7:e40459.
41. Chen JL, Fan J, Yan LS, et al. Urine Metabolite Profiling of Human Colorectal Cancer by Capillary Electrophoresis Mass Spectrometry Based on MRB. Gastroenterology Research and Practice,2012,(2012-12-02) 2012;2012:125890.
42. Cheng Y, Xie G, Chen T, et al. Distinct urinary metabolic profile of human colorectal cancer. Journal of Proteome Research 2012;11:1354-1363.
43. Mal M, Koh PK, Cheah PY, et al. Metabotyping of human colorectal cancer using two-dimensional gas chromatography mass spectrometry. Analytical & Bioanalytical Chemistry 2012;403:483-493.
44. Ma Y, Zhang P, Wang F, et al. An integrated proteomics and metabolomics approach for defining oncofetal biomarkers in the colorectal cancer. Annals of Surgery 2012;255:720.
45. Bertini I, Cacciatore S, Jensen BV, et al. Metabolomic NMR fingerprinting to identify and predict survival of patients with metastatic colorectal cancer. Cancer Research 2012;72:356.
46. Ong ES, Zou L, Li S, et al. Metabolic profiling in colorectal cancer reveals signature metabolic shifts during tumorigenesis. Molecular & Cellular Proteomics Mcp 2010.
47. Ma Y, Liu W, Peng J, et al. A pilot study of gas chromatograph/mass spectrometry-based serum metabolic profiling of colorectal cancer after operation. Molecular Biology Reports 2010;37:1403-1411.
48. Tessem MB, Selnæs KM, Sjursen W, et al. Discrimination of Patients with Microsatellite Instability Colon Cancer using 1H HR MAS MR Spectroscopy and Chemometric Analysis. Journal of Proteome Research 2010;9:3664-70.
49. Wang W, Feng B, Li X, et al. Urinary metabolic profiling of colorectal carcinoma based on online affinity solid phase extraction-high performance liquid chromatography and ultra performance liquid chromatography-mass spectrometry. Molecular Biosystems 2010;6:1947-1955.
50. Shureiqi I, Chen D, Day RS, et al. Profiling lipoxygenase metabolism in specific steps of colorectal tumorigenesis. Cancer Prev Res (Phila) 2010;3:829-38.

51. Qiu Y, Cai G, Su M, et al. Urinary metabonomic study on colorectal cancer. Journal of Proteome Research 2010;9:1627.
52. Chae YK, Kang WY, Kim SH, et al. Combining Information of Common Metabolites Reveals Global Differences between Colorectal Cancerous and Normal Tissues. Bulletin- Korean Chemical Society 2010.
53. Chan ECY, Koh PK, Mal M, et al. Metabolic Profiling of Human Colorectal Cancer Using High-Resolution Magic Angle Spinning Nuclear Magnetic Resonance (HR-MAS NMR) Spectroscopy and Gas Chromatography Mass Spectrometry (GC/MS). Journal of Proteome Research 2009;8:352-361.
54. Qiu Y, Cai G, Su M, et al. Serum Metabolite Profiling of Human Colorectal Cancer Using GC−TOFMS and UPLC−QTOFMS. Journal of Proteome Research 2009;8:4844.
55. Righi V, Durante C, Cocchi M, et al. Discrimination of healthy and neoplastic human colon tissues by ex vivo HR-MAS NMR spectroscopy and chemometric analyses. Journal of Proteome Research 2009;8:1859.
56. Hirayama A, Kami K, Sugimoto M, et al. Quantitative Metabolome Profiling of Colon and Stomach Cancer Microenvironment by Capillary Electrophoresis Time-of-Flight Mass Spectrometry. Cancer Research 2009;69:4918-4925.
57. Hsu WY, Chen TL, Lin WD, et al. Analysis of urinary nucleosides as potential tumor markers in human colorectal cancer by high performance liquid chromatography/electrospray ionization tandem mass spectrometry. Clinica Chimica Acta 2009;412:1861.
58. Mal M, Koh PK, Cheah PY, et al. Development and validation of a gas chromatography/mass spectrometry method for the metabolic profiling of human colon tissue. Rapid Communications in Mass Spectrometry 2009;23:487–494.
59. Monleón D, Morales JM, Barrasa A, et al. Metabolite profiling of fecal water extracts from human colorectal cancer. Nmr in Biomedicine 2009;22:342-8.
60. Piotto M, Moussallieh FM, Dillmann B, et al. Metabolic characterization of primary human colorectal cancers using high resolution magic angle spinning 1 H magnetic resonance spectroscopy. Metabolomics 2008;5:292-301.
61. Denkert C, Budczies J, Weichert W, et al. Metabolite profiling of human colon carcinoma – deregulation of TCA cycle and amino acid turnover. Molecular Cancer 2008;7:72.
62. Tadano T, Kanoh M, Matsumoto M, et al. Studies of serum and feces bile acids determination by gas chromatography-mass spectrometry. Rinsho Byori 2006;54:103-10.

S.Table 15. Quality assessment of studies included in the systematic review by QUADOMICS

	Item																
**First author[ref]Year**	1	2	3	4a	4b	5	6	7	8	9	10	11	12	13	14	15	16
**Djukovic, D. [1]2018**	Y	N/A	Y	Y	Y	Y	Y	Y	Y	Y	Y	Y	N	Y	N/A	N	N
**Farshidfar, F. [2]2018**	Y	N/A	Y	Y	Y	Y	Y	Y	Y	Y	Y	Y	N	Y	N/A	N	Y
**Shu, X.[3]2018**	Y	N/A	Y	Y	N	Y	Y	Y	?	?	Y	?	N	Y	N/A	N	N
**Venalainen, M.K. [4]2018**	Y	N/A	Y	Y	Y	Y	Y	Y	?	Y	Y	?	N	Y	N/A	N	N
**Chen, C. [5]2017**	Y	N/A	Y	N	Y	Y	Y	Y	Y	Y	Y	Y	N	Y	N/A	N	N
**Guertin, K.A.[6]2017**	Y	N/A	Y	Y	N	Y	Y	Y	?	Y	Y	?	N	Y	N/A	N	N
**Jing, Y.[7]2017**	Y	N/A	Y	Y	N	Y	Y	Y	Y	Y	Y	Y	N	Y	N/A	N	N
**Kinross, J.[8]2017**	Y	N/A	Y	Y	Y	Y	Y	Y	Y	Y	N	Y	N	Y	N/A	N	N
**Mika, A.[9]2017**	Y	N/A	Y	Y	Y	Y	Y	Y	Y	Y	Y	Y	N	Y	N/A	N	N
**Ning, W.[10]2017**	Y	N/A	Y	Y	Y	Y	Y	Y	Y	Y	Y	Y	N	Y	N/A	N	N
**Nishiumi, S.[11]2017**	Y	N/A	Y	Y	N	Y	Y	Y	Y	Y	Y	Y	N	Y	N/A	N	N
**Shen, S.[12]2017**	Y	N/A	Y	Y	N	Y	Y	Y	N	Y	Y	Y	N	Y	N/A	N	N
**Uchiyama, K.[13]2017**	Y	N/A	Y	Y	N	Y	Y	Y	N	Y	Y	Y	N	Y	N/A	N	N
**Wang, X.[14]2017**	Y	N/A	Y	Y	Y	Y	Y	Y	Y	Y	Y	Y	N	Y	N/A	N	N
**Wang, Z.[15]2017**	Y	N/A	Y	Y	Y	Y	Y	Y	N	Y	Y	Y	N	Y	N/A	N	N
**Zhang, L.J.[16]2017**	Y	N/A	Y	Y	Y	Y	Y	Y	N	Y	Y	Y	N	Y	N/A	N	N
**Yan, L.[17]2016**	Y	N/A	Y	Y	Y	Y	Y	Y	Y	Y	Y	Y	N	Y	N/A	N	N
**Gao, P.[18]2016**	Y	N/A	Y	N	N	Y	Y	Y	Y	Y	Y	N	N	Y	N/A	N	Y
**Yuan, T.[19]2016**	Y	N/A	Y	Y	N	Y	Y	Y	Y	Y	Y	Y	N	Y	N/A	N	N
**Crotti, S.[20]2016**	Y	N/A	Y	Y	Y	Y	Y	Y	Y	Y	Y	Y	N	Y	N/A	N	Y
**Farshidfar, F.[21]2016**	Y	N/A	Y	Y	N	Y	Y	Y	Y	Y	Y	Y	N	Y	N/A	N	N
**Sinha, R.[22]2016**	Y	N/A	Y	Y	Y	Y	Y	Y	?	Y	N	?	N	Y	N/A	N	N

	Item																
**First author[ref]Year**	1	2	3	4a	4b	5	6	7	8	9	10	11	12	13	14	15	16
**Zhang, H.[23]2016**	Y	N/A	Y	Y	Y	Y	Y	Y	Y	Y	Y	Y	N	Y	N/A	N	N
**Amal, H.[24]2016**	Y	N/A	Y	Y	Y	Y	Y	Y	Y	Y	Y	Y	N	Y	N/A	N	N
**Williams, M.D.[25]2015**	Y	N/A	Y	Y	Y	Y	Y	Y	Y	Y	Y	Y	N	Y	N/A	N	N
**Zamani, Z.[26]2014**	Y	N/A	Y	N	N	Y	Y	Y	?	Y	Y	Y	N	Y	N/A	N	N
**Cross, A.J.[27]2014**	Y	N/A	Y	Y	Y	Y	Y	Y	Y	Y	N	Y	N	Y	N/A	N	N
**Goedert, J.J.[28]2014**	Y	N/A	Y	Y	Y	Y	Y	Y	?	Y	N	Y	N	Y	N/A	N	N
**Bae, S.[29]2014**	Y	N/A	Y	Y	Y	Y	Y	Y	Y	Y	Y	?	N	Y	N/A	N	N
**Wang, C.[30]2014**	Y	N/A	Y	Y	Y	Y	Y	Y	Y	Y	Y	Y	N	Y	N/A	N	N
**Wang, C.[31] 2014**	Y	N/A	Y	Y	Y	Y	Y	Y	Y	Y	Y	Y	N	Y	N/A	N	N
**Mirnezami, R.[32]2014**	Y	N/A	Y	Y	Y	Y	Y	Y	Y	Y	Y	Y	N	Y	N/A	N	N
**Qiu, Y.[33]2014**	Y	N/A	Y	Y	Y	Y	Y	Y	Y	Y	Y	?	N	Y	N/A	N	N
**Phua, L.C.[34]2014**	Y	N/A	Y	Y	Y	Y	Y	Y	Y	Y	Y	Y	N	Y	N/A	N	N
**Jiménez, B.[35]2013**	Y	N/A	Y	Y	Y	Y	Y	Y	Y	Y	Y	Y	N	Y	N/A	N	N
**Kim, S.[36]2013**	Y	N/A	Y	N	N	Y	Y	Y	Y	Y	Y	Y	N	Y	N/A	N	N
**Li, F.[37]2013**	Y	N/A	Y	Y	?	Y	Y	Y	?	Y	Y	Y	N	Y	N/A	N	N
**Weir, T.L.[38]2013**	Y	N/A	Y	Y	Y	Y	Y	Y	N	Y	Y	?	N	Y	N/A	N	N
**Tan, B.[39]2013**	Y	N/A	Y	Y	Y	Y	Y	Y	?	Y	Y	?	N	Y	N/A	N	Y
**Nishiumi, S.[40]2012**	Y	N/A	Y	Y	Y	Y	Y	Y	Y	Y	Y	Y	N	Y	N/A	N	Y
**Chen, J.L.[41]2012**	Y	N/A	Y	Y	Y	Y	Y	Y	N	Y	Y	Y	N	Y	N/A	N	N
**Cheng, Y.[42]2012**	Y	N/A	Y	Y	Y	Y	Y	Y	?	Y	Y	Y	N	Y	N/A	N	Y
**Mal, M.[43]2012**	Y	N/A	Y	Y	Y	Y	Y	Y	Y	Y	Y	Y	N	Y	N/A	N	N
**Ma, Y.[44]2012**	Y	N/A	Y	N	N	Y	Y	Y	Y	Y	Y	?	N	Y	N/A	N	N
**Bertini, I.[45]2012**	Y	N/A	Y	?	Y	Y	Y	Y	N	Y	Y	?	N	Y	N/A	N	Y
**Ong, E.S.[46]2010**	Y	N/A	Y	Y	Y	Y	Y	Y	Y	Y	Y	Y	N	Y	N/A	N	N

First author[ref]Year	Item																
	1	2	3	4a	4b	5	6	7	8	9	10	11	12	13	14	15	16
**Ma, Y.[47]2010**	Y	N/A	Y	Y	Y	Y	Y	Y	Y	Y	Y	Y	N	Y	N/A	N	N
**Tessem, M.B.[48]2010**	Y	N/A	Y	Y	N	Y	Y	Y	Y	Y	Y	?	N	Y	N/A	N	N
**Wang, W.[49]2010**	Y	N/A	Y	N	N	Y	Y	Y	N	Y	Y	?	N	Y	N/A	N	N
**Shureiqi, I.[50]2010**	Y	N/A	Y	Y	Y	Y	Y	Y	Y	Y	Y	Y	N	Y	N/A	N	N
**Qiu, Y.[51]2010**	Y	N/A	Y	Y	Y	Y	Y	Y	N	Y	Y	Y	N	Y	N/A	N	N
**Chae, Y.K.[52]2010**	Y	N/A	Y	Y	Y	Y	Y	Y	Y	Y	Y	Y	N	Y	N/A	N	N
**Chan, E.C.Y.[53]2009**	Y	N/A	Y	Y	Y	Y	Y	Y	Y	Y	Y	?	N	Y	N/A	N	N
**Qiu, Y.[54]2009**	Y	N/A	Y	Y	N	Y	Y	Y	N	Y	Y	?	N	Y	N/A	N	N
**Righi, V.[55]2009**	Y	N/A	Y	Y	Y	Y	Y	Y	Y	Y	Y	Y	N	Y	N/A	N	N
**Hirayama, A.[56]2009**	Y	N/A	Y	Y	N	Y	Y	Y	Y	Y	Y	N	N	Y	N/A	N	N
**Hsu, W.Y.[57]2009**	Y	N/A	Y	Y	Y	Y	Y	Y	?	Y	Y	?	N	Y	N/A	N	N
**Mal, M.[58]2009**	Y	N/A	Y	N	N	Y	Y	Y	Y	Y	Y	?	N	Y	N/A	N	N
**Monleón, D.[59]2009**	Y	N/A	Y	N	Y	Y	Y	Y	Y	Y	Y	?	N	Y	N/A	N	N
**Piotto, M.[60]2008**	Y	N/A	Y	Y	Y	Y	Y	Y	Y	Y	Y	Y	N	Y	N/A	N	N
**Denkert, C.[61]2008**	Y	N/A	Y	Y	N	Y	Y	Y	Y	Y	Y	Y	N	Y	N/A	N	N
**Tadano, T.[62]2006**	Y	N/A	Y	N	N	Y	Y	Y	?	Y	Y	?	N	Y	N/A	N	N
**YES%**	100	0	100	83.87	67.74	100	100	100	66.13	98.39	93.55	67.74	0	100	0	0	11.29
**NO%**	0	0	0	14.52	30.65	0	0	0	16.13	0	0	3.23	100	0	0	100	88.71
**UC%**	0	0	0	1.61	1.61	0	0	0	17.74	1.61	6.45	29.03	0	0	0	0	0
**N/A%**	0	100	0	0	0	0	0	0	0	0	0	0	0	0	100	0	0

Note, the reference numbers are the same as in Supplementary Table 12.

Index: Y=criteria achieved, N=criteria not achieved, ?=Unclear, N/A=not applicable.

Item 1. Were selection criteria clearly described?

2. Was the spectrum of patients' representative of patients who will receive the test in practice?

3. Was the type of sample fully described?

4. Were the procedures and timing of biological sample collection with respect to clinical factors described with enough detail?

4a. Clinical and physiological factors; 4b. Diagnostic and treatment procedures

5. Were handling and pre-aN/Alytical procedures reported in sufficient detail and similar for the whole sample? And, if differences in procedures were reported, was their effect on the results assessed?

6. Is the time period between the reference standard and the index test short enough to reasoN/Ably guarantee that the target condition did not change between the two tests?

7. Is the reference standard likely to correctly classify the target condition?

8. Did the whole sample or a random selection of the sample receive verification using a reference standard of diagnosis?

9. Did patients receive the same reference standard regardless of the result of the index test?

10. Was the execution of the index test described in sufficient detail to permit replication of the test?

11. Was the execution of the reference standard described in sufficient detail to permit its replication?

12. Were the index test results interpreted without knowledge of the results of the reference standard?

13. Were the reference standard results interpreted without knowledge of the results of the index test?

14. Were the same clinical data available when test results were interpreted as would be available when the test is used in practice?

15. Were uninterpretable/intermediate test results reported?

16. Is it likely that the presence of over-fitting was avoided?

S.Table 16 Limit of detection, limit of quantification and standard curve of standards

Compound name	Equation of regression	R	Linearity range（μg/ml）	Quantitation limit（μg/ml）	Detection limit（μg/ml）
L-Tryptophon	y = 13.374x + 3.925	0.9979	1.0~12.0	0.1	0.05
Linoleic Acid	y = 29.038x - 1.791	0.9998	20~120	0.1	0.05
LysoPC(14:0)	y = 187.47x + 4.545	0.9932	0.1~1.2	0.01	0.005
LysoPC(16:0)	y = 51.207x + 301.25	0.9968	5.0~25.0	0.01	0.005
LysoPC(18:0)	y = 52.405x + 374	0.9929	5.0~25.0	0.05	0.01

S.Table 17 Standard addition recovery and relative standard deviation (N =6)

Compound name	1μg/mL		5μg/mL		10μg/mL	
	Recovery /%	RSD /%	Recovery /%	RSD /%	Recovery /%	RSD /%
L-Tryptophon	95.8	8.4	90.1	11.6	88.1	6.6
Linoleic Acid	93.6	10.8	91.5	14.1	86.9	7.0
LysoPC(14:0)	92.4	10.2	93.7	5.3	87.4	7.9
LysoPC(16:0)	91.4	8.5	89.3	6.0	86.1	8.4
LysoPC(18:0)	94.8	8.4	89.5	11.3	87.9	7.8

S.Table 18 Intra-day and inter-day precision (n=6)

Compound name	1μg/mL		5μg/mL		10μg/mL	
	**Intra-day** (%)	**Inter-day** (%)	**Intra-day** (%)	**Inter-day** (%)	**Intra-day** (%)	**Inter-day** (%)
L-Tryptophon	4.6	6.1	4.3	7.2	4.1	5.5
Linoleic Acid	6.0	12.1	6.4	9.9	4.1	10.7
LysoPC(14:0)	6.7	10.5	4.9	7.8	7.6	11.4
LysoPC(16:0)	6.9	13.8	5.9	9.1	5.5	8.3
LysoPC(18:0)	4.2	6.9	4.4	7.1	3.6	5.7

S.Table 19 The sensitivity and specificity of metabolites in ROC analysis

	First stage		Second stage		Paired-tissue		Targeted	
Metabolites	sensitivity	specificity	sensitivity	specificity	sensitivity	specificity	sensitivity	specificity
13-OxoODE	0.848	0.838	0.577	0.803	0.431	0.922		
2-Aminobenzoic acid	0.939	0.706	0.865	0.535				
2'-Deoxyinosine triphosphate	0.833	0.838	0.625	0.906				
3a,7a-Dihydroxycholanoic acid	0.939	0.897	0.923	0.913				
Alpha-N-Phenylacetyl-L-glutamine	0.909	0.926	0.856	0.929				
Citric acid	0.909	0.809	0.962	0.488				
Eicosenoic acid	1.000	1.000	0.990	0.764				
Glycochenodeoxycholate	0.712	0.632	0.962	0.425	0.804	0.863		
Glycocholic acid	0.727	0.544	0.394	0.874	0.725	0.922		
IDP	0.924	0.647	0.99	0.378	0.588	0.922		
Inosine	0.697	0.794	0.519	0.803				
Linoleic acid	0.682	0.868	0.625	0.677	0.667	0.804	0.490	0.684
L-Phenylalanine	0.485	0.824	0.721	0.559				
L-Tryptophan	0.727	0.706	0.740	0.685	0.608	0.922	0.566	0.597
LysoPC(14:0)	0.894	0.765	0.875	0.795			0.649	0.715
LysoPC(16:0)	0.848	0.897	0.827	0.827	0.725	0.863		
LysoPC(16:1(9Z))	0.924	0.926	0.913	0.866				
Taurocholic acid	0.742	0.794	0.817	0.693				
LysoPC(18:0)							0.426	0.787
Panel_1	0.970	0.956	0.962	0.858	0.902	0.863		
Panel_2	0.939	0.897	0.856	0.945				
Panel_3	0.924	0.897	0.933	0.811	0.902	0.941		
Combined*							0.790	0.589

*The combined results of metabolites in targeted quantitative analysis.
